# ﻿A revision of the four Afrotropical and Palaearctic *Sphyracephala* Say (Diptera, Diopsidae) with an illustrated overview of the other five *Sphyracephala*

**DOI:** 10.3897/zookeys.1241.151490

**Published:** 2025-06-10

**Authors:** Hans R. Feijen, Frida A. A. Feijen, Cobi Feijen

**Affiliations:** 1 Naturalis Biodiversity Center, P. O. Box 9517, 2300 RA Leiden, Netherlands Naturalis Biodiversity Center Leiden Netherlands; 2 ETH Zürich, Department of Environmental Systems Science, Institute of Integrative Biology (IBZ), 8092 Zürich, Switzerland Institute of Integrative Biology Zürich Switzerland; 3 EAWAG, Aquatic Ecology, Dübendorf, Switzerland EAWAG, Aquatic Ecology Dübendorf Switzerland

**Keywords:** Allometry, biogeography, key, redescriptions, *Sphyracephala*, stalk-eyed flies, synonymy, wing morphometrics

## Abstract

In the Afrotropical Region, *Sphyracephalabeccarii* (Rondani) and *S.munroi* Curran are found, the former just extending into the Palaearctic Region. In the latter region, *S.babadjanidesi* Zaitzev occurs in the Balkan and Caucasus Regions and *S.nigrimana* Loew in Far Eastern Russia and North-Eastern China. The European stalk-eyed fly *S.europaea* Papp & Földvári is proposed as junior synonym of the Eurasian stalk-eyed fly *S.babadjanidesi*. In North America *S.brevicornis* (Say) and *S.subbifasciata* Fitch occur. The four true Holarctic *Sphyracephala* are shown to reach their northern limits between 45°30'N and 48°20'N. These species hibernate and show characteristic clustering behaviour in spring and autumn. The four Palaearctic and Afrotropical *Sphyracephala* are redescribed and extensively illustrated. A key is given to all nine *Sphyracephala* presently recognised. The subdivisions within the genus are discussed. A cladogram, based on morphological, molecular, wing morphometric and allometric considerations, is presented. It shows the two species groups recognised. Each species group is divided into two subtaxa. Geometric morphometric analysis supports the grouping in four subtaxa as well as the synonymy of *S.babadjanidesi* and *S.europaea*. In *Sphyracephala*, both sexual monomorphism and dimorphism with relation to eye span occur. The allometric lines for males and females of eight species are compared. A clear link is found between allometric slopes and the four subtaxa distinguished. In *Sphyracephala*, female-biased, balanced, and male-biased sex ratios are found. A remarkable case of female-biased sex ratio distortion is reported for *Sphyracephalabeccarii*. In Continental Africa and the Arabian Peninsula, a balanced sex ratio was found, while in Madagascar a female-biased 2:1 ratio was found. This represents the first case of a female-biased sex-ratio in a geographically isolated population of a monomorphic diopsid.

## ﻿Introduction

The genus *Sphyracephala* was erected by Say (1828) for the Nearctic *Diopsisbrevicornis* Say, 1817. Westwood (1845) described a species from India as *Diopsishearseiana*, but later (1848) referred it to *Sphryracephala* [sic]. Rondani (1875) erected *Zygocephala* for *S.hearseiana* but that genus soon reverted to *Sphyracephala* (Brunetti 1907). The second Nearctic species was described by Fitch (1855) as *Sphyracephalasubbifasciata*. That species was for a long time considered a junior synonym of *S.brevicornis*, but reinstated as a true species by Feijen (1989). Loew (1873) described the first Palaearctic species from far eastern Russia as *Sphyracephalanigrimana*, while Zaitzev (1919) added as second Palaearctic species *Sphyracephalababadjanidesi*.

Oriental *Sphyracephala* species were described by Walker (1860) from Sulawesi as *Diopsisdetrahens* (= *Diopsis* (*Sphryracephala* [sic]) *cothurnata*Bigot 1874) and by Senior-White (1922) as *Teleopsisbipunctipennis* from Sri Lanka. Hendel (1917) erected the genus *Pseudodiopsis* for *D.cothurnata*. Shillito (1940) and Steyskal (1977) referred *T.bipunctipennis* to *Pseudodiopsis*. Rondani (1873) added the first Afrotropical species as *Diopsisbeccarii*, and subsequently (1875) referred it to his short-lived *Hexechopsis* Rondani. Osten Sacken (1882) referred *D.beccarii* to *Sphyracephala*. Karsch (1888) described the Afrotropical *Sphyracephalaafricana*, while Curran (1928) added *Sphyracephalamunroi*. Collart (1954) reviewed the Afrotropical *Sphyracephala* and tabulated the differences between *S.beccarii* and *S.munroi*. He confirmed the earlier assumed (Curran 1928, Séguy 1949) synonymy of *S.africana* with *S.beccarii*.

Hennig (1941b) reviewed the Palaearctic species of *Sphyracephala*. He included three species: *S.nigrimana*, *S.babadjanidesi* and *S.beccarii*. Hennig improved the description for *S.nigrimana*, provided some illustrations and extended the distribution from the Amur Oblast in the Russian Far East to northern China. The description for *S.babadjanidesi* from Azerbaijan was translated from Russian and English into German. Hennig considered the *Sphyracephalahearseiana* Westwood record from Algeria by Bezzi (1922) and assumed it to represent *S.beccarii*. This view was confirmed in Vaillant (1953). Hennig added to the description of the largely Afrotropical *S.beccarii* and illustrated its epandrium.

*Sphyracephala* was reviewed by Feijen (1989) who placed *Pseudodiopsis* Hendel, 1917 (= *Microdiopsis* Curran, 1934, as indicated by Curran in his corrections) in synonymy with *Sphyracephala*. Feijen revised the Nearctic *Sphyracephala*, presented a catalogue of *Sphyracephala* and a provisional diagnosis. Based on morphological characters, Feijen divided the *Sphyracephala* into three species groups. The first group was called the *S.brevicornis* species group and included the Nearctic *S.brevicornis* and *S.subbifasciata* together with the Palaearctic *S.babadjanidesi* and *S.nigrimana*. The second group, the *S.hearseiana* species group included the Oriental *S.hearseiana* and the Afrotropical *S.beccarii* and *S.munroi*. The third species group included the Oriental species previously placed in *Pseudodiopsis* and referred to as *S.detrahens* and *S.bipunctipennis*. The second and the third groups were considered sister groups.

Molecular analyses of *Sphyracephala* species were given by Baker (1999), Meier and Baker (2002), and Jackson (2019). They confirmed the view that *Pseudodiopsis* is embedded in *Sphyracephala*. Jackson considered six species and indicated two main taxa in *Sphyracephala*. The first one included *S.beccarii* with as sister taxon *S.bipunctipennis* together with *S.detrahens*. The second one included *S.europaea* Papp and Földvári with as sister taxon *S.brevicornis* together with *S.munroi*. This agrees with the groups proposed by Feijen (1989) with the placement of *S.munroi* as exception. Although this paper is not a revision of *Sphyracephala*, the intrageneric phylogeny will be discussed.

The latest *Sphyracephala* described so far, is *S.europaea* Papp & Földvári (Papp et al. 1997) found near the Tisza River in southern Hungary. It is remarkable that the presence of *Sphyracephala* in Europe went unnoticed till 1997, while the first American, Asian, and African *Sphyracephala* species were, respectively, described by Say (1817), Westwood (1845), and Rondani (1873). *Sphyracephalaeuropaea* is, of course, rather small but the eye stalks are striking, while clustering in large numbers appears common (Paulovics 1998; Simova-Tošić and Stojanović 2000; Kutsarov and Hubenov 2019) as in other *Sphyracephala* (see Feijen et al. 2017). For Europe, it is the more surprising that *S.europaea* was subsequently and in quick order found in Serbia (Simova-Tošić and Stojanović 2000), on the border of Hungary and Romania (KMNP 2018) and on the border of Bulgaria and Romania (Kutsarov and Hubenov 2019).

Hauser et al. (2011) and Feijen et al. (2017) showed that *S.beccarii* also extends into the Palaearctic section of the Arabian Peninsula. Hilger (2000) and Feijen et al. (2017) discussed other Diopsidae occurring close to the Palaearctic region. The two Afrotropical *Sphyracephala*, *S.beccarii* and *S.munroi*, and the three Palaearctic species, *S.babadjanidesi*, *S.nigrimana*, and (just extending into the region) *S.beccarii* will be reviewed and illustrated. The status of *S.europaea* will be considered leading to the recommendation to designate it a junior synonym of *S.babadjanidesi*. The five *Sphyracephala* from other regions will be listed and illustrated.

## ﻿Materials and methods

Details on procedures for preparing genitalia slides and procedures for taking measurements are given in Feijen et al. (2018). For information on morphological terminology, the reader is referred to the same source. The following categories of rate of dimorphism *D* are used: monomorphy -0.25–0.25, very low *D* 0.26–0.50, low *D* 0.51–1.00, moderate *D* 1.01–2.00, high *D* 2.01–3.00, and very high *D* > 3.00. For focus stacking photography of specimens, a Zeiss Stereomicroscope SteREO Discovery.V20 was used. Wings were mainly photographed while mounted in slides. The distribution map was built using the online version of SimpleMappr (Shorthouse 2010).

Geometric morphometric analysis was used to find which species are similar according to wing venation geometry. Single wing photographs of 31 specimens were used: five for *S.babadjanidesi* (among which two *S.europaea* paratype wings), five *S.beccarii*, two *S.bipunctipennis*, two *S.brevicornis*, three *S.detrahens*, four *S.hearseiana*, five *S.munroi*, two *S.nigrimana*, and three *S.subbifasciata*. Photos were imported in tpsDig 2.32 (Rohlf 2021) and a similar set of landmarks was used as for the genus *Madagopsina* (Feijen et al. 2018). Exceptions are the addition of a landmark at the base of vein CuA+CuP, and the replacement of the landmark at the tip of M4 by a landmark on the tangent from the tip of M1 with cell bm+dm (see figure in discussion). This last modification was necessary since vein M4 is not extending beyond crossvein dm-m in all *Sphyracephala* (see figure in discussion). Landmark data were imported using R v. 4.4.2 in RStudio 2024.09.0 (R Core Team 2024; Posit team 2024). Analysis and data visualization proceeded using the geomorph v. 4.0.9 (Adams et al. 2025; Baken et al. 2021), pvclust v. 2.2-0 (Suzuki and Shimodaira 2019) and ggplot2 v. 3.5.1 (Wickham 2016) packages. Generalized Procrustes transformation of the raw landmark coordinates was done using the gpagen function. Principal Components Analysis (PCA) of the transformed data was done using the gm.prcomp function. Finally, hierarchical clustering dendrograms were built using the pvclust function on the PCA scores. The pvclust function calculates the AU (Approximately Unbiased) p-value and BP (Bootstrap Probability) value for branches in the dendrogram, thus enabling an interpretation of the robustness of the hierarchical clustering analysis. Default function settings were used in all steps of the analysis, except for increasing the number of bootstraps of the hierarchical clustering analysis to 10.000 and using both the average and complete agglomeration methods in the hierarchical clustering analysis.

The following institutional codens and abbreviations are used:

**AMGS**Albany Museum, Grahamstown, Cape Province, South Africa;

**BMSA**National Museum Bloemfontein, Bloemfontein, South Africa;

**CAS**California Academy of Sciences, San Francisco, California, USA;

**CSCA**California State Collection of Arthropods, Sacramento, California, USA;

**FBUB**Universität Bielefeld, Bielefeld, Germany;

**HNHM**Hungarian Natural History Museum, Budapest, Hungary;

**MLUH**Wissenschaftsbereich Zoologie, Martin-Luther-Universität, Halle (Saale), Germany;

**MSNG**Museo Civico di Storia Naturale “Giacomo Doria”, Genova, Italy;

**NHMBEO** Natural History Museum, Belgrade, Serbia;

**NHMUK**Natural History Museum, London, United Kingdom;

**NHRS**Naturhistoriska Riksmuseet, Stockholm, Sweden;

**NMSA**KwaZulu-Natal Museum, Pietermaritzburg, South Africa;

**RMNH**Naturalis Biodiversity Center (formerly Rijksmuseum van Natuurlijke Historie), Leiden, The Netherlands;

**SMF**Forschungsinstitut und Naturmuseum Senckenberg, Frankfurt am Main, Germany;

**SOFM**National Museum of Natural History, Sofia, Bulgaria;

**USNM**National Museum of Natural History (formerly United States National Museum), Washington D.C., USA;

**WVUC**West Virginia University, Morgantown, West Virginia, USA;

**ZIN**Russian Academy, of Sciences, Zoological Institute, St. Petersburg, Russia;

**ZMHB** Museum für Naturkunde der Humboldt-Universität, Berlin, Germany;

**ZSM**Zoologische Staatssammlung, München, Germany.

**AU** Approximately Unbiased p-value (%)

**BP** Bootstrap Probability values (%)

**D** Rate of dimorphism

**l/w** (ratio) length/width

**PCA** Principal Components Analysis

**sc. sp.** scutellar spine

**SE** Standard Error

## ﻿Taxonomy

### ﻿Family Diopsidae

Taxon classificationAnimaliaDipteraDiopsidae

Billberg, 1820

E6C2C0E0-9F42-58B0-97F9-5A7793C0ADEC

Diopsidae : Billberg, 1820: 115 (as Natio Diopsides).

#### Type genus.

*Diopsis* Linnaeus, 1775: 5.

### ﻿Genus Sphyracephala

Taxon classificationAnimaliaDipteraDiopsidae

Say, 1828

B83ACA91-AEAD-5B55-A644-F4182DB64D3D

Sphyracephala Say, 1828: plate 52. Osten Sacken 1882: 234 (morphology, catalogue); Brunetti 1907: 163 (Oriental, clustering); Bezzi 1922: 69 (world catalogue); Sen 1921: 33 (Oriental, ecology); Curran 1928: 274 (Afrotropical); Collart 1954: 329 (Afrotropical); Hennig 1941a: 59, 1941b: 3 (Palaearctic, morphology), 1965: 54 (phylogeny); Séguy 1955: 1123 (Afrotropical); Descamps 1957: 19 (Afrotropical, biology); van Bruggen 1961: 425 (Afrotropical); Lavigne 1962: 5 (Nearctic, biology); Steyskal 1972: 13 (world catalogue), 1977: 35 (Oriental catalogue); Hochberg-Stasny 1985: 1 (Nearctic, biology); Peterson 1987: 785 (Nearctic, morphology); Feijen 1989: 66 (Nearctic and world, morphology); Papp et al. 1997: 137 (Palaearctic); Hilger 2000: 335 (Palaearctic, morphology); Meier and Hilger 2000: 6 (egg morphology); Meier and Baker 2002: 329 (phylogeny); O’Hara et al. 2011: 96, 191 (synonyms); Feijen et al. 2017: 76 (Arabian Peninsula, biogeography, clustering); Nartshuk 2017: 128 (Palaearctic); Feijen and Feijen 2019: 39 (Oriental); Jackson 2019: suppl. figs 1, 2 (phylogeny).
Sphryracephala , Westwood 1848: 37, pl. 18 fig. 3. Portschinsky 1871: 287; Bigot 1874: 115; Sen 1921: 33. (Error for Sphyracephala).
Hexechopsis Rondani, 1875: 442, type-species Diopsisbeccarii Rondani, 1873, by original designation and monotypy. Osten Sacken 1882: 235; Brunetti 1907: 163; Eggers 1916: 27; 1925: 482; Bezzi 1922: 71 (as Hexecopsis); Shillito 1940: 148; Hennig 1941b: 5; Séguy 1949: 74; van Bruggen 1961: 425; Steyskal 1972: 13; Cogan and Shillito 1980: 584; Feijen 1989: 66; Feijen et al. 2017: 76.
Zygocephala Rondani, 1875: 443, type-species Diopsishearseiana Westwood, 1845, by original designation and monotypy (as Diopsishearsejana (Wiedemann)). Osten Sacken 1882: 235 (implicitly); Brunetti 1907: 163; Shillito 1940: 148; van Bruggen 1961: 425; Steyskal 1972: 13, 1977: 35; Feijen 1989: 66; Feijen and Feijen 2019: 40.
Pseudodiopsis Hendel, 1917: 33, type-species Sphyracephalacothurnata Bigot, 1874, by original designation and monotypy. Malloch 1938: 437; Shillito 1940: 150; Hennig 1965: 62 (in Diopsini); Steyskal 1972: 12 (in Sphyracephalini); Feijen 1989: 66 (as synonym of Sphyracephala); Burkhardt and de la Motte 1996: 173; Baker 1999: 24, figs 1, 2 (as synonym); Meier and Hilger 2000: 32 (as synonym); Baker et al. 2001: 93, fig. 1; Andersen 2001: 138; Meier and Baker 2002: 334; Jackson 2019: suppl. fig. 1.
Sphyrocephala [sic]: Curran 1934: 358. Flint 1956: 44; Stone 1980: 17.
Microdiopsis Curran, 1934: 359, type-species Sphyracephalacothurnata Bigot, 1874, by original designation and monotypy. Curran 1934: 495 (in the corrections); Malloch 1938: 437; Shillito 1940: 148; Steyskal 1972: 12; Arnaud and Owen 1981: 144.
Sphracephala [sic]: Nayar and Tandon 1962a: 113, 1962b: 132, 1963: 1; Singh et al. 1962: 79.

#### Type species.

*Diopsisbrevicornis* Say, 1817: 23, by monotypy.

**Figures 1, 2.**
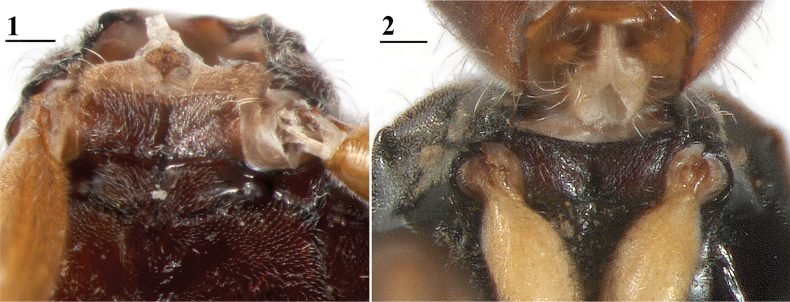
Prosternum, ventral view **1** basiliform prosternum, ♂, *Sphyracephalamunroi*, Arusha, Tanzania **2** precoxal bridge, ♂, *Sphyracephalabeccarii*, Mboma, DR Congo. Scale bars: 0.1 mm.

**Figures 3, 4.**
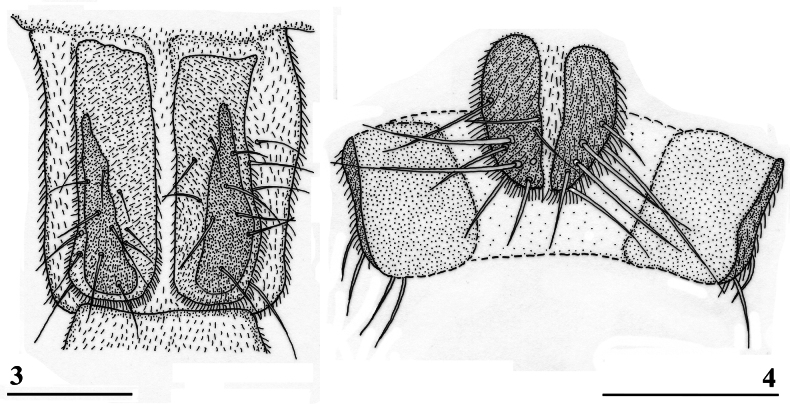
♀, sternite 8, ventral view **3***Sphyracephalamunroi*, Arusha, Tanzania **4***Sphyracephalabeccarii*, Maputo, Mozambique. Scale bars: 0.1 mm.

##### ﻿Key to the *Sphyracephala*

Although this revision concentrates on the Afrotropical and Palaearctic *Sphyracephala*, the key covers all described species. It should be stressed that in the Oriental and Australasian Regions some species remain to be described. *Sphyracephaladetrahens* and *S.bipunctipennis* also need to be redescribed, so couplet 8 will then be updated and extended.

**Table:** 

1	Basiliform prosternum (Fig. 1); tergite 1 with semicircular groove; ♀ sternite 8 with 2 small sclerites located on the meson Fig. 3); presence of sclerotised ring of ventral vagina wall; surstylus without microtrichia. *Sphyracephalabrevicornis* species group	**2**
–	Precoxal bridge (Fig. 2); tergite 1 with two longitudinal grooves; ♀ sternite 8 with 2 large, rectangular, plates, well separated on the meson Fig. 4); absence of sclerotised ring of ventral vagina wall; surstylus with microtrichia on outer side. *Sphyracephalahearseiana* species group	**6**
2	Distinct wing markings (Figs 82, 116) including apical wing spot and central wing band running from vein R1 to posterior margin; eye span/body ratio: 0.38–0.44; sexual monomorphy with regard to eye span (D = 0.01–0.04); scutellar spine/scutellum ratio: 0.54–0.75; apical seta/scutellar spine ratio: 2.8–3.9	**3**
–	None or vague wing markings (Figs 12, 59); if markings present, central spot running from below vein R2+3 to posterior end of crossvein dm-m; eye span/body ratio: 0.52–0.67; sexual dimorphy with regard to eye span (D = 0.33–0.39); scutellar spine/scutellum ratio: 0.41–0.44; apical seta/scutellar spine ratio: 5.4–6.1	**5**
3	Central wing band proximally extending to crossvein r-m and from there to vein R1 creating pale anterior wing spot (Fig. 82); inner side of fore femur brown with darker apical half; surstyli articulate, elongate (Figs 94, 95, l/w ratio: 4.7); Far Eastern Siberia, North-Eastern China	***Sphyracephalanigrimana***
–	Central wing band proximally just extending to crossvein r-m (Figs 116, 121); inner side of fore femur almost uniformly blackish brown or brown with broad transverse darker band; surstyli fused to epandrium; surstyli oblong (l/w ratio: ~ 1.9–2.2); Canada, USA	**4**
4	Distinct apical wing spot, vague infuscation posterior to base of subcostal cell (Fig. 116); fore femur with two rows of spinous bristles, uniformly blackish brown with pale base and apex; l/w ratio of fore femur ~ 2.66; surstylus oblong in lateral view	***Sphyracephalabrevicornis***
–	Only slight infuscation at wing apex, distinct spot posterior to base of subcostal cell (Fig. 121); fore femur with one row of spinous bristles, yellowish brown with brown spots; l/w ratio of fore femur ~ 2.43; surstylus oblong to spatula-shaped (depending on angle of view) in lateral view	***Sphyracephalasubbifasciata***
5	Overall clothed in sparse, small white setulae; small eye span (1.7–2.2 mm) in ♀ and ♂ (respectively, ~ 52% and ~ 60% of body lengths); wing almost transparent with vague brown central and apical spots; l/w ratio of fore femur 2.7–2.9; inner side of fore femur with centrally a broad, dark transverse band; ♀ cerci rather elongate, l/w ratio: ~ 3.2, remarkably curled upward; surstyli rectangular, l/w ratio: ~ 1.5–1.7; Balkan and Caucasus regions	***Sphyracephalababadjanidesi***
–	Overall clothed in long dark setulae; small eye span (2.5–2.7 mm) in ♀ and ♂ (respectively ~ 61% and ~ 67% of body length); wing transparent, without spots; l/w ratio of fore femur 3.4–3.9; inner side of fore femur with dark fine, longitudinal stripe on central third; ♀ cerci elongate, l/w ratio: ~ 4.6, not curled upward; surstyli elongate, l/w ratio: ~ 2.8; Afrotropical Region	***Sphyracephalamunroi***
6	Large inner vertical setae; transparent wing without spots; vein M4 normally extending beyond crossvein dm-m; pale, whitish scutellar spines; scutellar spine/scutellum ratio: 0.48–0.56; apical seta/scutellar spine ratio: 3.60–3.91; articulate surstyli	**7**
–	Inner vertical setae absent; wing with large brown apical wing spot (~ 40% of wing length) and large central spot; vein M4 not extending beyond crossvein dm-m; dark scutellar spines; scutellar spine/scutellum ratio: 0.88–1.15; apical seta/scutellar spine ratio: 2.50–2.93; surstyli fused to epandrium with suture visible	**8**
7	Epandrium semi-circular with square posterior corners (Fig. 132); surstyli posteriorly directed, slender, sickle-shaped, strongly tapering towards apex (Fig. 133); sternite 7 with peculiar small invagination anteromedially (see Kumar 1978b: fig. 2); ♀ cercus with l/w ratio: ~ 2.5	***Sphyracephalahearseiana***
–	Epandrium almost circular (Fig. 48); surstyli medially directed, apices almost touching, apically acute with an upturned apex (Fig. 49); ♀ sternite 7 without invagination anteromedially; ♀ cercus with l/w ratio: ~ 1.9	***Sphyracephalabeccarii***
8	Dark apical wing spot in cell r4+5 proximally extending to halfway the cell (Fig. 138); central crossband quite narrow, in cell r4+5 ~ 1/7 of cell length; apical wing spot marginally linked to central wing band along veins M1 and R4+5; very pale preapical wing band almost uninterrupted and striking; dark apex of fore femur on dorsal side extending to ~ 25% of femur length	***Sphyracephaladetrahens***
–	Dark apical wing spot in cell r4+5 proximally extending to almost crossvein dm-m (Fig. 147); central crossband quite broad, in cell r4+5 ~ 1/4 of cell length apical wing spot broadly linked to central wing band along veins M1 and R4+5; pale preapical wing band interrupted giving three pale spots with a vague central one in cell r4+5; dark apex of fore femur on dorsal side extending to ~ 40% of femur length	***Sphyracephalabipunctipennis***

### ﻿ Sphyracephala babadjanidesi

Taxon classificationAnimaliaDipteraDiopsidae

Zaitzev, 1919

E96BD850-2F0B-54A7-8E2E-351AA8552A35

Figs 5 , 6, 7 , 8–11 , 12, 13 , 14–17 , 18–23 , 24–28 , 29–31 , 32 , 105 , 106 , 107 , 108 , 109 , 110–112 , Tables 1 , 2 , 3 , 4

Sphyracephala babadjanidesi Zaitzev, 1919: 3 (in Russian), 5 (in English), fig. 1. Hennig 1941a: 60, 1941b: 6, fig. 6 (repetition of Zaitzev’s description); Steyskal 1972: 13; Feijen 1989: 67; Papp et al. 1997: 137; Hilger 2000: 340; Simova-Tošić and Stojanović 2000: 149; Nartshuk 2003: 179, pl. 44, fig. 13, 2017: 128.
Sphyracephala europaea Papp & Földvári, 1997: 138, figs 1–13. Simova-Tošić and Stojanović 2000: 149, figs 1–5, table 1 (as S.europea [sic]); Hilger 2000: 338, figs 7.1, 7.2; Földvári and Meier 2002: 71; Carr et al. 2006a: 5, figs 1h, 2; Oosterbroek 2006: 130, fig. 496; Carr 2008: 114, fig. 1, table 1; Kotrba 2014: 98, fig. on p. 99; Nartshuk 2017: 129; KMNP 2018: 10^th^ p. (unpaginated); Kutsarov and Hubenov 2019: 145, figs 1–3); Jackson 2019: 61, suppl. fig. 1, 2; Turista Magazin 2023: unpag., fig. 4/6. Syn. nov.

#### Link.

https://www.flickr.com/search/?text=sphyracephala%20europaea.

#### Type series.

*Sphyracephalababadjanidesi*. Azerbaijan: 6 ***syntypes*** (♂ and ♀), Elizavetpol [later Ganja, then Kirovabad, now Ganja], vi.1916, vi.1917; type series lost according to Nartshuk (2017), who designated a ***neotype***, ♂, Azerbaijan, окр. Ганжи, р. Качкарка [okr. Ganzhi, r. Kachkarka, 40°40'12"N, 46°16'33"E], 2.vii.1933, Lukyanovich (ZIN). Type location and the nearby neotype location are well into the Asian part of Azerbaijan.

**Figure 5.**
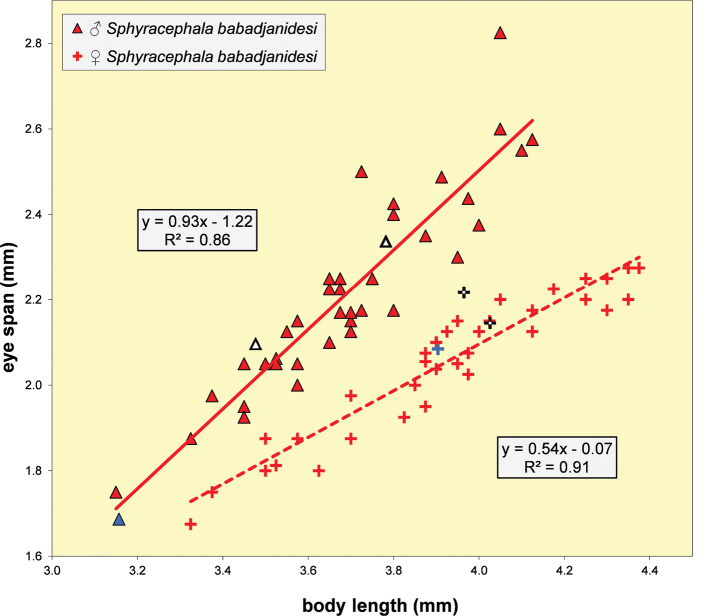
*Sphyracephalababadjanidesi*, eye span plotted against body length. Most data points were supplied by A. Stojanović (NHMBEO). These Serbian data were also used by Simova-Tošić and Stojanović (2000). Data points for two Azerbaijan flies are marked in blue, while data points of four Hungarian flies are marked in black and white.

*Sphyracephalaeuropaea*. Hungary: ***holotype***, ♂, Szeged, Maros-torok, magaspart [~ 46°14'24"N, 20°14'14"E ~ 100 m], 26.iv.1997. ***Paratypes***, 10 ♂, 7 ♀, same locality and date; 1 ♀, same locality, 16.x.1996 (all in HNHM).

#### Material examined.

Azerbaijan: 1 ♀, 1 ♂, Болчалы ЮЗ Гянджи, Азербайджан, Лукъянович 17.vii.1933, *Sphyracephalababadjanides* [sic], det. Nartshuk (RMNH) [Bolchaly (= Balchili), sw Ganja, Lukyanovich, 40°40'12"N, 46°16'33"E, 17.vii.1933, ~ 500 m]. Azerbaijan is mainly located in West Asia, but a small part (5½ districts) in the North is part of Europe as the Caucasus form the division between Eastern Europe and Western Asia. From 1920 to 1991, Azerbaijan was part of the Soviet Union (USSR). Fyodor K. Lukyanovich (1904–1942) was a Russian entomologist. Hungary: ***paratypes*** of *S.europaea*, 2 ♀, 2 ♂, Szeged, Maros-Torok, magaspart, 26.iv.1997, Paulovics and Földvári (HNHM); Georgia: 1 ?, Kakheti, Signagi, Vakiri, 41°38'43.368"N, 45°55'27.3"E [390 m], 17.vii.2024, S. Kiladze (photographs, see https://www.inaturalist.org/observations/229960704).

#### Diagnosis.

*Sphyracephalababadjanidesi* can be recognised by the following set of characters: head mainly blackish brown, face and anterior edge of frons brown; thorax and abdomen blackish; clothed in sparse, small white setulae; eye stalk very stout (~ 0.94–1.02× the widest sagittal eye diameter); very small eye span (~ 1.7–2.2 mm) in ♀ and ♂ (respectively ~ 52% and ~ 60% of body length); very low rate of dimorphism D = 0.39; rectangular basiliform prosternum; apical seta/scutellar spine ratio: 5.4–5.5; scutellar spine/scutellum ratio: 0.43 in ♀ and 0.41 in ♂; very small, scutellar spines whitish but darker basally ~ 0.13 mm; wing almost transparent with brown central and apical spots; fore femur brown with apical third blackish brown, inner side centrally with dark brown diagonal transverse band, strongly incrassate (l/w ratio: 2.7–2.8), two rows of pale slender spinous setae, inner row with ~ 4.5 setae and outer row with ~ 4.0 hardly spinous setae; tergite 1 with distinct subcircular groove; intersternite 1-2 a solid, straight, rod-like sclerite, laterally linked to sternite 2; ♀ tergite 7 consisting of two anteriorly located, triangular sclerites; ♀ sternite 7 with anteriorly two subtriangular plates, posteriorly connected to two subrectangular plates; ♀ sternite 8 two large elongate sclerites; ♀ cerci rather elongate, l/w ratio: ~ 3.2, sharply tapering apically, remarkably curled upward; small sclerotised ring present; surstyli articulate, medially directed, subrectangular with slightly concave apical side, without microtrichia, clothed in setulae, diagonal ridge on basal half of inner side. *Sphyracephalababadjanidesi* belongs to the *S.brevicornis* species group and comes closest to *S.munroi*.

#### Redescription.

The following redescription considers the original descriptions by Zaitzev (1919) and Papp et al. (1997), and especially also the description and illustrations by Simova-Tošić and Stojanović (2000). Philipp Adamovich Zaitzev (1877–1957) was the founder of research on the insect fauna of the Caucasus.

***Measurements***. Zaitzev (1919) studied six specimens and gave as length of body 3.7–4.2 mm, eye span 2.2–2.5 mm and wingspan 7–8 mm. Papp et al. (1997) gave as length of body 3.48 mm (holotype ♂), 3.10–3.50 mm (paratype ♂♂), 3.38–4.05 mm (paratype ♀♀); as wing length 3.13 mm (holotype), 2.75–3.20 mm (paratype ♂♂), 3.05–3.65 mm (paratype ♀♀); and as eye span 2.20 mm holotype), 1.90–2.20 mm (paratype ♂♂) and 1.70–2.15 mm (paratype ♀♀). The best series of measurements were given in Simova-Tošić and Stojanović (2000: table 1). However, measurements were given in μm and not, as stated, in mm, so values given must be divided by 1000 to get mm). Their most important measurements are body length ♀ 3.92 mm ± SE 0.05 (range 3.33–4.38, *n* = 35), ♂ 3.69 mm ± 0.04 (range 3.15–4.13, *n* = 38), eye span ♀ 2.05 mm ± 0.03 (range 1.68–2.28, *n* = 35), ♂ 2.21 mm ± 0.04 (range 1.75–2.83, *n* = 38). We measured the two flies from Azerbaijan and four paratypes from Hungary. For comparison, relevant measurements are summarised in Table 1. All data points for body length and eye span are plotted in Fig. 5. The actual Azerbaijan measurements for the ♀ fit well with the measurements of Zaitzev, but the ♂ is clearly at the lower end of the size range (Fig. 5). In general, measurements for the three countries are well in agreement with each other (Table 1).

**Table 1.** Measurements (mm) and ratios for *S.babadjanidesi* from Azerbaijan, Hungary (paratypes of *S.europaea*) and Serbia. The ♂ from Azerbaijan was very small. The Serbian measurements were supplied by Stojanović (pers. comm. 2024).

*Sphyracephalababadjanidesi*	Azerbaijan		Hungary		Serbia	
	♀	♂	♀	♂	♀	♂
*n*	1	1	2	2	35	38
Length of body	3.90	3.16	4.00 ± 0.03	3.63 ± 0.15	3.92 ± 0.05	3.69 ± 0.04
Eye span	2.08	1.69	2.18 ± 0.04	2.22 ± 0.12	2.05 ± 0.03	2.21 ± 0.04
Span/body ratio	0.53	0.53*	0.55 ± 0.01	0.61 ± 0.01	0.52 ± 0.00	0.60 ± 0.01
Sc. sp./scutellum	0.48	0.47	0.47 ± 0.01	0.41 ± 0.01	0.42 ± 0.01	0.40 ± 0.01
Apical seta/sc. sp.	5.00	4.89	4.83 ± 0.17	5.30 ± 0.10	5.35 ± 0.11	5.53 ± 0.10
Length of wing	3.54	2.87	3.66 ± 0.00	3.29 ± 0.00	3.43 ± 0.04	3.20 ± 0.03
Fore femur, l/w ratio	2.69	2.82	2.77 ± 0.03	2.82 ± 0.03	–	–

* Eye span/body measures by Zaitzev 0.59 and 0.60 (unsexed, but can be assumed to be males).

**Figures 6, 7.**
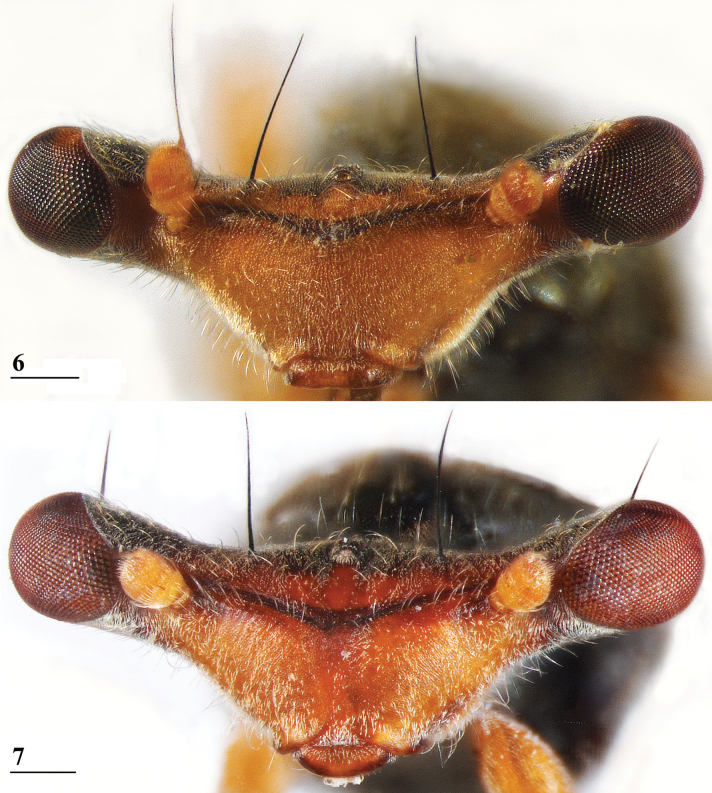
*Sphyracephalababadjanidesi*, head, anterior view **6** ♀, sw Ganja, Azerbaijan **7** ♀, paratype *S.europaea*, Szeged, Hungary. Scale bars: 0.2 mm.

**Figures 8–11.**
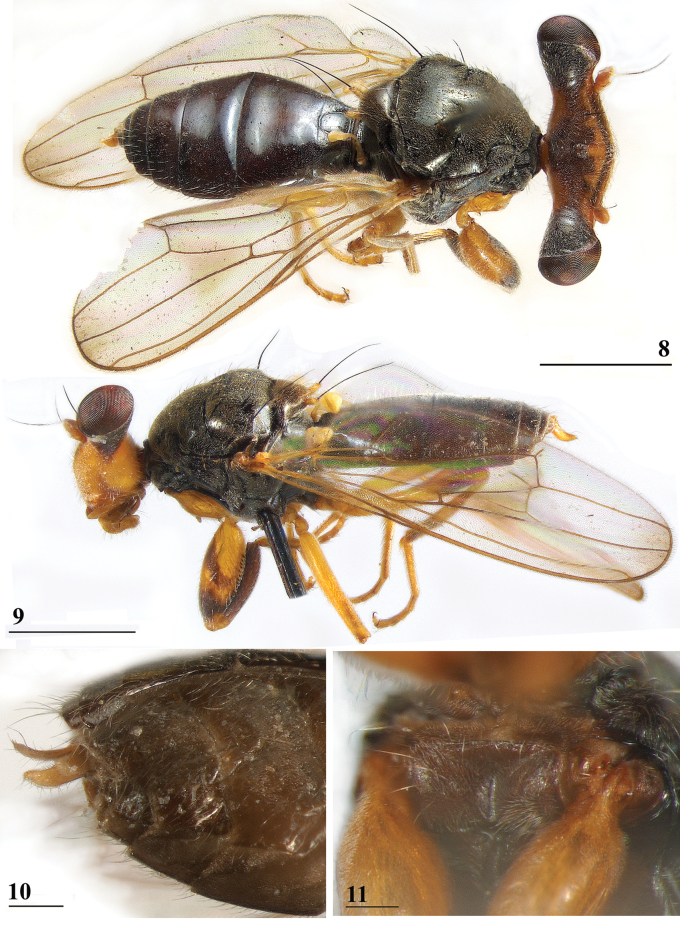
*Sphyracephalababadjanidesi***8**, **9**, **11** ♀, sw Ganja, Azerbaijan **8** habitus, dorsal view **9** habitus, lateral view **10** ♀, paratype *S.europaea*, abdominal apex with curved cerci, ventral view **11** basiliform prosternum. Scale bars: 1.0 mm (**8, 9**); 0.2 mm (**10**); 0.1 mm (**11**).

***Head***. Face and anterior edge of frons brown (Figs 6, 7, showing head of flies from Azerbaijan and Hungary); arcuate groove blackish brown; remainder of frons and stalks blackish brown, occiput brown but blackish medially (Figs 8, 9); head uniformly pruinose (Figs 6–9), clothed in sparse white setulae; frons flat; face flat, no facial teeth, lateroventral corners rounded, facial sulcus absent, but ventral facial edges slightly turned upward medially; eye stalk very stout, ~ 0.94–1.02× the widest sagittal eye diameter; eye span very small in both female (52.3% ± SE 0.2% of body length, *n* = 38) and male (59.7% ± SE 0.5% of body length, *n* = 41); a dimorphic species, rate of dimorphism very low D = 0.39 (Fig. 5, 105, 106, Table 2) [Carr et al. 2006a also indicated this species as mildly dimorphic]; inner vertical seta long, ~ 0.50 mm, 1.3× diameter of eye stalk; outer vertical seta long, ~ 0.35 mm, 0.9× diameter of eye stalk (Figs 6, 7). For additional figures of the head refer to Zaitzev (1919: fig. 1a), Papp et al. (1997: figs 1–3) and Simova-Tošić and Stojanović (2000: figs 1, 2b, 2c (antennae), 4a, 4b, 4c).

**Table 2.** Quantitative characters for *Sphyracephala* species: ratio eye span/ body length and allometric line for eye span on body length, length/width ratio of fore femur. Species are arranged in species groups and subtaxa.

*Sphyracephala*		span/body	allometric line		*n*	fore femur		*n*
		ratio	slope	dimorphism		l/w ratio	range	
*S.brevicornis*	♀	0.43 ± 0.00	0.30 ± 0.01	0.04	203	2.67 ± 0.02	2.62–2.72	6
	♂	0.44 ± 0.00	0.34 ± 0.02		131	2.65 ± 0.06	2.59–2.71	2
*S.subbifasciata*	♀	0.41 ± 0.00	0.27 ± 0.04	0.01	32	2.43	–	1
	♂	0.43 ± 0.00	0.28 ± 0.07		16	2.43	–	1
*S.nigrimana*	♀	0.38			1	2.86	–	1
	♂	0.40			1	2.73	–	1
*S.babadjanidesi*	♀	0.52 ± 0.03	0.54 ± 0.03	0.39	38	2.74 ± 0.03	2.69–2.80	3
	♂	0.60 ± 0.05	0.93 ± 0.06		41	2.82 ± 0.02	2.79–2.85	3
*S.munroi*	♀	0.61 ± 0.00	0.74 ± 0.03	0.33	40	3.63 ± 0.06	3.36–3.90	10
	♂	0.67 ± 0.00	1.07 ± 0.06		40	3.63 ± 0.03	3.50–3.83	10
*S.hearseiana*	♀	0.51 ± 0.00	0.51 ± 0.02	0.05	15	2.44 ± 0.02	2.30–2.55	15
	♂	0.53 ± 0.00	0.56 ± 0.03		15	2.48 ± 0.02	2.37–2.60	15
*S.beccarii*	♀	0.49 ± 0.00	0.49 ± 0.02	0.07	40	2.48 ± 0.02	2.40–2.61	10
	♂	0.53 ± 0.00	0.56 ± 0.03		40	2.53 ± 0.03	2.42–2.80	15
*S.detrahens*	♂	0.63			1	3.06	–	1
*S.bipunctipennis*	♀	0.49 ± 0.01	0.58 ± 0.15		4	3.46 ± 0.03	3.43–3.54	4
	♂	0.62			1	3.49 ± 0.13	3.36–3.62	2
S.nrdetrahens, Solomon Islands	♀	0.73 ± 0.01	0.97 ± 0.07	0.67	14	3.11 ± 0.06	2.81–3.36	9
	♂	0.78 ± 0.01	1.63 ± 0.07		20	3.11 ± 0.03	2.96–3.23	10
S.nrdetrahens, Japan	♀	0.73 ± 0.01	1.03 ± 0.06		6	3.19 ± 0.01	3.15–3.20	6
	♂	0.78 ± 0.02	1.40 ± 0.11		4	3.25 ± 0.02	3.20–3.29	4

***Thorax***. Collar and scutum uniformly black, pruinose (Figs 6, 7, 31), scutellum slightly more blackish brown, pruinose; scutellar spines whitish but darker basally, densely pruinose (Figs 8, 9); scutum and scutellum with sparse setulae; pleura dark black, largely pollinose, only katepisternum and ventral section of anepimeron glossy (Fig. 9); posterior notopleural seta long, infra-alar seta very long (Fig. 8), infra-alar seta more than twice longer than posterior notopleural seta (Simova-Tošić and Stojanović (2000) give for the notopleural seta a length of ~ 0.29 mm and for the infra-alar seta a length of ~ 0.67 mm); supra-alar carina just visible; basiliform prosternum large, rectangular, with deep medial groove, prosternum laterally close to propleuron but distinct; scutal length/scutal width ratio: ~ 0.9; scutellum trapezoid; scutellar spines very small, straight, slightly turned upward, diverging at angle of ~ 50°; scutellar spine/scutellum ratio: 0.43 in ♀ and 0.41 in ♂ (Table 3); scutellar spine/length of body ratio: 0.055 in ♀ and 0.051 in ♂; apical seta/scutellar spine ratio: 5.4 in ♀ and 5.5 in ♂; scutellar length/scutellar width (at base) ratio: 0.57 in ♀ and in ♂. For additional figures of the thorax can be referred to Simova-Tošić and Stojanović (2000: figs 3e, 3f, scutellum and postscutellum).

**Table 3.** Quantitative characters for *Sphyracephala* species: ratio scutellar spine/scutellar length and ratio apical seta/ scutellar spine. Species are arranged in species groups and subtaxa.

*Sphyracephala*		scutellar spine/scutellum	*n*	apical seta/scutellar spine	*n*
		ratio		ratio	
*S.brevicornis*	♀	0.65 ± 0.03	6	2.82 ± 0.06	6
	♂	0.64 ± 0.00	2	3.00	1
*S.subbifasciata*	♀	0.54	1	3.86	1
	♂	0.54	1	3.86	1
*S.nigrimana*	♀	0.75	1	3.22	1
	♂	0.65	1	2.82	1
*S.babadjanidesi*	♀	0.43 ± 0.01	28	5.36 ± 0.13	20
	♂	0.41 ± 0.01	40	5.50 ± 0.09	35
*S.munroi*	♀	0.43 ± 0.01	10	6.10 ± 0.22	10
	♂	0.44 ± 0.01	10	6.11 ± 0.11	9
*S.hearseiana*	♀	0.56 ± 0.01	12	3.60 ± 0.09	9
	♂	0.55 ± 0.01	10	3.71 ± 0.12	7
*S.beccarii*	♀	0.48 ± 0.01	15	3.91 ± 0.10	15
	♂	0.53 ± 0.01	10	3.85 ± 0.11	10
*S.detrahens*	♀	0.88	1	2.93	1
	♂	1.10 ± 0.26	2*	2.64	1
*S.bipunctipennis*	♀	1.02 ± 0.02	4	2.50 ± 0.02	4
	♂	1.12	1		
S.nrdetrahens Solomon Islands	♀	0.98 ± 0.02	10	2.85 ± 0.10	6
	♂	0.97 ± 0.02	10	2.94 ± 0.10	6
S.nrdetrahens Japan	♀	0.90 ± 0.01	6	2.38 ± 0.08	2
	♂	0.85 ± 0.04	4	2.86 ± 0.14	4

* Both from Sulawesi, but perhaps not conspecific.

**Figures 12, 13.**
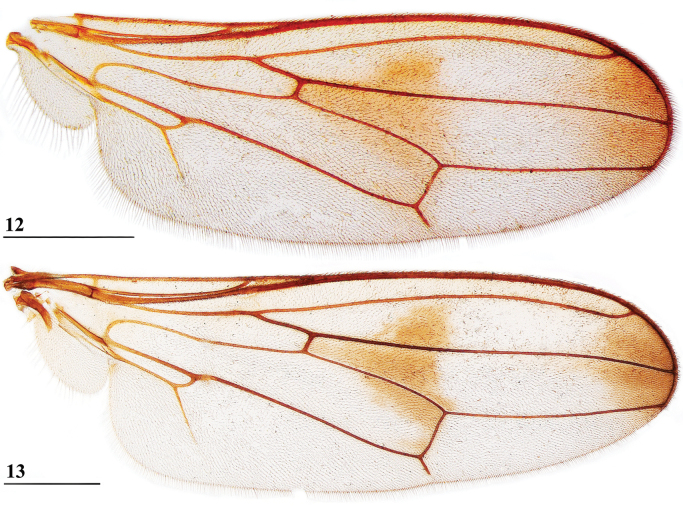
*Sphyracephalababadjanidesi*, wing **12** ♂, sw Ganja, Azerbaijan **13** ♀, paratype *S.europaea*, Szeged, Hungary. Scale bars: 0.5 mm.

***Wing***. Almost transparent with brown central and apical spots (Figs 12, 13 showing wing of flies from Azerbaijan and Hungary); apex with rounded spot in cells r2+3 and r4+5 just extending in cells r1 and m1; central irregular spot running from posterior end of crossvein dm-m to almost vein R2+3, section in cell r2+3 rounded, section in cell r4+5 running from crossvein r-m to crossvein dm-m, section in cell bm+dm in anterodistal corner and along crossvein dm-m; vein CuA+CuP from vein CuP onward extending under angle of 45° to just past halfway wing margin in straight line; vein M4 continuing distal of crossvein dm-m to almost three-quarters the wing margin; cell cua slightly broadening distally, apically rounded (Figs 12, 13); crossvein h distinct; glabrous area only includes tiny basal spot in cell br.

**Figures 14–17.**
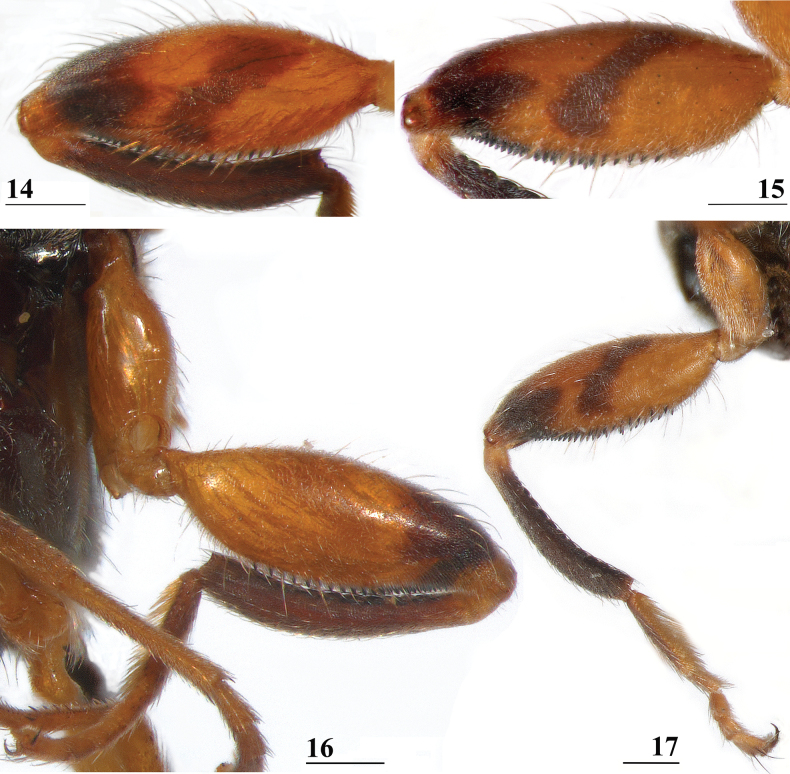
*Sphyracephalababadjanidesi***14, 15** fore femur, inner side **16, 17** fore leg **14** ♀, sw Ganja, Azerbaijan **15** ♀, paratype *S.europaea*, Szeged, Hungary **16** ♀, outer side, sw Ganja, Azerbaijan **17** ♂, inner side, paratype *S.europaea*, Szeged, Hungary. Scale bars: 0.2 mm.

***Legs*.** Fore coxa and trochanter brown, especially anteriorly pruinose, clothed in some white setulae; fore femur (Figs 14, 15 showing femora of flies from Azerbaijan and Hungary) brown, apical third blackish brown on inner and outer side, inner side centrally with dark brown diagonal transverse band, thinly pruinose, sparsely clothed in small setulae; fore tibia dark brown, thinly pruinose; basitarsus dark brown, other tarsomeres brown (Figs 16, 17), thinly pruinose and with rows of blackish setulae; mid and hind legs brown, femora with darker brown apices, hind tibia with darker brown apex; fore femur strongly incrassate (Table 2), l/w ratio in Azerbaijan ♀ 2.69 and in Azerbaijan ♂ 2.82, l/w ratio in two *S.europaea* paratype ♀ from Hungary 2.77 ± 0.03 (range 2.73–2.80) and in two *S.europaea* paratype ♂ 2.82 ± 0.03 (range 2.79–2.85) [Papp et al. (1997) stated that the fore femur in *S.europaea* is definitely thicker than in *S.babadjanidesi* and a major differential character. Basing themselves on Zaitzev’s (1919) drawing, they gave a ratio width/length for *S.babadjanidesi* of 27/75 (i.e., l/w ratio: 2.78). According to Papp et al., the ratio length/width in the holotype of *S.europaea* came to 2.41, while in the paratype females it was 2.44. However, the data at our disposal clearly show no difference in the ratio l/w between *S.europaea* and *S.babadjanidesi*.]; fore femur with two rows of pale spinous setae on distal two-thirds (Figs 14, 15), especially the setae on the outer site are very slender and hardly qualify as “spinous”, in total 8.5 ± SE 0.2 setae (*n* = 10, range 8–9, ♀ and ♂ combined), inner row with 4.5 ± 0.2 (*n* = 10, range 4–5) setae and outer row with 4.0 ± 0.0 setae (*n* = 11, range 4); two rows of tubercles on distal three-quarters with in total 50.2 ± 0.4 tubercles (*n* = 10, range 48–52, ♀ and ♂ combined), inner row with 24.4 ± 0.3 (*n* = 10, range 23–26) tubercles and outer row with 25.5 ± 0.4 (*n* = 11, range 23–27) tubercles. Simova-Tošić and Stojanović (2000: fig. 3a) illustrated setae and tubercles on the fore femur.

**Figures 18–23.**
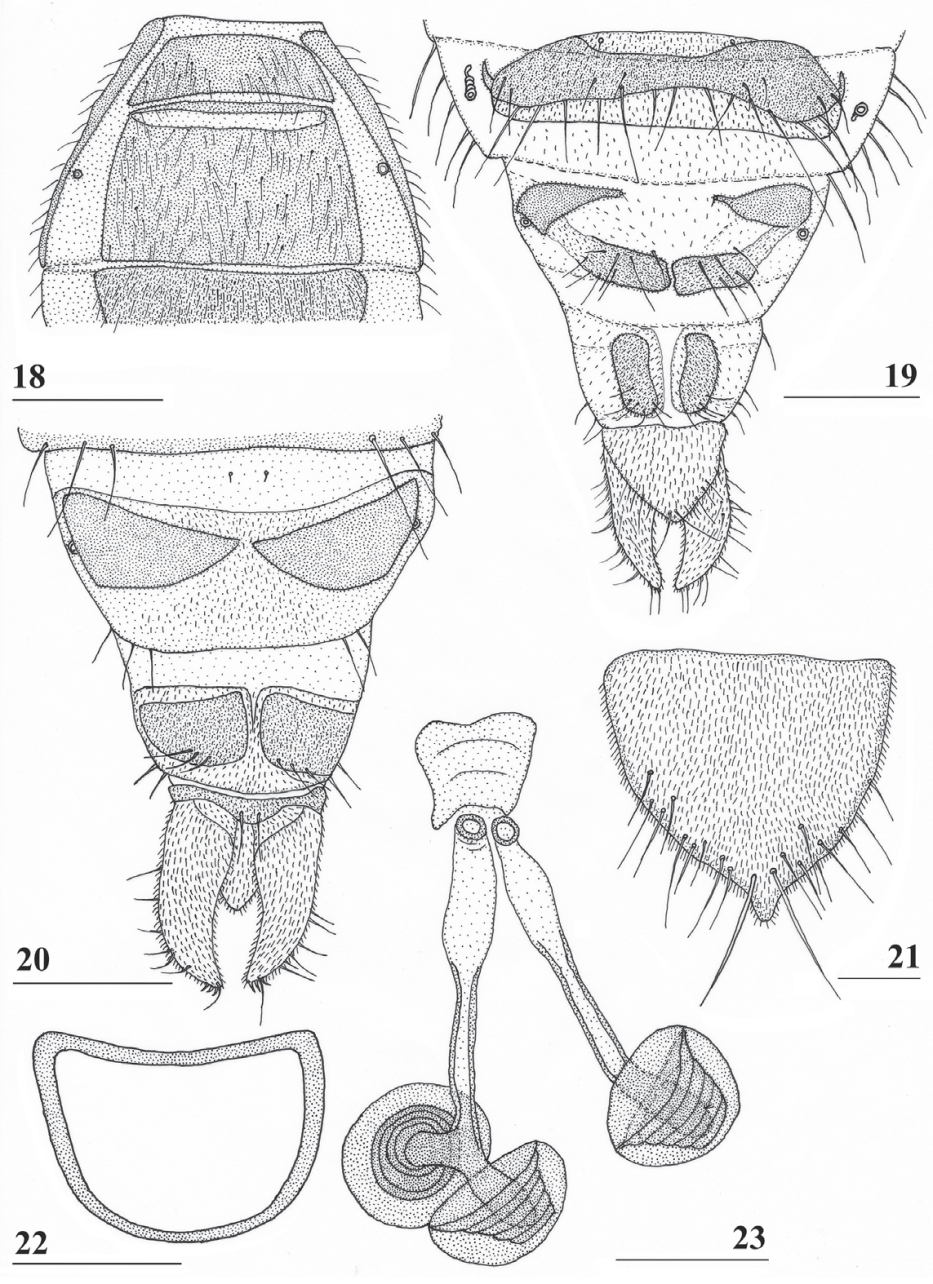
*Sphyracephalababadjanidesi*, ♀, sw Ganja, Azerbaijan **18** sternite 1, intersternite 1-2 and sternite 2, ventral view **19** postabdomen, ventral view **20** postabdomen, dorsal view **21** subanal plate, ventral view **22** sclerotised ring **23** spermathecae. Scale bars: 0.5 mm (**18**); 0.2 mm (**19, 20**); 0.05 mm (**21–23**).

***Preabdomen***. Tergites (Fig. 8) blackish brown, thinly pruinose, small white setulae especially laterally; tergite 1 with very distinct subcircular groove and vague transverse ridges (Fig. 8, groove also shown in Zaitzev (1919: fig. 1), Papp et al. (1997: fig. 1), and Simova-Tošić and Stojanović (2000: fig. 4d, e); suture between tergites 1 and 2 very distinct; sternites 1–6 dark brown; sternites 1 and 2 glossy, clothed in small setulae (Fig. 18); other sternites thinly pruinose, sparsely clothed in small white setulae; sternite 1 short, trapezoid; intersternite 1-2 a solid straight rod-like sclerite, laterally linked to sternite 2 (Fig. 18); sternite 2 a slightly trapezoid plate; sternites 3–5 rectangular plates; sternite 6 (Fig. 19) somewhat curved, strongly sclerotised, anteromesally a non-sclerotised section.

***Female postabdomen***. Postabdomen (Fig. 19) with “normal” shape, not long and narrow like *S.munroi*; tergite 7 represented by two glossy, well sclerotised, anteriorly located, triangular sclerites, just separated mesally (Fig. 20); tergite 8 two subrectangular, thinly pruinose, sclerites, separated on the meson; tergum 10 short, on the meson broader and posteriorly rounded, thinly pruinose, one pair of apical setulae; cerci curled upward, a striking feature clearly visible in both females from Hungary and Azerbaijan (Figs 9, 10; see also Simova-Tošić and Stojanović 2000: fig. 4i), rather elongate, l/w ratio: ~ 3.2, sharply tapering apically, clothed in microtrichia and setulae, apically 3 tiny spine-like setulae (Fig. 20); sternite 7 (Fig. 19; see also Papp et al. 1997: fig. 10) with anteriorly two subtriangular, almost bare, plates well separated on the meson, posteriorly these plates are connected to two posterior subrectangular plates just separated on the meson, these latter plates pruinose and clothed in some setulae; spiracle 7 in membrane; sternite 8 represented by two elongate sclerites, well separated on the meson (Fig. 19), pruinose and posteriorly with some setulae; subanal plate (Fig. 21) triangular to mitre-shaped with apically a tiny extension, pruinose, apex with one pair of longer setulae, ~ 9 pairs of setulae posteriorly; spermathecae (Fig. 23) mushroom-shaped with medium-sized, bell-shaped, hollow, more sclerotised, striated, inner structure, no protuberances, spermathecal ducts very short and broadening distally; sclerotised ring of ventral vagina (Fig. 22) small, anteriorly straight and posteriorly semi-circular, arms very slender.

**Figures 24–28.**
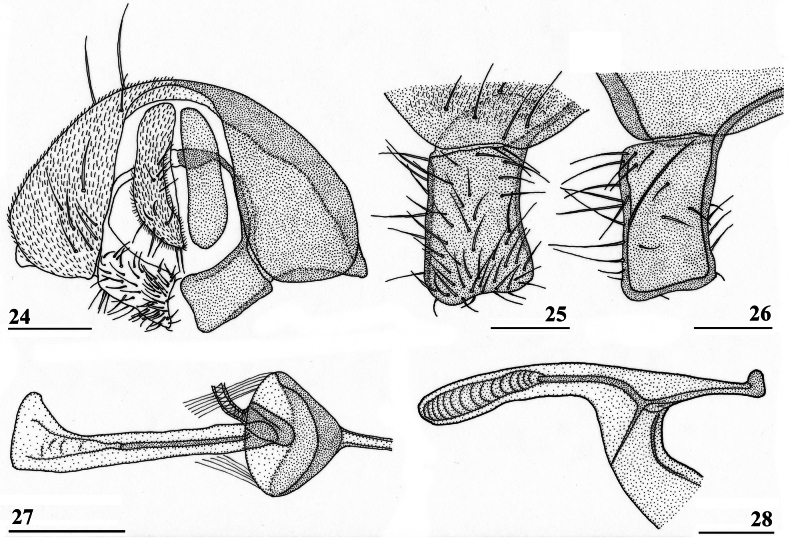
*Sphyracephalababadjanidesi*, ♂, sw Ganja, Azerbaijan **24** epandrium, cerci, surstyli, posterior view **25** surstylus, outer view **26** surstylus, inner view **27** ejaculatory apodeme + sac **28** phallapodeme. Scale bars: 0.1 mm (**24, 27, 28**); 0.05 mm (**25, 26**).

***Male postabdomen***. Syntergosternite 7+8 very slender and wide, weakly sclerotised; spiracles 7 in membrane just anteriorly of syntergosternite; epandrium (Fig. 24) rounded, with a large mesal gap, clothed in microtrichia and ~ 10 pairs of setulae; surstyli articulate, l/w ratio: ~ 1.5–1.7, almost touching on the meson, (Figs 24–26), simple, subrectangular with slightly concave apical side, outer side (Fig. 25) without microtrichia, clothed in ~ 50 setulae, inner side (Fig. 26) with only 20 setulae and an almost diagonal ridge on basal half; surstyli interconnected via slender processus longi, processus broadening medially (Fig. 24); cerci slender, rather elongate, l/w ratio: 3.9, clothed in microtrichia and on apical half with ~ 10 setulae; phallapodeme (Fig. 28) with slender anterior arm, corners rounded, anterior arm 1.5× longer than posterior arm, lateral processes broad; ejaculatory apodeme straight, slender but apically widening to twice its width (Fig. 27), ejaculatory sac normal-sized. Papp et al. (1997: figs 4, 5, 8) give lateral views of the male genital complex, also illustrating the phallic complex with short male genital process. Simova-Tošić and Stojanović (2000: fig. 5a, b) likewise illustrate the lateral view of the male genital complex.

#### Biology.

**Figures 29–31.**
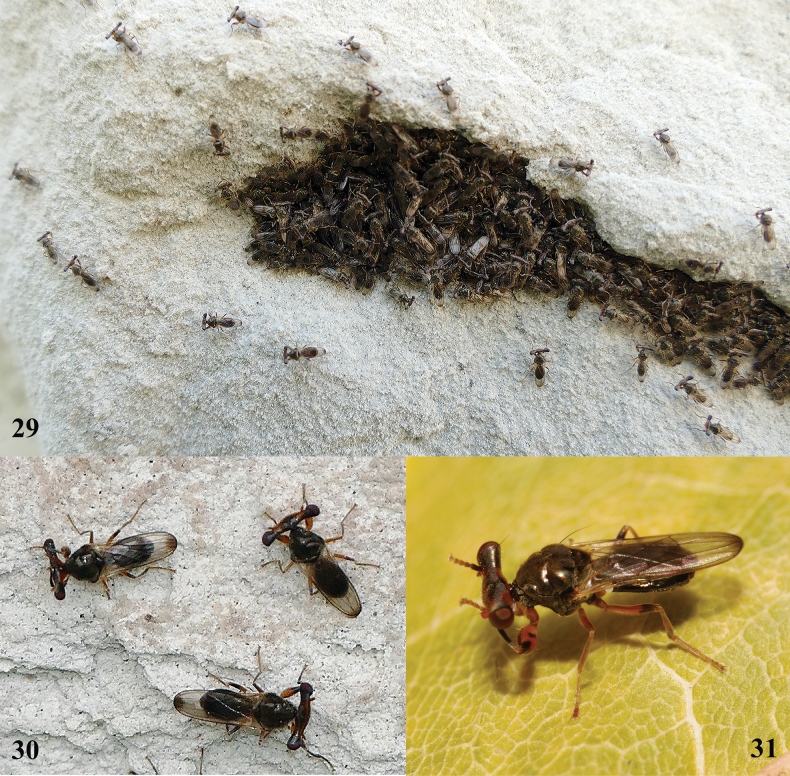
*Sphyracephalababadjanidesi*, live **29** cluster of flies in cleft, Nikopol, Danube River, Bulgaria (photograph © Yordan Kutsarov) **30** three flies, same data as for 29 **31** fly showing fore femur, Szeged. Maros River, Hungary (photograph © Walter Pflieger).

Zaitzev (1919) reported that *S.babadjanidesi* was attracted by electric light in the evening. Papp et al. (1997) reported on hundreds of *S.europaea* in October on an overwintering site. Returning in April, the flies were there again, though in smaller numbers. Paulovics (1998) presented data on summer occurrences and distribution along the Hungarian Maros river as well as observations on its ethology. The fly preferred plain and sandy parts of the riverbank and avoided the zone covered by mud. Flies were found 0.5–1.5 m from the river edge. On or near a dead frog, seven or eight *Sphyracephala* were found. A single fly was also found feeding on a dead ant. In a two-week period in October, the number of assembling flies increased from several to ~ 500. Simova-Tošić and Stojanović (2000) found a cluster of a few thousand flies of *S.europaea* in early November. Males and females occurred in approximately equal numbers. Kutsarov and Hubenov (2019) reported on clusters of thousands of flies in Bulgaria (see Figs 29, 30). Nartshuk (2017) noted the gregarious behaviour of *S.babadjanidesi* in Azerbaijan. She also noted that there were only small differences in the descriptions for *S.babadjanidesi* and *S.europaea* and that the study of type material would be necessary to determine their synonymy. Papp et al. (1997) and Simova-Tošić and Stojanović (2000) reported on 44 ♀♀ and 55 ♂♂ which would give a sex ratio of 1 ♀:1.25 ♂ (Table 4). However, the latter authors stated that ♂♂ and ♀♀ occurred in equal numbers.

**Table 4.** Sex ratios in *Sphyracephala* species. Species are arranged in species groups and subtaxa.

*Sphyracephala*	*n* ♀	*n* ♂	Ʃ *n*	♀ : ♂	Source
*S.brevicornis*	185	119	304	1.00:0.64	Feijen 1989
*S.subbifasciata*	56	31	87	1.00:0.55	Feijen 1989
*S.babadjanidesi* (as *europaea*)	44	55	99	1.00:1.25 1:1*	Papp et al. 1997 Simova-Tošić and Stojanović 2000
*S.munroi*	318	307	625	1.00:0.97	FBUB, NHRS, RMNH
*S.hearseiana*	17	17	34	1.00:1.00 1:1**	RMNH Sen 1921
*S.beccarii*, Afrotropical Continent	622	635	1257	1.00:1.02	RMNH
*S.beccarii*, Madagascar	547	285	832	1.00:0.52	CAS, NMSA, CSCA, RMNH
*S.bipunctipennis*	11	4	15		NHMUK, TAU, RMNH
*S.detrahens*, Sulawesi	14	4	18		NHMUK, RMNH
S.nrdetrahens, Japan	247	376	623	1.00:1.52	Ôhara 1993, 1997, RMNH
S.nrdetrahens, Solomon Islands	15	29	44	1.00:1.93	RMNH
S.nrdetrahens, Papua N. G.	1	6	7		RMNH

* Simova-Tošić and Stojanović (2000): “The swarm contained few thousands of males and females approximately in equal number.” ** Sen (1921): “The mass consisted of flies of both sexes, in approximately equal numbers.”

Laboulbeniales (Ascomycota) have never been found on *S.babadjanidesi*, *S.nigrimana*, and the two Nearctic *Sphyracephala*. The long hibernation period might form the reason for this. Rossi and Feijen (2018) noted that Laboulbeniales are common on African Diopsidae, but considerably less common on Oriental Diopsidae. In Afrotropical *Sphyracephala*Laboulbeniales are very common, but in Oriental *Sphyracephala* they are rare. It should be noted that the first fossil record of the order Laboulbeniales was found on a fossil diopsid *Prosphyracephala* in Baltic Amber (Rossi et al. 2005). Grace and Carr (2020) recorded for *S.babadjanidesi* (as *europaea*) 4 subfamilies of mariner transposons against none in *S.beccarii*.

#### Distribution.

**Figure 32.**
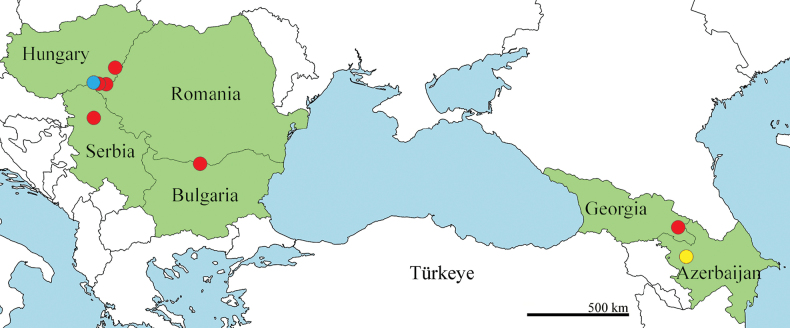
Distribution map for records of *Sphyracephalababadjanidesi* (red dots). The yellow dot indicates the type locality of *S.babadjanidesi*, while the blue dot indicates the one for *S.europaea*.

The original type series for *S.babadjanidesi* (Zaitzev 1919) and its neotype (Nartshuk 2017) all originate from the Ganja Region in the Asian part of Azerbaijan. The neotype forms part of a large collection (ZIN) of more than 500 specimens originating from almost the same place as the type series (Nartshuk 2017). In 2024, *S.babadjanidesi* was photographed in Georgia (https://www.inaturalist.org/observations/229960704). The type series for *S.europaea* came from the Maros River in Hungary. This river is a tributary of the Tisza River which in its turn is the main tributary of the Danube. According to Paulovics (1998), the species exists all along the Hungarian part of the Maros (Fig. 31). The species was later (KMNP 2018) recorded from the Körös-Maros Nemzeti Park in Hungary, near the border with Romania (~ 46°41'23"N, 21°10'28"E, ~ 80 m). Rivers in this park are tributaries of the Tisza. Rahmé published pictures (https://www.flickr.com/photos/eurythyrea/5126015816) taken in Makó, Csongrád, Hungary (46°12'11"N, 20°27'11"E, 83 m). Simova-Tošić and Stojanović (2000) extensively reported on the presence of *S.europaea* in Serbia along the Danube, ca 2 km from the mouth of the Tisza at Stari Slankamen (45°9'5"N, 20°14'44"E, ~ 100 m) Stojanović (pers. comm.) observed the flies again on 14.x.2006 in the same locality. Early in the morning (8:00 am) ~ 40 specimens could be observed in the same hollow on the loess profile. Later, at around 4:00 p.m., more than 200 specimens were gathered in the same place, spread out over an area of ca 3 m^2^. Kutsarov and Hubenov (2019) recorded *S.europaea* in Bulgaria, east of the town of Nikopol, next to the rocky monastery St. Stefan (43°42'36"N, 24°54'51"E, 60 m) on the limestone rocks along the Danube River on the border with Romania. On the internet references for *S.europaea* in Romania can be found (https://www.flickr.com/photos/eurythyrea/5126015816). All locations for *S.europaea* are along the Danube River and its tributaries. Papp et al. (1997) stated “that Hennig (1941b) hypothesized the occurrence of *Sphyracephala* in South Europe including Hungary”. However, that view, also repeated in Földvári and Meier (2002), cannot be deduced from Hennig’s paper. The various collecting localities and the two type localities are shown on the map (Fig. 32). In Hungary, this fly has a nature conservation status: collecting it carries a 10,000 HUF fine (Turista Magazin 2023).

#### Remarks on synonymy.

Papp et al. (1997) described *S.europaea* and declared it to be the first known species of the family Diopsidae in Europe. However, fossil species are well known from Europe. Papp et al. (1997) considered *S.babadjanidesi* the most closely related species to *S.europaea*. By mistake, they reported Armenia as type locality of *S.babadjanidesi*. Papp and Földvári (in Papp et al. (1997)), while describing *S.europaea*, had no access to specimens of *S.babadjanidesi*. They had to rely on the description and illustrations by Zaitzev (1919), which for its time were certainly of a good standard. However, Zaitzev did not study the genitalia of *S.babadjanidesi*. Describing a closely related species, without access to flies from the type locality of *S.babadjanidesi* and without knowledge of genitalia morphology, is not a procedure to be recommended.

Papp et al. (1997) list three “features” that are different between *S.babadjanidesi* and *S.europaea*. The first one concerns the colour of the fore tarsi. According to Papp et al. “both description and figure [of Zaitzev] say that fore basitarsus and tarsomeres are yellow in *Sphyracephalababadjanidesi*, contrasting those of *S.europaea*.” In the description, Papp et al. (1997) state “Fore basitarsus all black, dorsal surface of other fore tarsomeres dark grey, at most 5^th^ tarsomere light”. Comparison of photographs (Figs 16, 17) clearly shows that the colour of basitarsus and tarsomeres are similar for flies from Azerbaijan and Hungary. The second difference they listed is “No dark hue in r1 cell of *Sphyracephalaeuropaea*, contrary to *S.babadjanidesi*.” This feature is certainly a bit overrepresented in Zaitzev (1919: fig. 1), but is not repeated in the text. Now, comparison of the wings shows no difference in this regard (Figs 12, 13). In wings from both Azerbaijan and Hungary, the very apex of cell r1 can be slightly darker. The third difference given by Papp et al. (1997) concerns the l/w ratio of the fore femur. In the section on the legs of *S.babadjanidesi*, their statement that fore femora of *S.europaea* are more incrassate, has already been rejected (see above, and Table 1). The measurements given by Papp et al. (1997) are somewhat haphazard and not well presented. Fortunately, Simova-Tošić and Stojanović (2000) later presented high quality measurements for many characters and based on large series of males and females. The graphs for the ratio’s eye span/body length for flies from Azerbaijan, Hungary, and Serbia (Fig. 5) also clearly show no differences in this regard. Likewise, study of the wing morphometrics (Figs 107–109) supports the conspecificity of *S.babadjanidesi* and *S.europaea*.

We present comparative colour photographs for flies from Azerbaijan and Hungary for anterior head (Figs 6, 7), wings (Figs 12, 13) and inner side of fore femur and whole fore legs (Figs 14–17, 31). These already give a strong indication that the same species is involved. Comparison with the large sets of genitalia drawings by Papp et al. (1997) and Simova-Tošić and Stojanović (2000) confirms the view that flies from Azerbaijan, Hungary, and Serbia are conspecific.

### ﻿ Sphyracephala beccarii

Taxon classificationAnimaliaDipteraDiopsidae

(Rondani, 1873)

4A329B7F-CE39-584D-BC57-E93F588E751E

Figs 2 , 4 , 33–36 , 37 , 38–42 , 43–47 , 48–53 , 105 , 106 , 107 , 108 , 109 , 110–112 , Tables 2 , 3 , 4

Diopsis beccarii Rondani, 1873: 289.
Hexechopsis beccarii (Rondani): Rondani 1875: 442. Osten Sacken 1882: 235; Brunetti 1907: 163; Bezzi 1922: 71; Eggers 1925: 488, 493; Séguy 1949: 74, 1955: 1123.
Sphyracephala beccarii (Rondani): Osten Sacken 1882: 235. Bezzi 1922: 69; Brunetti 1928: 273; Curran 1928: 274; Hennig 1941a: 62, 1941b: 6, figs 1, 5d, 1965: figs 46, 49c, 58a; Séguy 1949: 75, figs 6, 7; 1950: 276; Vaillant 1953: 11, figs on p. 12; Collart 1954: 329; Lindner 1954: 28; 1962; 17; Séguy 1955: 1124; Guiglia 1957: 194; Descamps 1957: 17, figs 7b–7f; van Bruggen 1961: 425, figs 12, 15; Steyskal 1972: 13; Feijen 1978: 19; 1983: fig. 5; 1987: 420; 1989: 67; Cogan and Shillito 1980: 584; Ferrar 1987: figs 28.34, 28.49, 28.62, 28.63, table 28.1; Rossi 1990: 3; Blackith and Guillet 1995: 66; Jakobs 1997: 13; McAlpine 1997: 172, figs 23, 24, 27; 2011: 150, figs 123, 125; Papp et al. 1997: 137; Hariri et al. 1998: 254, tabs 1–4; Baker 1999: tab 2-1, figs 2-2, 2-3, app. A; Lande and Wilkinson 1999: table 1; Wilkinson and Taper 1999: 1687, fig. 1, table 1; Simova-Tošić and Stojanović 2000: 149; Hilger 2000: 337, figs 7.3–7.7; Meier and Hilger 2000: 6, figs 9–11, table 2; Baker and Wilkinson 2001: figs 2, 3, table 1; Baker et al. 2001: figs 1, 2; Hurley et al. 2001: 408, 1C-E, 2D, 3C (as *beccarri*); Hurley 2002: 2, figs 1.3, 2.1.C-E, 2.2.D. 2.3.C; Meier and Baker 2002: 333, figs 1b, 2; Cotton 2004: 2, figs 4.1, 4.2, 4.3, table 4.1; Cotton et al. 2004: 1310, figs 1-3, table 1; Chapman et al. 2005: 534; Carr et al. 2005: 403, figs 4, 5, 2006a: 5, figs 1g, 2, 2006b: fig. 22; Warren and Smith 2007: figs 1a, 2, 3b, 3d; Carr 2008: 114, fig. 1, table 1; Dawah and Abdullah 2008: 89; Ribak et al. 2009; 861, figs 1, 3, 4, 5, 7, table 1; Hauser et al. 2011: 765, figs 1–3; O’Hara et al. 2011: 96; Baker et al. 2012: 2368; Marshall 2012: fig. 8 on p. 473; El-Hawagry et al. 2013: 60, 2016: 124, 2017: 17; Voje and Hansen 2013: figs 1, 2, tab 1; Baker et al. 2016: 898, figs 1, 2, table 1; Feijen et al. 2017: 76, figs 1, 5, 11, 17, 18, 20; Feijen et al. 2018: 142, figs 189, 194, 199; Mader 2017: 108; Nartshuk 2017: 129; Jackson 2019: suppl. fig. 1, 2; Picker et al. 2019: 344; Grace and Carr 2020: 18/24, table1; Feijen and Feijen 2021: 1484, figs 4, 48; 2022: 1111, fig. 9.56a; 2023: 84. [Given the frequent spelling errors in beccarii (1 or 2 c’s, 1 or 2 r’s, 1 or 2 i’s), this should be taken into account for digital searches.]
Sphyracephala africana Karsch, 1888: 380, pl. 4 fig. 11. Speiser 1910: 166, 1924: 100; Eggers 1925: 493; Brunetti 1926: 84, 1928: 273; Curran 1928: 274; Séguy 1933: 32, 1938: 237, 1949: 75, 1955: 1124; Collart 1954: 329; Lindner 1954: 28; Descamps 1957: 17; van Bruggen 1961: 426; Steyskal 1972: 13; Feijen 1978: 20; Cogan and Shillito 1980: 584; Feijen et al. 2017: 76.
Sphyracephala hearseiana (Westwood): Bezzi 1922: 69, Algeria (misidentification). Hennig1941b: 6; Séguy 1955: 1124; van Bruggen 1961: 426.

#### Type series.

Sphyracephala (Diopsis) beccarii. Eritrea: Sciotel, Bogos [1870, 15°35'N, 38°20'E, 780 m], 61 ***syntypes*** in MSNG (Sforzi and Sommaggio 2021), more ***syntypes*** in various other museums (MLUH, NHMUK). No lectotype has been nominated (Sforzi and Sommaggio 2021), although Guiglia (1957) mentioned “Tipo e numerosi cotipi”.

*Sphyracephalaafricana*. Tanzania: ***holotype***, ♀, Bondei, [~ 5°00'S, 39°00'E, 100 m, i.1886/87], ZMHB. The holotype is not listed in Rohlfien and Ewald (1970), but it is present in the ZMHB collection (Sven Marotzke, pers. comm. 2024).

#### Material examined.

It would go too far to list all the *S.beccarii* we have examined since 1971. Here we only list the totals examined per country or region: Eritrea, **syntypes** 3 ♀, 3 ♂, Sciotel, Bogos, 1870, O. Beccari (MLUH, V. Röder collection); Algeria, 1 ?sex, Rhouffi, vii.1949, Vaillant (ZSM); Arabian Peninsula 75 ♀, 80 ♂ (see Feijen et al. 2017); Benin, 1 ♀ (RMNH); Botswana, 1 ♀ (RMNH); Burkina Faso, 1 ♂ (RMNH); Cameroon, 1 ♀ (BMSA); DR Congo, 3 ♀, 5 ♂ (CSCA); Ethiopia, 2 ♀, 1 ♂ (FBUB); Gambia 1 ♀ (RMNH); Ghana, 19 ♀, 14 ♂ (RMNH, CSCA); Kenya, 4 ♀, 4 ♂ (RMNH); Madagascar, 528 ♀, 276 ♂ (CAS); Malawi, 314 ♀, 322 ♂, 1971–1975 (RMNH); Mozambique, 131 ♀, 135 ♂, 1976–1982 (RMNH); Niger, 8 ♀ (RMNH); Senegal, 2 ♀ (RMNH); South Africa 6 ♀, 1 ♂ (RMNH); Tanzania, 26 ♀, 35 ♂, 1982–1988 (RMNH); Togo, 17 ♀, 32 ♂ (FBUB, SMF, RMNH; Zambia, 1 ♀ (RMNH); Zimbabwe 7 ♀, 2 ♂ (AMGS, RMNH). In total 622 ♀ and 635 ♂ were examined for Continental Africa and the Arabian Peninsula, giving a balanced sex-ratio of 100 ♀:102 ♂. However, a different picture emerged for Madagascar: 528 ♀and 276 ♂ were found, based on 84 malaise trapping periods in 2002–2004, which gives a sex-ratio of 100 ♀:52 ♂ (see also Table 4). This striking difference will be discussed in the section on sex-ratio.

#### Diagnosis.

*Sphyracephalabeccarii* can be recognised by the following set of characters: head brown, thorax and abdomen blackish brown; sparsely covered with small setulae; frons with dark brown semicircular band; occiput yellowish brown; eye stalk stout (~ 0.75–0.80× the widest sagittal eye diameter), moderately sized for a *Sphyracephala*; very small eye span (~ 2.1 mm) in both ♀ and ♂ (respectively ~ 49% and ~ 53% of body length); monomorphic with rate of dimorphism D = 0.07; distinct precoxal bridge; apical seta/scutellar spine ratio: ~ 3.9; scutellar spine/scutellum ratio: 0.50; small, pale scutellar spines ~ 0.17 mm; transparent wings; fore femur brown with apical fifth dark brown, inner side with dark brown transverse stripe on central third, strongly incrassate, l/w ratio: 2.5–2.6, with two rows of black spinous setae, inner row with ~ 6.0 setae, outer row with ~ 1.2 setae; tergite 1 with vague transverse ridges, on the meson two parallel, longitudinal grooves; intersternite 1-2 very slender, laterally connected to main sternite 2; ♀ tergite 7 and sternite 7 divided in two small sclerites almost touching laterally; ♀ cerci broad, l/w ratio: ~ 1.9; ♀ sternite 8 represented by two small sclerites, almost touching on the meson; no sclerotised ring; surstyli articulate, almost touching on the meson, tapering apically towards an upturned apex, anterior side with microtrichia on basal third and ~ 25 setulae on apical half. *Sphyracephalabeccarii* belongs to the *S.hearseiana* species group and can be considered the sister species of *S.hearseiana*.

#### Redescription.

The following redescription considers the original descriptions by Rondani (1873) and Karsch (1888), description and figures by Hennig (1941b: figs 1, 5d; 1965: figs 46, 49c, 58a), Séguy (1949: figs 6, 7), Vaillant (1953: figs on p. 12), the table of differences between *S.beccarii* and *S.munroi* by Collart (1954), descriptions and figures by Descamps (1957: fig. 7b–f), description and illustrations by van Bruggen (1961: figs 13, 14, 16), Feijen (1983: fig. 5), description and figures of antenna by McAlpine (1997: 172, figs 23, 24, 27; 2011, figs 123, 125), figures by Hilger (2000: figs 7.3–7.7), egg description and figures by Meier and Hilger (2000: figs 9–11).

***Measurements***. Body length ♀ 4.26 mm ± SE 0.04 (range 3.54–4.64, *n* = 40), ♂ 3.91 mm ± 0.04 (range 3.32–4.27, *n* = 40), eye span ♀ 2.09 mm ± 0.02 (range 1.75–2.31, *n* = 40), ♂ 2.08 mm ± 0.02 (range 1.78–2.27, *n* = 40); wing length ♀ 3.46 mm ± 0.04 (range 3.17–3.60, *n* = 10), ♂ 3.09 mm ± 0.08 (range 2.75–3.54, *n* = 10); length of scutellar spine ♀ 0.176 ± 0.004 (range 0.169–0.193, *n* = 10), ♂ 0.171 mm ± 0.004 (range 0.145–0.193, *n* = 10). Baker and Wilkinson (2001) found ♀ mean body length 4.97 mm, ♂ 4.50 mm; ♀ mean eye span 2.10 mm, ♂ 2.05 mm.

**Figures 33–36.**
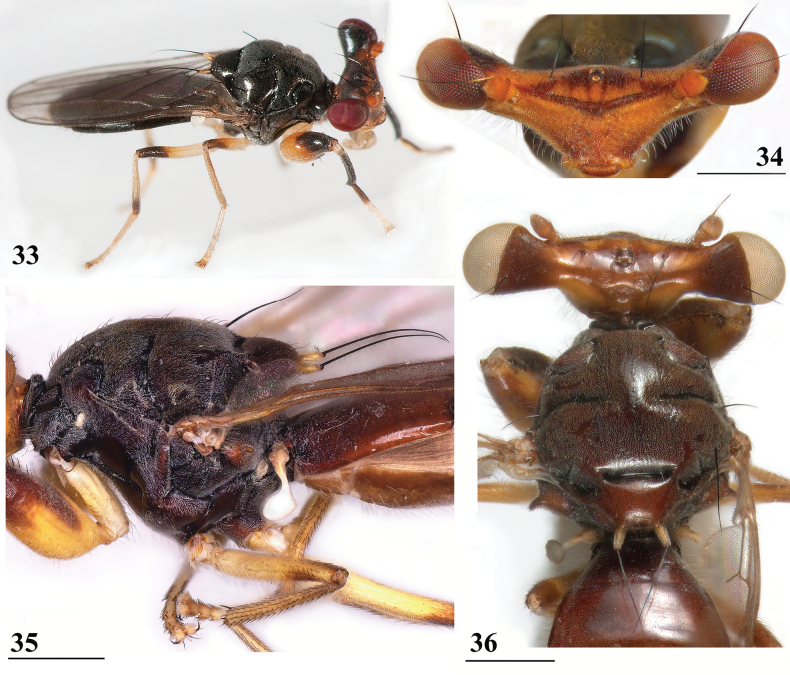
*Sphyracephalabeccarii***33** live photograph, Mikumi, Tanzania (photograph © Stephen Marshall) **34** ♂, head, anterior view, Wadi Maharish, Saudi Arabia **35** ♀, thorax, lateral view, Mahavelo Forest, Madagascar **36** ♀, thorax, dorsal view, Niamey, Niger. Scale bars: 0.5 mm.

**Figure 37.**
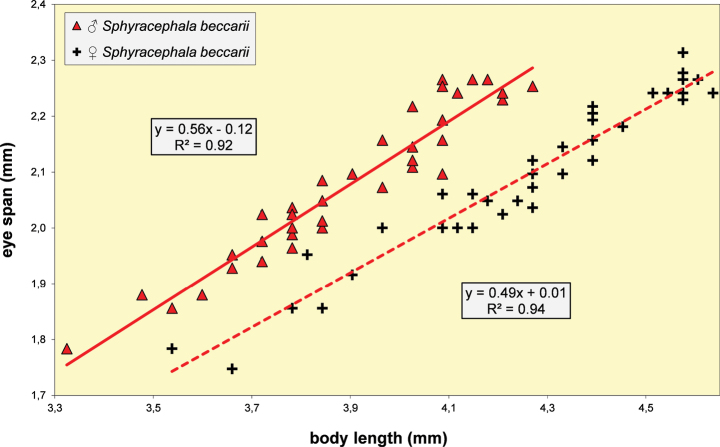
*Sphyracephalabeccarii*, eye span plotted against body length.

***Head***. Central head (Figs 33, 34, 36) brown, arcuate groove dark brown; frons with dark brown semicircular band running from arcuate groove via base of inner vertical seta to ocellar tubercle; stalks dorsally and posteriorly largely blackish; occiput yellowish brown, slightly darker dorsally; head uniformly pruinose (Figs 34, 36), head with a few small black setulae dorsally, ventrally more and longer whitish setulae; arcuate groove distinct blackish; frons with rectangular elevation below ocellar tubercle, grooves laterally of elevation; face flat, no facial teeth, lateroventral corners rounded, facial sulcus absent, but ventral facial edges slightly turned upward medially; eye stalk stout, ~ 0.75–0.80× the widest sagittal eye diameter; eye span very small in both female (49.2% ± SE 0.1% of body length, *n* = 40) and male (53.3% ± SE 0.1% of body length, *n* = 40); a monomorphic species with rate of dimorphism D = 0.07 (Figs 37, 105, 106, Table 2); inner vertical seta long, > 0.4 mm, 1.2× diameter of eye stalk; outer vertical seta long, > 0.3 mm, 0.9× diameter of eye stalk (Figs 34, 36). A drawing of the antenna is provided by Feijen (1983: fig. 5). McAlpine (2011: figs 123, 125) provided a scanning electron microscope picture of the conus of the pedicel and a drawing of funiculus and basal segments of arista. McAlpine (1997: figs 23, 24) discussed the taxonomic importance of the ultrastructure of the face and provided electron micrographs of the lower part of face and parafacials and the microtrichose crazed cuticle of face. In a thesis, Jakobs (1997) studied the fine structure of the optical system. Jakobs measured 30 females and 30 males. She found for ratio eye span/body length in females 44.4% and in males 45.8%, whereas she found a rate of dimorphism D = 0.05, clearly indicating a monomorphic species. Baker and Wilkinson (2001) found for ratio eye span/body length in females 42.3% and in males 45.6%, whereas they found a rate of dimorphism D = 0.20 which still qualifies *S.beccarii* as a monomorphic species.

***Thorax***. Collar, scutum and scutellum blackish brown with few small setulae (Figs 35, 36), scutum and scutellum with fine granulated structure; scutellar spines whitish with brown base; pleura blackish brown, uniformly pruinose; posterior notopleural seta quite long; infra-alar seta long, almost twice length of notopleural seta (Fig. 36); supra-alar carina indistinct; distinct precoxal bridge (Fig. 2); scutal length/scutal width ratio: 0.85; scutellum trapezoid, narrowing distally; scutellar spines small, straight, slightly turned upward, diverging at angle of ~ 60°; scutellar spine/scutellum ratio: 0.50 ± 0.01 (*n* = 25, see Table 3); scutellar spine/length of body ratio: 0.044 ± 0.001 (*n* = 20); apical seta/scutellar spine ratio: 3.89 ± 0.07 (*n* = 25); scutellar length/scutellar width (at base) ratio: 0.69 ± 0.01 (*n* = 20). McAlpine (1997: fig. 27) illustrated the median ventral region of sternopleura, showing double series of pits.

**Figures 38–42.**
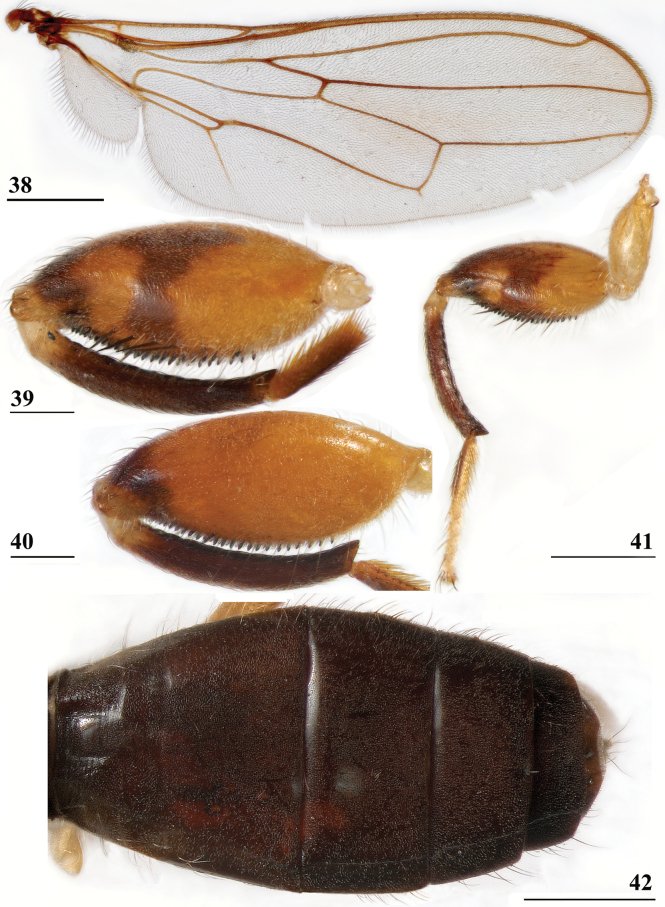
*Sphyracephalabeccarii***38** ♂, wing, Diampwe Malawi **39, 40** ♀, fore femur, Wadi Maharish, Saudi Arabia **39** inner view **40** outer view **41** ♂, Limbe, Malawi, fore leg, inner view **42** ♀, Dedza, Malawi, abdomen, dorsal view. Scale bars: 0.5 mm (**38, 41, 42**); 0.2 mm (**39, 40**).

***Wing***. Transparent with only the faintest brownish hue (Fig. 38); vein CuA+CuP from vein CuP onward extending under angle of 45° to two-thirds of wing margin in straight line; vein M4 continuing distal of crossvein dm-m to one quarter of distance to wing margin; cell cua very narrow, width near base and apex equal (Fig. 38); crossvein h distinct; glabrous area only includes basal quarter of cell br. Wing pictures were provided by van Bruggen (1961: fig. 12). Hilger (2000: fig. 3), Feijen et al. (2017: fig. 11) and Feijen and Feijen (2018: fig. 189). In the drawing by Hilger, cell cua is clearly misrepresented.

***Legs*.** Fore coxa and trochanter very pale, thinly pruinose, with some setulae; fore femur (Figs 39–41) brown, thinly pruinose, apical fifth dark brown on inner and outer side, inner side with dark brown transverse band, dorsally connected to dark apex, sparsely clothed in small setulae; fore tibia dark brown, thinly pruinose; basitarsus brown, other tarsomeres pale, thinly pruinose and with rows of blackish setulae (Fig. 41); mid and hind legs pale brown, femora with dark brown apical third, hind tibia with dark brown apex; fore femur strongly incrassate (Table 2), l/w ratio: 2.48 ± SE 0.02 in ♀ (*n* = 10) and 2.53 ± 0.03 in ♂ *n* = 15); fore femur with two rows of black spinous setae on distal half with in ♀ 7.5 ± SE 0.2 setae (*n* = 16) and in ♂ 6.8 ± 0.2 (*n* = 16) setae, inner row with 6.0 ± 0.1 (*n* = 32) setae and outer row with 1.2 ± 0.1 setae (*n* = 32), two rows of tubercles on distal three-quarters with in ♀ 49.8 ± 0.8 tubercles (*n* = 16) and in ♂ 46.9 ± 0.6 (*n* = 16), inner row with 22.3 ± 0.2 (*n* = 32) tubercles and outer row with 26.1 ± 0.3 (*n* = 32) tubercles. Curran’s (1928) key separating *S.beccarii* from *S.munroi* by the “Tibiæ and tarsi largely or wholly yellowish” for the former and the tibiae and tarsi brown for the latter should be disregarded. Already in Rondani’s (1873) description it was clearly stated “antici .... tibiis, et metatarso nigricantibus”. Collart (1954) in his table indicated as differential characters for *S.beccarii* that the fore femur was strongly incrassate, hind tibia only black at apex and for *S.munroi* that the fore femur was moderately incrassate, hind tibia completely black.

***Preabdomen***. Tergites (Fig. 42) blackish brown, thinly pruinose, small setulae laterally; tergite 1 with very vague transverse ridges, on the meson two parallel, longitudinal grooves (Fig. 42); suture between tergites 1 and 2 just visible; sternites 1–6 brown, all covering the width of the abdomen, sternite 1 and basal half of sternite 2 glossy, other sternites thinly pruinose, sparsely clothed in small white setulae; sternite 1 short, trapezoid; intersternite 1-2 absent, sternite 2 a uniform, slightly trapezoid plate (Fig. 46); sternites 3–6 rectangular.

**Figures 43–47.**
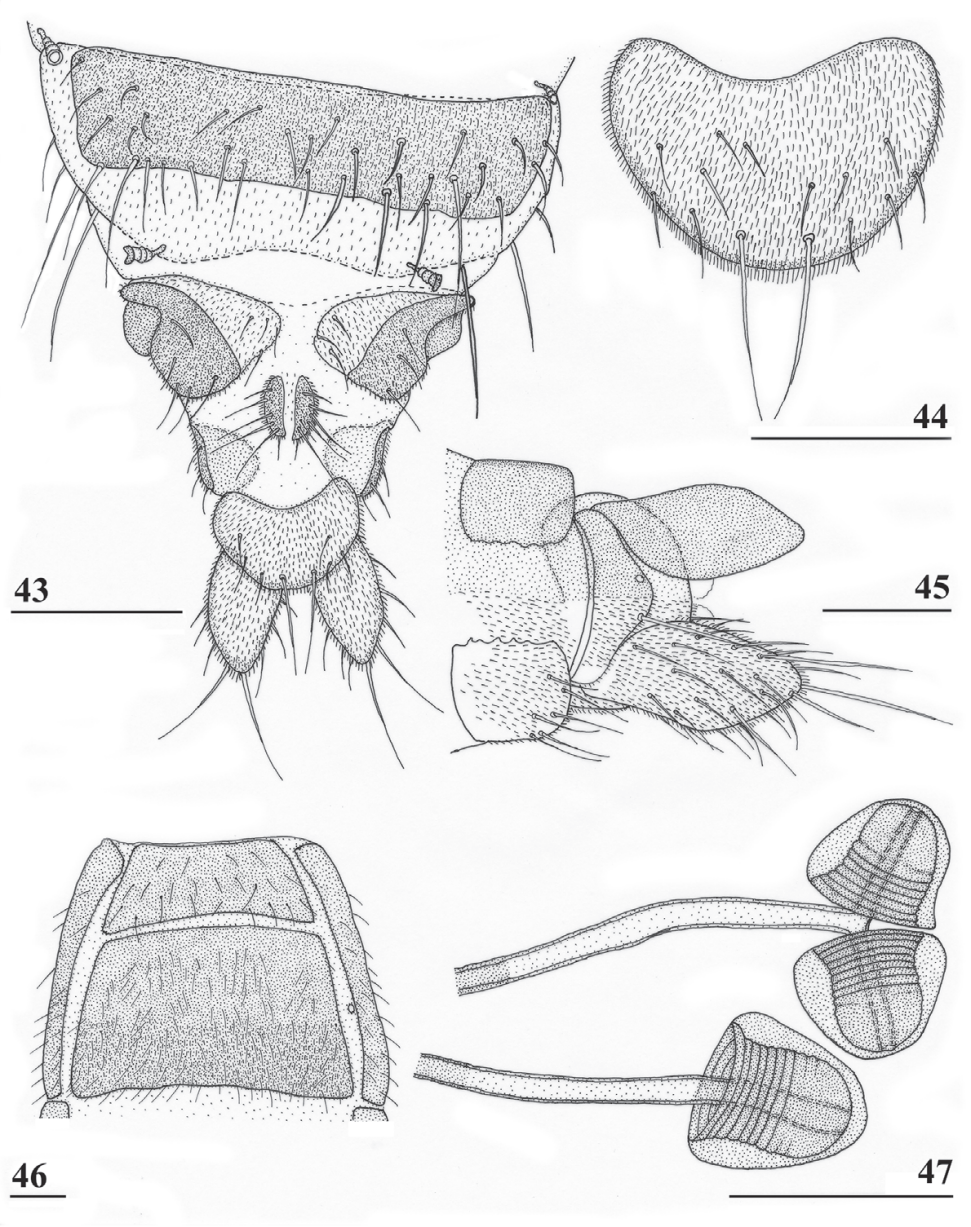
*Sphyracephalabeccarii*, ♀, **43, 44, 47** Maputo. Mozambique, **45, 46** Zomba, Malawi **43** postabdomen, ventral view **44** subanal plate, ventral view **45** tergite 8,10 and cerci, dorsal view **46** sternites 1 and 2, ventral view **47** spermathecae. Scale bars: 0.2 mm (**43, 46**); 0.1 mm (**44, 45, 47**).

***Female postabdomen***. Postabdomen short, broad (Fig. 43); tergite 7 represented by two small, strongly sclerotised, laterally located, sclerites; tergite 8 (Fig. 45) two square, thinly pruinose, sclerites, laterally located, broadly separated on the meson; tergum 10 short, extending posteriorly on the meson, thinly pruinose, one pair of apical setulae; cerci broad, l/w ratio: ~ 1.9, clothed in microtrichia and setulae; sternite 7 consisting of two small, strongly sclerotised, laterally located, angular sclerites, almost touching tergite 7; spiracle 7 located in membrane in between sternites 6 and 7 (Fig. 43); sternite 8 represented by two small sclerites, almost touching on the meson (Fig. 4), near the genital pore, clothed in microtrichia, 6 pairs of long setulae and some small setulae ; subanal plate (Fig. 44) kidney-shaped with rounded apex and rounded anterolateral corners, apex with one pair of longer setulae, clothed in microtrichia and a few pairs of small setulae; spermathecae (Fig. 47) mushroom-shaped with large, bell-shaped, hollow, more sclerotised, striated, inner structure, no protuberances; sclerotised ring of ventral vagina absent. Hilger (2000: figs 4, 5) provides detailed drawings of ventral and lateral views of the postabdomen.

**Figures 48–53.**
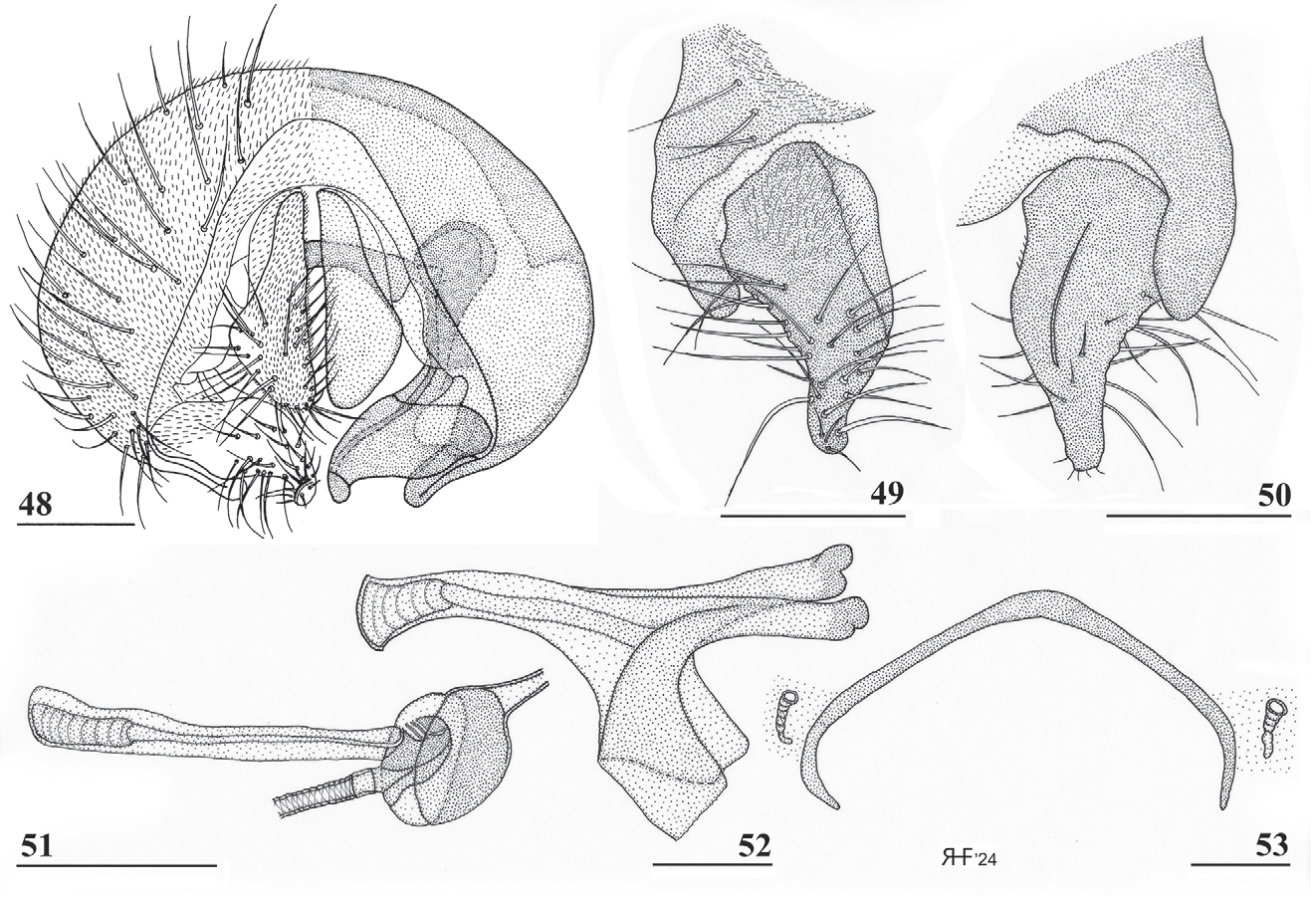
*Sphyracephalabeccarii*, ♂, Diampwe River, Malawi **48** epandrium, cerci, surstyli, posterior view **49** surstylus, outer view **50** surstylus, inner view **51** ejaculatory apodeme and sac **52** phallapodeme **53** syntergosternite 7+8, anterodorsal view. Scale bars: 0.1 mm.

***Male postabdomen***. Syntergosternite 7+8 very slender, extending the width of the abdomen, slightly angular on the meson (Fig. 53); spiracles 7 in membrane; epandrium (Fig. 48) rounded, clothed in microtrichia and ~ 30 pairs of setulae; surstyli articulate, almost encircled by the epandrium, nearly touching on the meson, tapering apically towards an upturned apex (Figs 48–50), outer side with microtrichia on basal third, apical half with ~ 25 setulae (Fig. 49), inner side with only a few small setulae on apical half (Fig. 50); surstyli interconnected via broad processus longi; cerci tapering basally and apically, broadest at one-third from apex (Fig. 48), length/broadest width ratio: 2.8, clothed in microtrichia and on apical half with more than 30 setulae; phallapodeme (Fig. 52) with slender anterior arm, corners pointed, anterior arm slightly longer than posterior arm, lateral processes “vane” broad; phallus broad, short, male genital process hardly sticking out from apex; ejaculatory apodeme straight, very slender (Fig. 51), ejaculatory sac normal-sized.

Hennig (1941b: figs 1, 5d) illustrated the inner genitalia and epandrium with surstylus. Vaillant (1953: figs on p. 12) presents drawings of the postabdomen, but the surstylus is shown as fused to the epandrium, syntergosternite is lacking and the terms ventral and dorsal should be reversed.

Van Bruggen (1961) stated that male genitalia in *Sphyracephala* “are too uniform to facilitate identification of the species”. However, Hennig (1941b) already stated that *S.beccarii* and *S.hearseiana* are extraordinarily similar, but can be distinguished by epandrium and surstyli (see also Figs 48, 49 and Figs 132, 133). Hilger (2000: figs 6, 7) gives drawings of the lateral views of male postabdomen and the phallic complex while Feijen and Feijen (2021: fig. 48) give a posterior view of the epandrium.

***Egg*, *larva*, *and pupa***. Descamps (1957) was the first to note the reticulation of the chorion of the egg as “non strié longitudinalement” and “un fin réseau de petits polygones irréguliers.” Feijen (1989) stated that the absence of longitudinal ridges might represent a apomorphic character for *Sphyracephala*. Meier and Hilger (2000) studying three *Sphyracephala* including *S.beccarii*, stated that the eggs are “entirely covered with hexagonal reticulation, chorion never striated”. They considered the egg ornamentation as a diagnostic character for the genus. Micrographs were provided of dorsal egg, micropyle, posterior pole (Meier and Hilger 2000: figs 9, 10, 11). Descamps (1957) stated that eggs are laid on decomposing plant material. Descamps (1957: pl. 7, figs c–f) described larva and puparium and illustrated larval cephaloskeleton, larval posterior spiracles and puparium.

#### Biology.

Descamps (1957) reared *S.beccarii* in Cameroon. Eggs were laid on decomposing plant matter. Descamps described the saprophagous larvae and the time the various stages take. From egg to fly took approximately two weeks. Descamps indicated that *S.beccarii* often constitute large swarms in the dry season. The flies then disperse at the start of the rainy season. In Algeria, Vaillant (1953) detected among rocks in an oasis swarms of *S.beccarii*. Feijen (1984) found in the dry season in Malawi among rocks in a river bedding a dense mass of *S.beccarii*. After being disturbed they flew up. A single sweep of a net yielded more than 6,000 specimens. The size of the whole mass was estimated to be approximately 100,000 specimens, while the cluster took up an area of less than 0.2 m^2^. Feijen et al. (2017) described and illustrated a cluster of more than 80,000 *S.beccarii* on a tree trunk near a river in Wadi Darbat, Oman. Picker et al. (2019) recorded for South Africa that *S.beccarii* forms groups in moist, rocky places near water. Their idea that these flies mimic small jumping spiders appears unlikely. Mader (2017) mentions for *S.beccarii* the antipodal position during copulation. However, all photographs for *Sphyracephala* and other diopsids show an epipodal position during copulation. Descamps (1957) noted that copulation takes several hours in *S.beccarii*.

Rossi (1990) described *Stigmatomycesbeccarii* (Laboulbeniales) from *S.beccarii*, while *Stigmatomyceselongatus* was described from *S.munroi*. Both new fungi were described from flies from Malawi. It is interesting to note that these two sympatric *Sphyracephala* were parasitised by very different *Stigmatomyces*. Hariri et al. 1998 recorded the presence of the bacteria Type A *Wolbachia* in *S.beccarii*. *Wolbachia* can be associated with female-biased sex ratio distortion. As we report on a female-biased sex ratio in *S.beccarii* from Madagascar only, it would be interesting to compare the *Wolbachia’s* in flies from Madagascar and mainland Africa. Carr (2008) found no evidence for the presence of subfamilies of transposable elements in *S.beccarii*, though three independent lineages were found in *S.babadjanidesi* (as *S.europaea*).

Some minor contradictions are found in the records for the rate of dimorphism for *S.beccarii*. Wilkinson and Taper (1999) considered *S.beccarii* a monomorphic species. They found an eye span/body length ratio of 0.44 for ♀ and 0.47 for ♂ (we found 0.49 and 0.53 respectively). As rate of dimorphism, they gave *D* 0.54–0.35 = 0.19 (we found D 0.56 -0.49 = 0.07, see Fig. 37). The same data were presented in Baker and Wilkinson (2001), but due to a difference in rounding off a *D* of 0.20 is given, while *S.beccarii* was classified as a dimorphic species. Jakobs (1997) found an eye span/body length ratio of 44.4% for ♀ and 45.8% for ♂, and a rate of dimorphism D = 0.05, indicating a monomorphic species. Carr et al. (2005) stated that *S.beccarii* has identical mean eye span in males and females, but female body size is greater, leading to sexual dimorphism in relative eye span. However, that is not the way the rate of dimorphism is determined in Diopsidae, cf. Baker and Wilkinson (2001) and Feijen and Feijen (2009, 2021). Referring to the data of Baker and Wilkinson (2001), Cotton et al. (2004a) considered *S.beccarii* a species with only slight (or weak) sexual dimorphism for eye span. Chapman et al. (2005) considered *S.beccarii* a sexually monomorphic species. Ribak et al. (2009) found for *S.beccarii* no significant differences in the mass-adjusted eye-span between the sexes. Voje and Hansen (2013) used for *S.beccarii* the data of Baker and Wilkinson (2001). Their calculations of allometric slope and intercept were based on the least-squares regression of log eye span as function of log body size. Accordingly, they found that slope and intercept are different between the sexes at the 95% confidence level.

#### Distribution.

*Sphyracephalabeccarii* is known to occur in almost all contiguous Sub-Saharan African countries and Madagascar. We have seen specimens or records from Benin, Botswana, Burkina Faso, Burundi, Cameroon, Chad, D.R. Congo, Eritrea, Eswatini, Djibouti, Ethiopia, Gambia, Ghana, Kenya, Malawi, Mali, Mozambique, Namibia, Niger, Nigeria, Rwanda, Senegal, South Africa, Sudan, Tanzania, Togo, Uganda, Zambia, and Zimbabwe.

*Sphyracephalabeccarii* extends into the Palaearctic Region in Algeria (Bezzi 1922, Hennig 1941b, Séguy 1949, Vaillant 1953). Bezzi referred the Algerian flies to *S.hearseiana*, but Hennig considered that a likely misidentification for *S.beccarii*, while Séguy confirmed that the two flies studied by Bezzi belonged to *S.beccarii*. Vaillant illustrated male genitalia of his Algerian flies and provided reliable information on habitat and swarming. Only his assumptions on the predatory nature of these flies must be rejected. We examined a specimen collected in 1949 in Algeria by Vaillant (ZSM). Papp et al. (1997) considered *S.beccarii* “an Afrotropical species with one questionable record from Algeria”. However, there is no reason to consider the Algerian records as doubtful. Moreover, Séguy (1950) recorded *S.beccarii* for Monts Bagzane in northern Niger, not far from Algeria. *Sphyracephalabeccarii* also extends into the Palaearctic Region in the Arabian Peninsula (Feijen et al. 2017). An extensive number of records were provided for Oman, Saudi Arabia, the United Arab Emirates and Yemen. They discussed the delimitation of the Afrotropical and Palaearctic Regions in the Arabian Peninsula.

### ﻿ Sphyracephala munroi

Taxon classificationAnimaliaDipteraDiopsidae

Curran, 1928

85D699B7-165C-5DBC-9B7D-DA561E2AB675

Figs 1 , 3 , 54–56 , 57–59 , 60 , 61–65 , 66–71 , 72–77 , 105 , 106 , 107 , 108 , 109 , 110–112 , Tables 2 , 3 , 4

Sphyracephala munroi Curran, 1928: 274. Séguy 1949: 75, 1955: 1124; Collart 1954: 330; Descamps 1957: 17; van Bruggen 1961: 415, figs 13, 14, 16; Lindner 1962: 18; Trân 1975: 14, fig. 22b; Cogan and Shillito 1980: 584; Arnaud and Owen 1981: 144; Feijen 1989: 21; Rossi 1990: 3; Baker 1999: 15, figs 1-2, 1-3, 1-7, 2-2, 2-3, table 2-1, App. A; Hariri et al. 1998: 255, table 1; Baker and Wilkinson 2001: figs 2, 3, table1; Baker et al. 2001: figs 1, 2, table1; Meier and Baker 2002: fig. 2; Camerik 2006: 140, table7; Voje and Hansen 2013: figs 1, 2, tab 1; Husak et al. 2013: fig. 2; Holstein 2015: fig. 2 on page 159 (not identified as S.munroi); Feijen and Feijen 2021: 1540, fig. 64.27.
Sphyracephala beccarii (Rondani): Séguy 1949: 75. Collart 1954; 329;
Sphyracephala munroi , Austen in Brunetti, 1928: 273. *Nomen nudum*.

#### Type series.

South Africa: ***holotype***, ♀, Farm Stentor, Barberton, Transvaal [Mpumalanga province, Ehlanzeni District, Nkomazi Local Municipality, 25°33'6"S, 31°22'42"E, 390 m], 7.vi.1925, H. K. Munro (NMSA).

#### Material studied.

Kenya: 5 ♀, 2 ♂, Mt. Elgon, E. side Kaptega r. [~ 1°11'22"N, 34°45'44"E, ~ 2250 m], 26.i.1975, T. Kronestedt (NHRS); Malawi: 24 ♀, 37 ♂, Nyika, Mondwe valley [10°24'S, 33°50'E, 1760 m], vii.1972, D. Munthali (RMNH); 3 ♀, Zomba, near postoffice [15°22'30"S, 35°19'32"E, 980 m], 18.xi.1973; 3 ♂, 25.xi.1973; 9 ♀ 13 ♂, 27.x.1974, all H.R. Feijen (RMNH); 1 ♂, Zomba, Mlunguzi river, [15°22'30"S, 35°19'18"E, 1140 m], 2.viii.1975; 1 ♀, 7.viii.1975, all H.R. Feijen (RMNH); 1 ♂, Mulanje, Likabula river [15°56'19"S, 35°31'13"E, 1045 m], 5.viii.1974, H.R. Feijen (RMNH); 2 ♀, 3 ♂, Fort Lister, along small river [Phalombe District, 15°49'58"S, 35°40'23"E, 1005 m], 5.viii.1974, H.R. Feijen (RMNH); Tanzania: 10 ♀, 19 ♂, Marangu [3°16'35"S, 37°31'11"E, 1480 m], 30.vi.1978, H.R. Feijen (RMNH); 8 ♀, 13 ♂, Arusha [3°22'15"S, 36°41'48"E, 1400 m], 8.vii.1978; 3 ♀, 2 ♂, 3.viii.1978; 242 ♀, 210 ♂, 9.xi.1978; 1 ♂, 4.vii.1987, all H.R. Feijen (RMNH); 10 ♀, 1 ♂, Arusha Centre [3°22'15"S, 36°41'48"E, 1400 m], 8.viii.1978, H.R. Feijen (RMNH); 1 ♂, Arusha, Mnt Meru hotel [~ 3°21'59"S, 36°42'14"E, 1430 m], 10.ii.1984, G.G.M. Schulten (RMNH); Uganda: 1 ♀, Bundibugyu distr., River Kyemahizi, 0°40'12"N, 30°02'06"E, 920 m, 19.3.2012, M. von Tschirnhaus (FBUB). In total 318 ♀ and 307 ♂ were examined, giving a balanced sex-ratio of 100 ♀:97 ♂ (see also Table 4).

#### Diagnosis.

*Sphyracephalamunroi* can be recognised by the following set of characters: head, thorax and abdomen blackish; overall covered with long setulae; brown band below arcuate groove, large brown spots on occiput; eye stalk stout (~ 0.7× the widest sagittal eye diameter), comparatively long and straight for a *Sphyracephala*; very small eye span (2.5–2.7 mm) in ♀ and ♂ (respectively ~ 61% and ~ 67% of body length); very low rate of dimorphism D = 0.33; rectangular basiliform prosternum; apical seta/scutellar spine ratio: ~ 6.1; scutellar spine/scutellum ratio: 0.44; very small, blackish scutellar spines ~ 0.13 mm; transparent wings with brownish tinge; brown fore femur with apical third dark brown, inner side with dark brown longitudinal stripe on central third, incrassate (l/w ratio: 3.63), with two rows of transparent slender spinous setae, inner row with ~ 2.8 setae, outer row with ~ 4.0 setae; tergite 1 with fine transverse ridges and deep circular groove; intersternite 1-2 mesally a small dark sclerite, laterally narrowly connected to main sternite 2; ♀ tergite 7 with 2 large, rectangular sclerites; ♀ sternite 7 forming two rectangular sclerites with posterior extensions; ♀ cerci elongate, l/w ratio: ~ 4.6; ♀ sternite 8 forming two large rectangular sclerites; no real sclerotised ring, but mesally a tiny structure with thin lateral extensions; surstyli articulate, ventromedially directed, parallel-sided, on medial side hollow, no microtrichia, outer side clothed in setulae, inner side with a comb of fine, small setulae. *Sphyracephalamunroi* belongs to the *S.brevicornis* species group and comes closest to *S.babadjanidesi*.

#### Redescription.

The following redescription considers the original description by Curran (1928), the table of differences between *S.beccarii* and *S.munroi* by Collart (1954), and description and illustrations by van Bruggen (1961: figs 13, 14, 16).

***Measurements***. Body length ♀ 4.2 mm ± SE 0.0 (range 3.7–4.7, *n* = 40), ♂ 4.1 mm ± 0.0 (range 3.7–4.5, *n* = 40), eye span ♀ 2.5 mm ± 0.0 (range 2.2–3.0, *n* = 40), ♂ 2.7 mm ± 0.0 (range 2.2–3.2, *n* = 40); wing length ♀ 3.5 mm ± 0.0 (range 3.2–3.7, *n* = 10), ♂ 3.6 mm ± 0.1 (range 3.3–3.8, *n* = 10); length of scutellar spine ♀ 0.13 ± 0.00 (range 0.10–0.14, *n* = 10), ♂ 0.13 mm ± 0.00 (range 0.12–0.14, *n* = 10). Baker and Wilkinson (2001) found ♀ mean body length 5.96 mm, ♂ 5.29 mm, ♀ mean eye span 2.88 mm, ♂ 2.86 mm.

**Figures 54–56.**
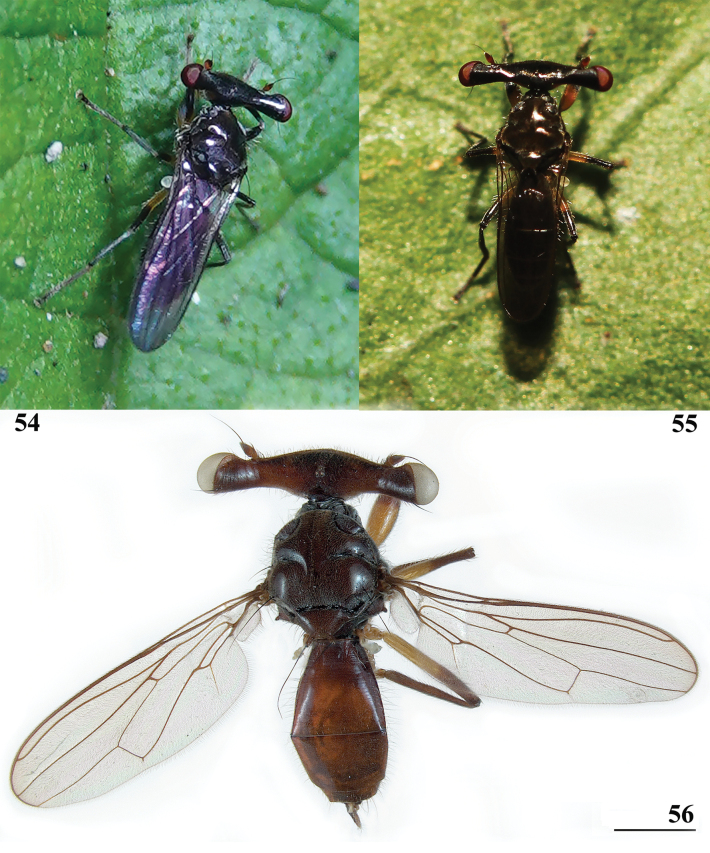
*Sphyracephalamunroi*, **54, 55** live photographs **54** Arusha N.P., Tanzania (photograph © Donyo Gabriel) **55** Matema, Tanzania (photograph © Martin Grimm) **56** ♀, Arusha, Tanzania, habitus, dorsal view. Scale bar: 1 mm (**56**).

**Figures 57–59.**
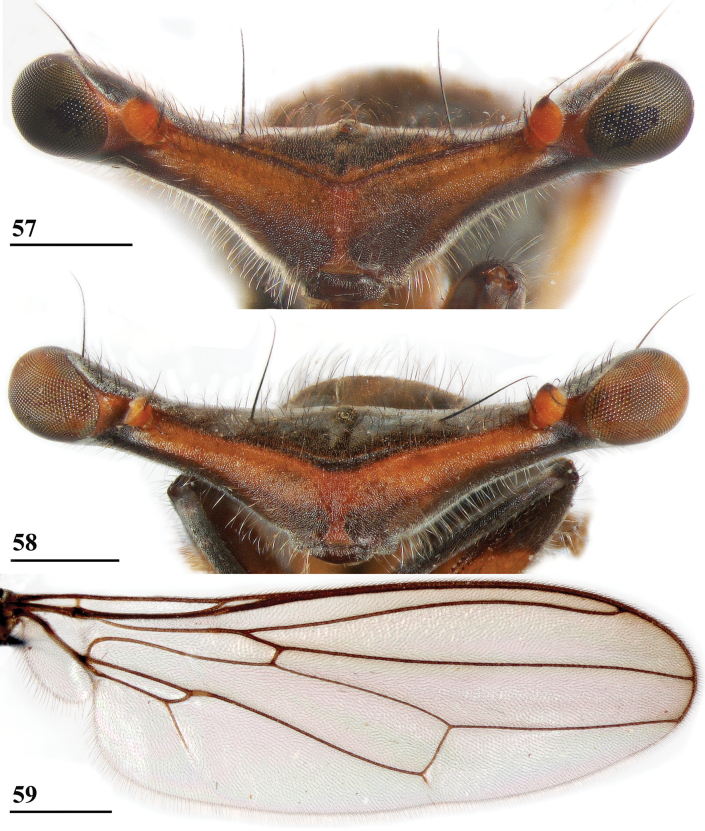
*Sphyracephalamunroi***57** ♀, head, anterior view, Zomba, Malawi **58** ♂, head, anterior view, Arusha, Tanzania **59** ♀, wing, Arusha. Scale bars: 0.5 mm.

**Figure 60.**
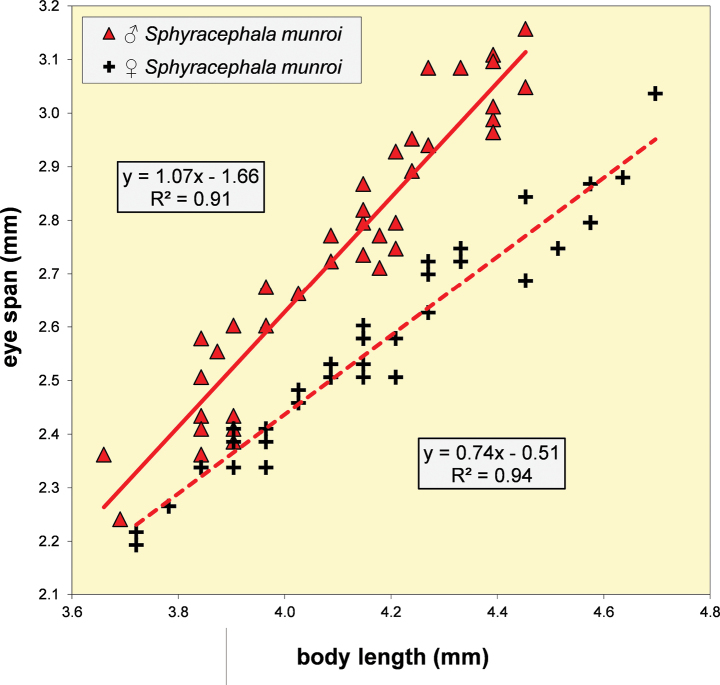
*Sphyracephalamunroi*, eye span plotted against body length.

***Head***. Central head (Figs 54–58) and stalks blackish brown with broad brown band below arcuate groove running from antenna to antenna, basal ventral sections of stalks brown, occiput yellowish brown with dark brown edges; head uniformly pruinose (Figs 57, 58) except for glossy ventral part of clypeus and glossy ventral edge of occiput, head quite setulose; arcuate groove not very distinct, narrow and blackish; frons with slight elevation below ocellar tubercle; face flat, no facial teeth, lateroventral corners rounded, facial sulcus absent, but ventral facial edges slightly turned upward medially; eye stalk stout, ~ 0.7× the widest sagittal eye diameter; eye span very small in both female (61.3% ± SE 0.2% of body length, *n* = 40) and male (66.7% ± SE 0.5% of body length, *n* = 40); a dimorphic species with a very low rate of dimorphism D = 0.33 (Figs 60, 105, 106, Table 2); inner vertical seta long, close to 0.5 mm, 1.5× diameter of eye stalk; outer vertical seta long, close to 0.4 mm, 1.1× diameter of eye stalk (Figs 57, 58). Curran (1928) indicated long ocellar setae, but that must be an error. Baker and Wilkinson (2001) found eye span in female to be 48.3% of body length and in male 54.1%, whereas they found a rate of dimorphism of 0.18 which would indicate *S.munroi* as a monomorphic species.

***Thorax***. Collar blackish, pruinose; scutum, scutellum and scutellar spines uniformly blackish, pruinose and quite setulose (Fig. 56) [Curran (1928) described the scutellar spines as brown, but Collart (1954) and van Bruggen (1961) named the black spines a differential character]; pleura blackish, uniformly pruinose; posterior notopleural seta medium-sized; infra-alar seta long, 3× the length of notopleural seta (Fig. 56); supra-alar carina distinct; basiliform prosternum (Fig. 1) large, rectangular, laterally close to propleuron but clearly distinct [Feijen (1989) noted a precoxal bridge for *S.munroi*, but that is an error.]; scutal length/scutal width ratio: 0.87; scutellum trapezoid, strongly narrowing distally; scutellar spines very small, straight, slightly turned upward, diverging at angle of ~ 75°; scutellar spine/scutellum ratio: 0.44 ± 0.01 (*n* = 20, see Table 3); scutellar spine/length of body ratio: 0.033 ± 0.001 (*n* = 20); apical seta/scutellar spine ratio: 6.10 ± 0.12 (*n* = 19); scutellar length/scutellar width (at base) ratio: 0.60 ± 0.01 (*n* = 20).

***Wing***. Transparent with a faint brownish tinge, especially apically (Figs 56, 59); vein CuA+CuP from vein CuP onward extending under angle of 45° to halfway wing margin in straight line; vein M4 continuing distal of crossvein dm-m to one third of distance to wing margin; cell cua narrow, basally acute, apically rounded (Fig. 59); crossvein h distinct; glabrous area only includes anterior half of cell bc.

**Figures 61–65.**
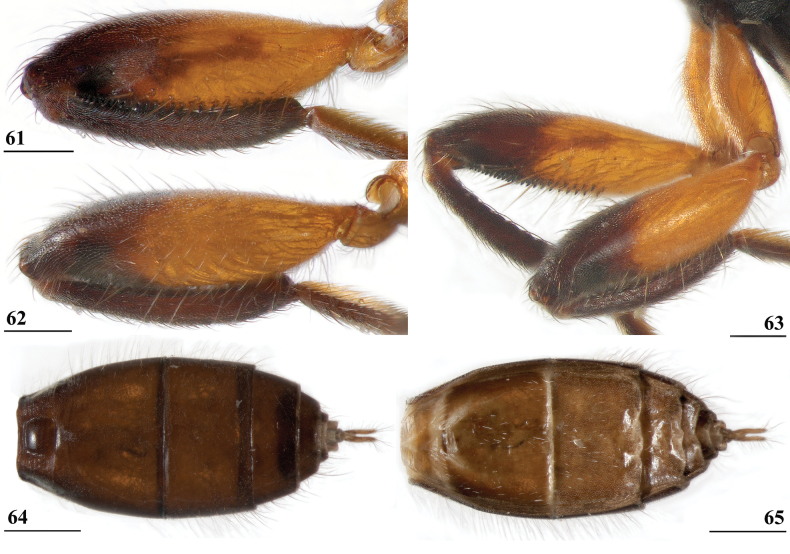
*Sphyracephalamunroi*, ♀, **61, 62** Marangu, Tanzania **63–65** Arusha, Tanzania **61** fore femur, inner view **62** fore femur, outer view **63** fore legs, lateral view **64** abdomen, dorsal view **65** abdomen, ventral view. Scale bars: 0.5 mm (**64, 65**); 0.2 mm (**61–63**).

***Legs*.** Fore coxa and trochanter pale brown (Fig. 63), coxa densely pruinose on anteriorly directed side, setulose; fore femur (Figs 61–63) pale brown, apical third dark brown on inner and outer side, inner side with characteristic dark brown longitudinal stripe on central third (Fig. 61), outer side thinly pruinose, inner side with subapically a densely pruinose depression, thinly pruinose dorsally and on inner side, clothed in pale setulae; fore tibia and tarsus dark brown, thinly pruinose and with rows of blackish setulae; mid and hind legs pale brown, femora with dark brown apical third, tibiae and tarsi brown; fore femur incrassate (Table 2), l/w ratio: 3.63 ± SE 0.06 in ♀ (*n* = 10) and 3.63 ± 0.03 in ♂ *n* = 10); fore femur with two rows of rather transparent, slender spinous setae (almost setula-like and difficult to count, especially on inner side) on distal half with in ♀ 6.0 ± SE 0.4 setae (*n* = 12) and in ♂ with 7.2 ± 0.3 (*n* = 12), inner row with 2.8 ± 0.1 (*n* = 24) setae and outer row with 4.0 ± 0.1 setae (*n* = 24), two rows of tubercles on distal three-quarters (Fig. 63) with in ♀ 46.5 ± 0.8 tubercles (*n* = 11) and in ♂ with 44.5 ± 0.6 (*n* = 11), inner row with 24.2 ± 0.3 (*n* = 23) tubercles and outer row with 21.3 ± 0.3 (*n* = 23) tubercles.

Curran’s (1928) key separating *S.beccarii* from *S.munroi* by the “Tibiæ and tarsi largely or wholly yellowish” for the former and the tibiae and tarsi brown for the latter should be disregarded. Collart (1954) in his tabulated key indicated useful differential characters: for *S.beccarii* “Fémurs antérieurs: fortement grossis. Tibias postérieurs: noirs à l’extrémité seulement.” and for *S.munroi* “Fémurs antérieurs: modérément épaissis. Tibias postérieurs: entièrement noirs.”

**Figures 66–71.**
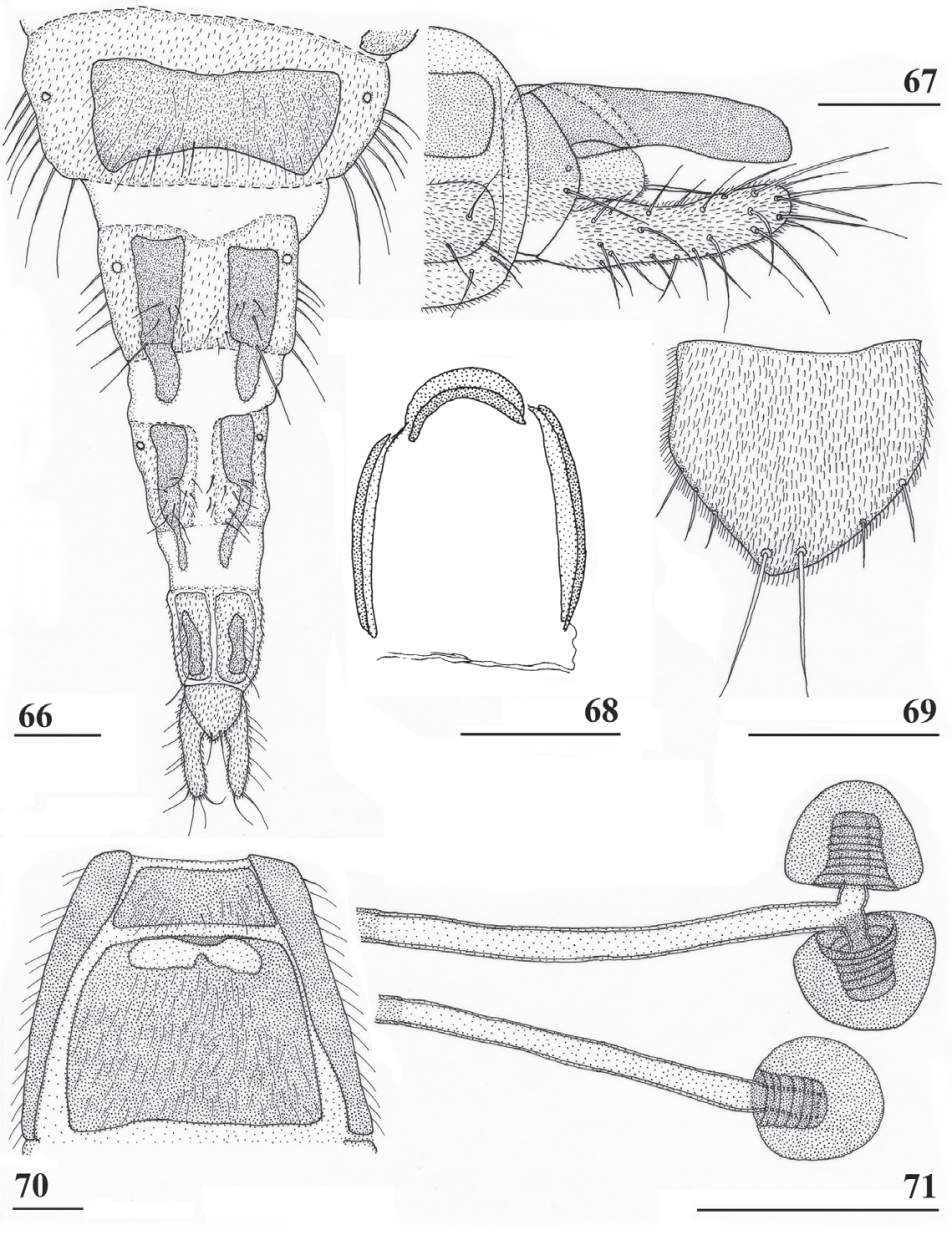
*Sphyracephalamunroi*, ♀, **66, 68–70** Arusha, Tanzania, **67, 71** Zomba, Malawi **66** postabdomen, ventral view **67** tergite 8, 10 and cerci, dorsal view **68** sclerotised ring **69** subanal plate, ventral view **70** sternite 1, intersternite 1-2 and sternite 2, ventral view **71** spermathecae. Scale bars: 0.2 mm (**66, 70**); 0.1 mm (**67, 69, 71**); 0.05 mm (**68**).

***Preabdomen***. Tergites (Fig. 64) blackish brown, very thinly pruinose, almost glossy, setulose, especially laterally; tergite 1 with fine transverse ridges and on the meson a large, deep, circular groove (Fig. 64); suture between tergites 1 and 2 visible; tergites 2–6 rectangular; sternites 1–6 brown, all covering the width of the abdomen, clothed in small white setulae, sternites 1 and 2 glossy, sternites 3–6 thinly pruinose; sternite 1 short and trapezoid (Figs 65, 70); intersternite 1-2 mesally a slender dark sclerite, laterally narrowly connected to main sternite 2; sternites 2–5 rectangular sclerites (Figs 65, 66); sternite 6 consisting of two long, elongate sclerites, posteriorly with less sclerotised, narrower extensions (Fig. 66).

***Female postabdomen***. Postabdomen long, narrow (Fig. 66); tergite 7 represented by two rectangular sclerites, well separated mesally; tergite 8 two rectangular, thinly pruinose, sclerites, separated on the meson; tergum 10 short, posteriorly rounded, thinly pruinose but laterally glabrous, one pair of apical setulae; cerci elongate, l/w ratio: ~ 4.6, clothed in microtrichia and setulae (Fig. 67); sternite 7 consisting of two rectangular, elongate sclerites with long, narrow posterior extension; spiracle 7 in membrane; sternite 8 represented by two large rectangular sclerites, separated on the meson, almost taking up the width of the abdomen, posterolaterally more sclerotised (Fig. 3), clothed in microtrichia and 12 pairs of setulae; subanal plate (Fig. 69) pentagonal with rounded corners, apex with one pair of longer setulae, clothed in microtrichia and a few pairs of small setulae; spermathecae (Fig. 71) mushroom-shaped with medium-sized, bell-shaped, hollow, more sclerotised, striated, inner structure, no protuberances; no real sclerotised ring of ventral vagina, but mesally a tiny curved structure with thin lateral extensions with a very thin, transparent connection between posterior tips (Fig. 68).

**Figures 72–77.**
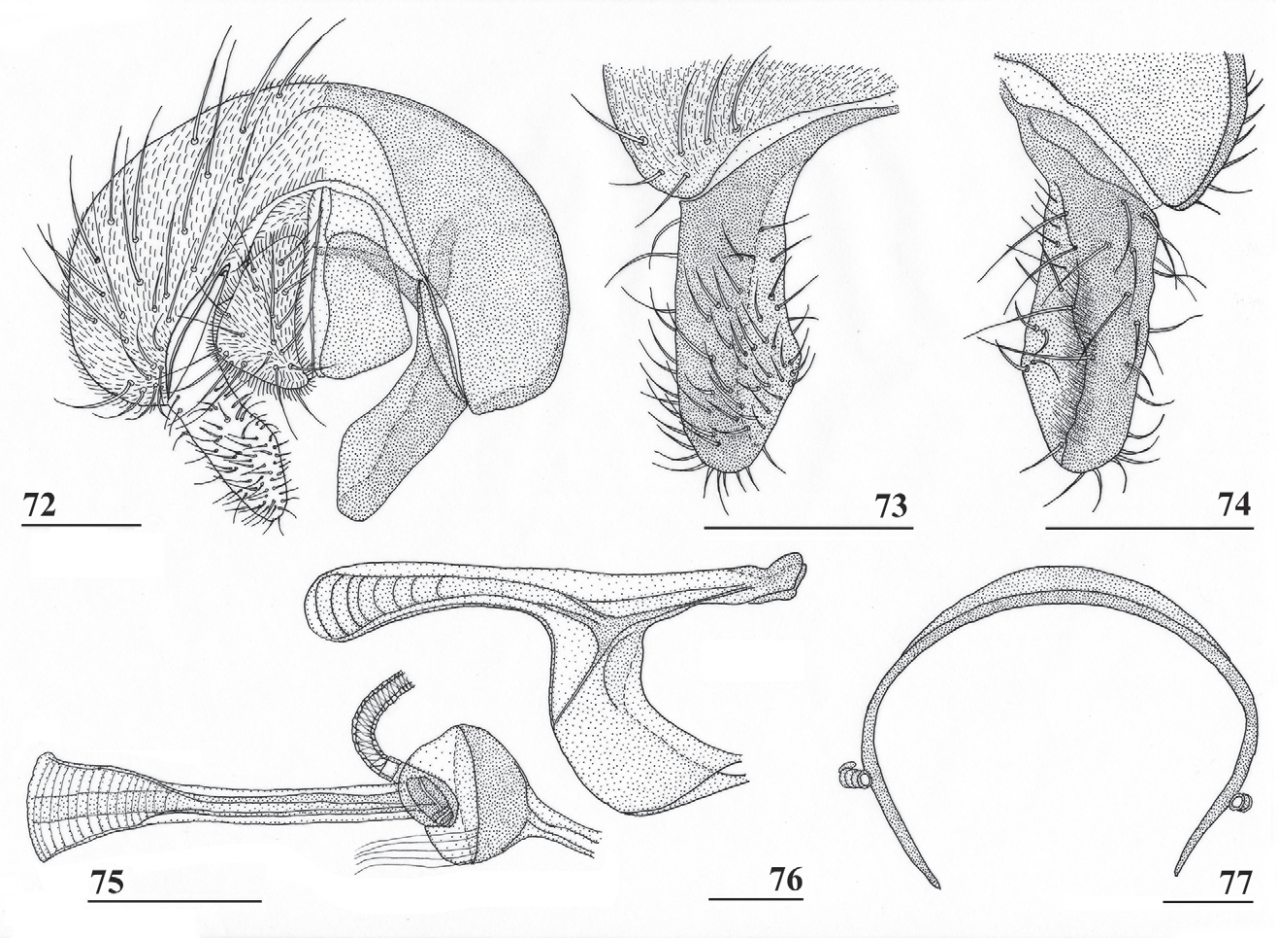
*Sphyracephalamunroi*, ♂, Zomba, Malawi **72** epandrium, cerci, surstyli, posterior view **73** surstylus, outer view **74** surstylus, inner view **75** ejaculatory apodeme + sac **76** phallapodeme **77** syntergosternite 7+8, posterior view. Scale bars: 0.1 mm.

***Male postabdomen***. Syntergosternite 7+8 slender, on both sides extending on the venter, (Fig. 77), spiracles 7 connected to sclerite well before its apices; epandrium (Fig. 72) rounded, clothed in microtrichia and ~ 25 pairs of setulae; surstyli (Figs 73, 74) articulate, l/w ratio: ~ 2.8, ventromedially directed, almost parallel-sided, apically rounded, on medial side hollow (scoop-like), no microtrichia, outer side clothed in setulae on distal three-quarters (Fig. 73), inner side with a few larger setulae on distal half, a comb of fine, small setulae along central ridge of “scoop” (Fig. 74); surstyli interconnected via slender processus longi; cerci (Fig. 72) broadening towards apex, length/broadest width ratio: 1.6, clothed in microtrichia and ~ 20 setulae; phallapodeme (Fig. 76) with club-shaped anterior arm, apically rounded, anterior arm 1.5× longer than posterior arm, lateral processes broad; phallus broad, short, male genital process hardly sticking out from apex; ejaculatory apodeme straight, slender, apically twice as broad as basally (Fig. 75), ejaculatory sac normal-sized.

#### Biology.

Compared with *S.beccarii*, not much is known about the second Afrotropical species *S.munroi*. The large numbers of flies collected in Arusha, Tanzania, clearly show that *S.munroi* can also show gregarious behaviour. However, real clusters (see Feijen et al. 2017) have not yet been reported. Rossi (1990) described *Stigmatomycesbeccarii* (Laboulbeniales) from *S.beccarii*, while *Stigmatomyceselongatus* was described from *S.munroi*. Both new fungi were described from flies from Malawi. It is interesting to note that these *Sphyracephala* were parasitised by very different *Stigmatomyces*. Hariri et al. 1998 recorded the presence of bacteria Type A *Wolbachia* in *S.munroi*. *Wolbachia* can be associated with female-biased sex ratio distortion, but such a sex ratio has not been found in *S.munroi* (Table 4). Feijen (1989) reported on a *S.munroi* with the venter covered with closely packed mites. In the specimens examined, 3 ♀ and 1 ♂ from 9.xi.1978, Arusha, had the venter covered with mites. Camerik (2006) described the mite *Pediculasterkilimanjarensis* (Acari: Siteroptidae) from *S.munroi* collected in Tanzania.

#### Distribution.

Angola, D.R. Congo, Eswatini, Kenya, Malawi, Mozambique, South Africa, Tanzania, Uganda, Zimbabwe. It appears that *S.munroi* is confined to Eastern and Southern Africa. However, we have seen some records from West Africa, but those need confirmation. *Sphyracephalamunroi* was, in general, only collected from higher altitudes of 900–2250 m. Only the type locality in South Africa is from a lower altitude (390 m).

### ﻿ Sphyracephala nigrimana

Taxon classificationAnimaliaDipteraDiopsidae

Loew, 1873

C511DB94-7BD6-53C8-868D-7525FAFC0BA3

Figs 78–79 , 80–82 , 83–87 , 88–93 , 94–99 , 100 , 105 , 106 , 107 , 108 , 109 , 110 , 112 , Tables 2 , 3

Sphyracephala nigrimana Loew, 1873: 103. Osten Sacken 1882: 235; Bezzi 1922: 69; Lindner 1925: 167; Brunetti 1928: 273; Frey 1928: 70; Hennig 1941a: 59, 62, fig. 10b (scutellar spine indicated as from type), 1941b: 7, figs 5, 7; Steyskal 1972: 13; Yang and Chen 1988: 142; Feijen 1989: 67; Yang and Chen 1998: 474; Hilger 2000: 340; Simova-Tošić and Stojanović 2000: 149; Nartshuk 2003: 179, pl. 44 fig. 12, 2017: 129; Sidorenko 2004: 456, fig. 228; Hua 2006: 158; Mader 2017: 108; Feijen et al. 2018: 206.
Sphyracephala brevicornis (Say): Portschinsky 1871: 287, “dans les environs de Vladivostok” [near Vladivostok, Russia]. Loew 1873: 103; Bezzi 1922: 69; Hennig 1941b: 7; Nartshuk 2017: 129.
Non Sphyracephalanigrimana: Liu 2009: 67, figs 3e, 36, 1 ♂, Hongmao Village, Yuanmen, Baisha, Hainan, 19.x.2007, Yang Ding.

#### Type series.

Russia: multiple specimens, Nebenfluß des Amur [side river of the Amur], A. Fedtschenko [Alexei Pavlovich Fedchenko, 1844–1873]. Hennig (1941b) studied several specimens designated as *S.nigrimana* Loew in the Loew Collection (ZMHB). Although these specimens carried no location information, Hennig stated they could perhaps be considered “Typen” (types). According to Sven Marotzke (ZMHB, pers. comm., 2024) five specimens could be found: two pins with each one specimen glued to a card, and one pin with three specimens glued to a card. All three cards carried the information “Coll. Loew”. In addition, on one card with a single specimen was written “*Sphyracephalanigrimana* Loew”, while on the card with three specimens was added “Post Dubinskiy 23.iii.1870”. Two microscopic slides were also found labelled “innerer Kopul.-App.” [inner genital structure] and “Hypopygium” [epandrium]. There can be no doubt that these specimens represent the flies studied and illustrated by Hennig (1941b: figs 2, 5A, 7) and that the slides were made by him. The information “Post Dubinskiy, 23.iii.1870” is new. The collecting date looks reliable and fits the time line. The location Post Dubinskiy could not be traced.

#### Material studied.

Russia: 1 ♀, 1 ♂, зап. Кедровая Падь., Приморье Городков 19.x.1968, Усадода (?) На стене (RMNH) [Primorye (Primorsky Krai - region), Kedrovaya Pad Nature Reserve (Korean Pine Valley Reserve or Cedar Reserve), on the wall, 43°05'N, 131°30'E, 40–700 m, 19.x.1968, Gorodkov]. Kiril Borissovich Gorodkov (1932–2001) was a Russian entomologist.

#### Diagnosis.

*Sphyracephalanigrimana* can be recognised by the following set of characters: central head brown, thorax and abdomen blackish; clothed in small setulae; head subtriangular in anterior view; eye stalk very short, very stout (~ 1.1× the widest sagittal eye diameter); very small eye span in ♀ and ♂, ~ 39% of body length; assumed sexual monomorphy with regard to eye span; rectangular basiliform prosternum with medial groove; apical seta/scutellar spine ratio: ~ 3.0; scutellar spine/scutellum ratio: ~ 0.7; small, pale scutellar spines ~ 0.21 mm; transparent wing with pattern of dark brown spots including apical spot, central crossband and basal spots; brown fore femur with apical half darker brown, strongly incrassate (l/w ratio: 2.7–2.9), with two rows of spinous setae (more transparent on outer side); tergite 1 with distinct transverse ridges and vague circular groove, tergite 2 anteriorly with small triangle with transverse ridges; intersternite 1-2 a broad band, laterally connected to main sternite 2; ♀ tergite 7 with 2 small, laterally located, sclerites; ♀ sternite 7 forming 2 small, rounded sclerites with posterior extensions; ♀ cerci rather elongate, l/w ratio: ~ 3.3; ♀ sternite 8 forming 2 large rectangular sclerites; well-developed sclerotised ring, triangular to rounded; surstyli articulate, ventrally directed, parallel-sided, l/w ratio: ~ 4.7, on medial side scope-like, no microtrichia, inner and outer side clothed in setulae. *Sphyracephalanigrimana* comes closest to the two Nearctic *Sphyracephala*.

#### Redescription.

The following redescription considers the original description by Loew (1873), descriptions by Hennig (1941a, b) and illustrations by Nartshuk (2003) and Sidorenko (2004). In his description, Loew especially indicated the differences with the Nearctic *S.brevicornis*.

***Measurements***. Body length ♀ 3.97 mm, ♂ 4.03 mm; eye span ♀ 1.52 mm, ♂ 1.59 mm; wing length ♀ 2.99 mm, ♂ 3.11 mm; length of scutellar spine ♀ 0.22 mm, ♂ 0.20 mm (Tables 2, 3). Loew (1873) only indicated that *S.nigrimana* was of the same size as *S.brevicornis*, but that the eye span was smaller. Feijen (1989) gave for *S.brevicornis* a mean length of body of 4.41 mm for ♀ and 4.25 mm for ♂, while the mean eye span came to 1.93 mm in ♀ and 1.88 mm in ♂. This agrees with Loew’s observation about the relatively smaller eye span in *S.nigrimana*. The drawing by Hennig (1941b: fig. 2)) indicates a body length of 3.44 mm and an eye span of 1.37 mm for an unsexed fly. Sidorenko (2004) gives a body length of 3.5–4.2 mm.

**Figures 78, 79.**
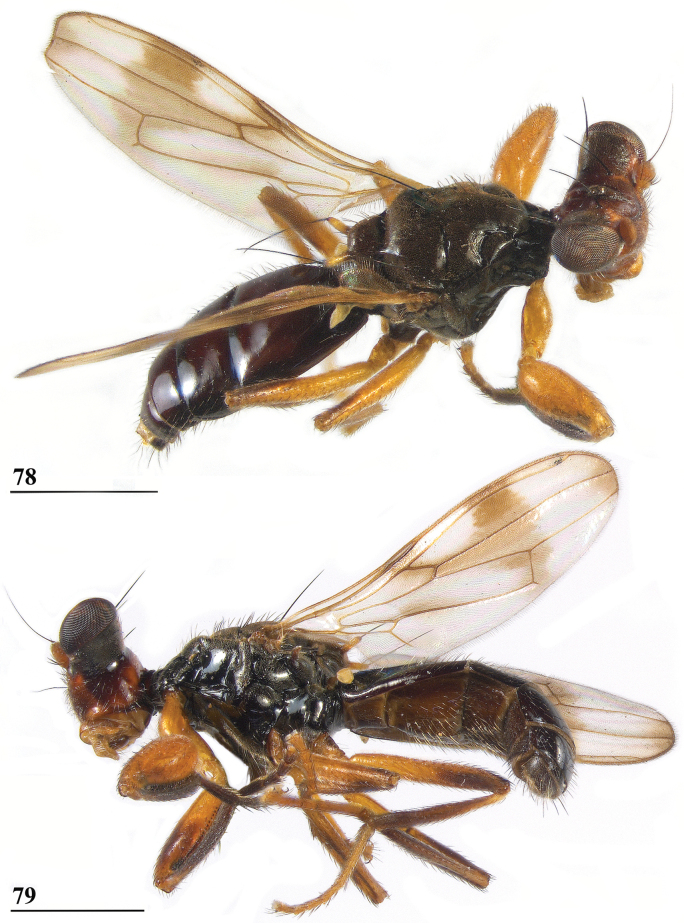
*Sphyracephalanigrimana*, Kedrovaya Pad, Russia **78** ♀, habitus, dorsolateral view **79** ♂, habitus, ventrolateral view. Scale bars: 1 mm.

**Figures 80–82.**
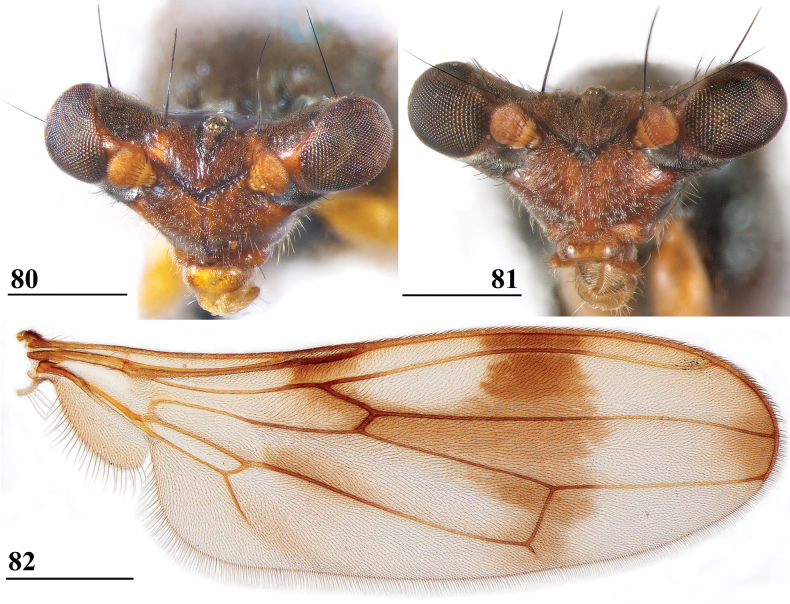
*Sphyracephalanigrimana*, Kedrovaya Pad, Russia **80** ♀, head, anterior view **81** ♂, head, anterior view **82** ♂, wing. Scale bars: 0.5 mm.

***Head***. Subtriangular in anterior view (Figs 80, 81); central head dark brown (Figs 78–81), stalks blackish, below the inner vertical setae and laterally of the antennae small yellowish brown spots; face thinly pruinose (Figs 80, 81) with laterally some whitish setulae; frons (Figs 80, 81) and ocellar tubercle thinly pollinose; arcuate groove distinct, narrow and blackish; facial sulcus shallow and indistinct, no facial teeth, lateroventral corners of face rectangular; clypeus more yellowish brown and more glossy; occiput glossy ventrally of ocellar tubercle, some white setulae dorsally and ventrally (Figs 79, 80); eye stalk very stout, ~ 1.05–1.10× the widest sagittal eye diameter; eye span very small (Table 2) in both female (38.3% of body length) and male (39.5% of body length), Hennig’s (1941b) drawing shows a ratio of 39.8% for an unknown sex; the two data points for eye span/body length (Figs 100, 105, 106) are just below the allometric lines for *S.brevicornis* and *S.subbifasciata*, so it appears most likely that *S.nigrimana* is also a monomorphic species; inner and outer vertical setae long, close to 0.5 mm, approx. equal in length to diameter of eye stalk (Figs 80, 81).

***Thorax***. Collar black, pruinose with laterally tiny glossy spots; scutum and scutellum uniformly black, pruinose (Fig. 78), scutellar spines pale but darker basally and apically, covered with tiny setulae (Figs 78, 79, 84); scutum and scutellum clothed with small blackish setulae; pleura dark black, largely pruinose, glossy sections (Fig. 79) include anepisternum and anepimeron (except for posterior edge of anepisternum and dorsal edge of both sclerites), katepisternum (except dorsoposteriorly) and meron (except dorsal and posterior edges); posterior notopleural seta and infra-alar seta long (Fig. 79), infra-alar seta slightly larger than posterior notopleural seta, supra-alar carina just visible; basiliform prosternum large, rectangular, with medial groove, prosternum laterally close to propleuron but clearly distinct; scutal length/scutal width ratio: 1.0; scutellum trapezoid; scutellar spines very small, straight, almost aligned with dorsal plane of scutellum, diverging at angle of ~ 75°; scutellar spine/scutellum (Table 3) ratio: 0.75 in ♀ and 0.65 in ♂; scutellar spine/length of body ratio: 0.055 in ♀ and 0.051 in ♂; apical seta/scutellar spine ratio: 3.22 in ♀ and 2.82 in ♂; scutellar length/scutellar width (at base) ratio: 0.63 in ♀ and 0.72 in ♂.

***Wing***. Almost transparent with distinct pattern of dark brown spots (Figs 78, 79, 82); apex with small spot in cells r2+3 and r4+5; central irregular crossband running from anterior margin to posterior margin near M4; crossband darker anteriorly in cells r1 and r2+3, broadens strongly proximally in cell r4+5 to include crossvein r-m, and narrows posteriorly around crossvein dm-m; from crossvein dm-m a small band runs anteriorly to cell sc; a spot centrally in cell bm+dm along vein M4; a vague spot in cell m4 distally of vein CuA+CuP; alula vaguely brown infuscated; vein CuA+CuP from vein CuP onward extending under angle of 30° to halfway wing margin in slightly curved line; vein M4 continuing distal of crossvein dm-m to less than halfway wing margin; cell cua subrectangular (Fig. 82); crossvein h indistinct; glabrous area only includes small basal spot in cell br.

**Figures 83–87.**
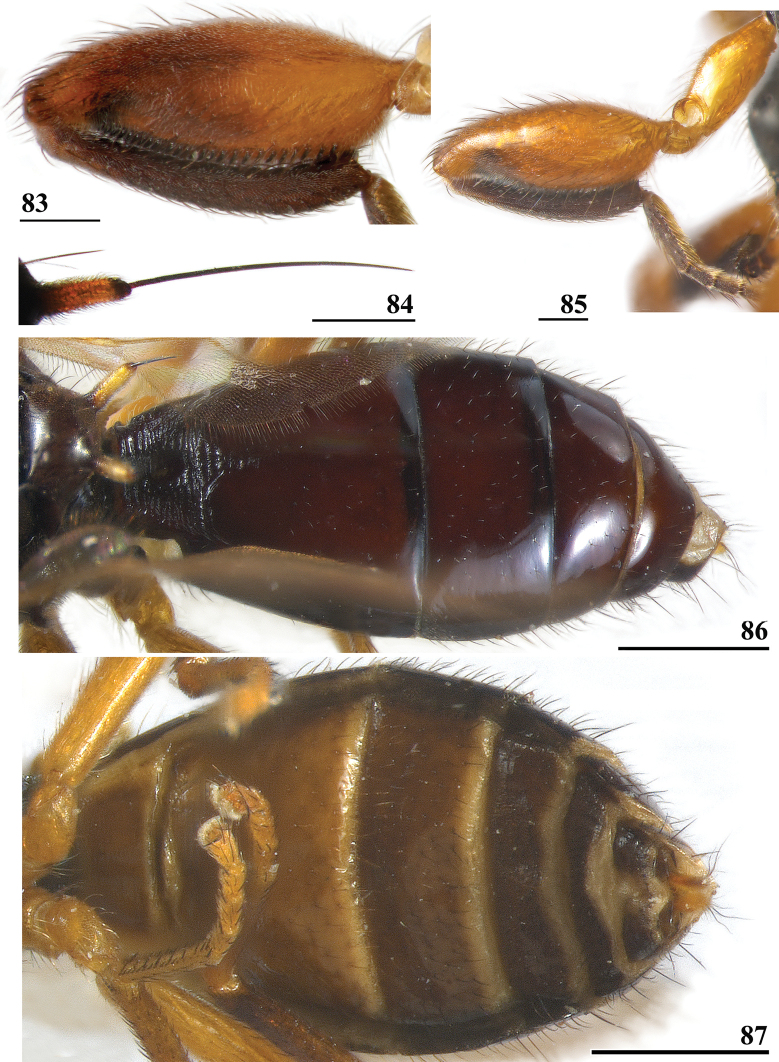
*Sphyracephalanigrimana*, Kedrovaya Pad, Russia **83** ♂, fore femur, inner view **84** ♂, scutellar spine, apical bristle **85** ♂, fore leg, outer view **86** ♀, abdomen, dorsal view **87** ♀, abdomen, ventral view. Scale bars: 0.5 mm (**86, 87**); 0.2 mm (**83–85).**

***Legs*.** Fore coxa and trochanter brown, thinly pruinose on inner side, with some whitish setulae; fore femur (Figs 83, 85) brown, irregularly dark brown on distal half of inner side, thinly pruinose dorsally and on inner side, clothed in dark setulae; fore tibia and tarsus blackish brown, hence the specific epithet *nigrimana* (Figs 79, 85), thinly pruinose and with rows of blackish setulae; mid and hind legs brown, femora with dark brown spots on distal third of inner and outer side, tibiae darker brown; fore femur strongly incrassate, l/w ratio: 2.9 in ♀ and 2.7 in ♂ (Table 2), two rows of spinous setae on distal half with 6.0 ± 0.0 setae (*n* = 4), inner row with 4.0 ± 0.0 setae and outer row with 2.0 ± 0.0 setae, two rows of tubercles on distal five-sixth with 52.8 ± 0.6 tubercles (*n* = 4), inner row with 25.3 ± 0.3 (*n* = 4) tubercles and outer row with 27.5 ± 0.5 (*n* = 4) tubercles.

**Figures 88–93.**
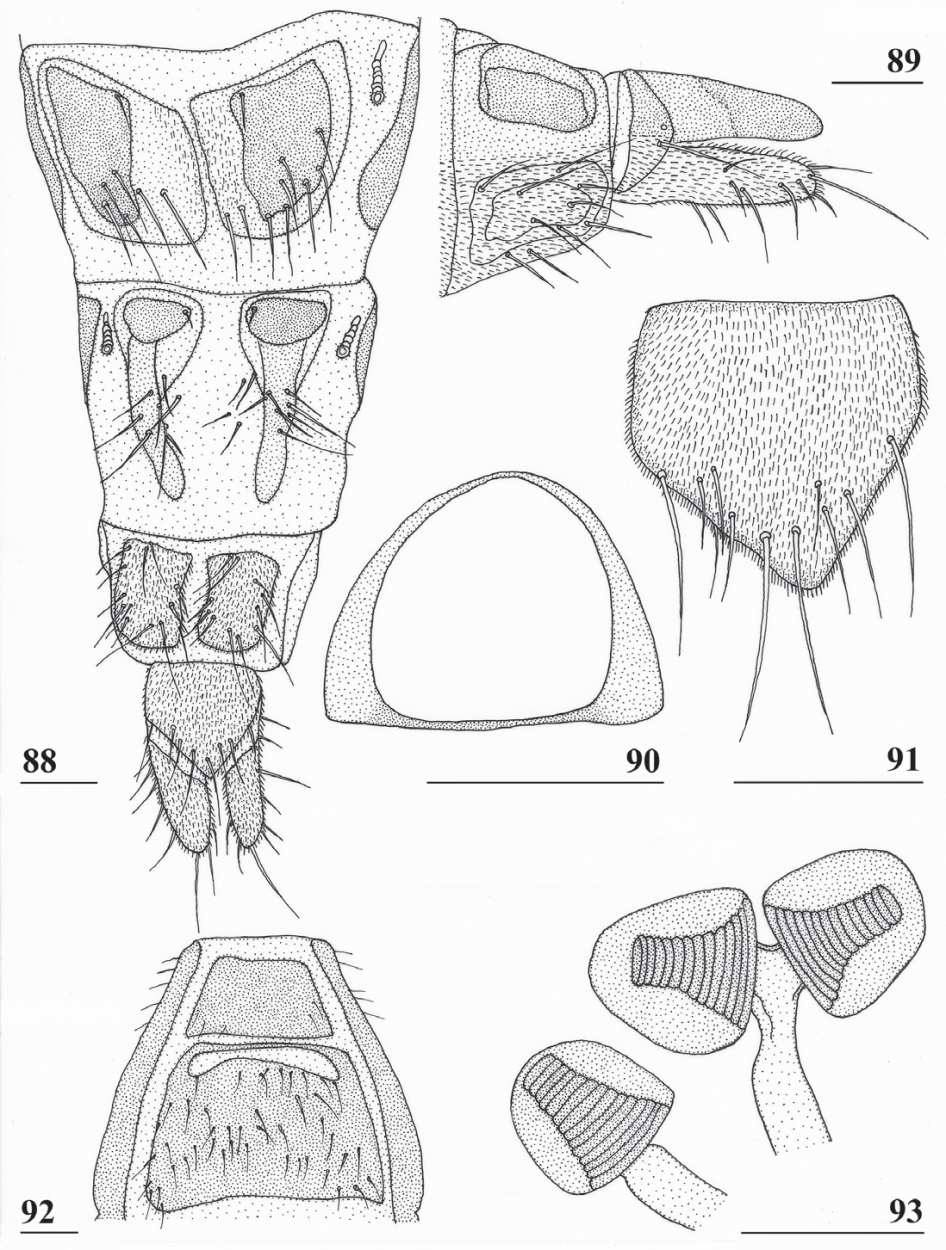
*Sphyracephalanigrimana*, ♀, Kedrovaya Pad, Russia **88** postabdomen, ventral view **89** tergite 8, 10 and cerci, dorsal view **90** sclerotised ring **91** subanal plate, ventral view **92** sternite 1, intersternite 1-2 and sternite 2, ventral view **93** spermathecae. Scale bars: 0.2 mm (**92**); 0.1 mm (**88–91, 93**).

***Preabdomen***. Tergites (Fig. 86) uniformly glossy, blackish brown, with scattered tiny white setulae, setulae laterally longer, darker and more dense; tergite 1 with distinct transverse ridges and vague, shallow circular groove, tergite 2 anteromedially with small triangle with transverse ridges; suture between tergites 1 and 2 distinct; sternites 1–6 glossy, dark brown, all covering the width of the abdomen, well covered with dark setulae; sternites 1 and 2 trapezoid (Figs 87, 92), sternite 1 glossy with a few small setulae, sternite 2 glossy, clothed in small setulae, intersternite 1-2 a solid, slender, darker sclerite, laterally broadening and connected to main sternite 2 (Fig. 92); sternites 3, 4 and 5 rectangular sclerites, sternite 3 as long as sternites 4 and 5 together; ♀ sternite 6 (Fig. 88) consisting of two rectangular sclerites well separated on the meson; ♂ sternite 6 (Fig. 99) represented by two small semi-circular sclerites, medially located.

***Female postabdomen***. Postabdomen narrow (Fig. 88); tergite 6 represented by two elongate, laterally located, sclerites; tergite 7 represented by two small, laterally located, sclerites; tergite 8 two elongate, pruinose sclerites, well separated on the meson (Fig. 89); tergum 10 short, triangular, thinly pruinose, one pair of apical setulae; cerci rather elongate, l/w ratio: ~ 3.3, clothed in microtrichia and setulae; sternite 7 consisting of two small, rounded, anteriorly located sclerites with long, narrow, less sclerotised posterior extensions (Fig. 88); spiracle 7 in membrane; sternite 8 represented by two large rectangular sclerites, separated on the meson, (Fig. 88), clothed in microtrichia and 12 pairs of setulae; subanal plate (Fig. 91) pentagonal with posterior corners rounded, apically with three pairs of long setulae, clothed in microtrichia and a few pairs of small setulae; spermathecae (Fig. 93) mushroom-shaped with inner structure large, striated, basally broadening cone-shaped, hollow and well sclerotised; sclerotised ring of ventral vagina, well developed, triangular to rounded, anterior side narrow (Fig. 90).

**Figures 94–99.**
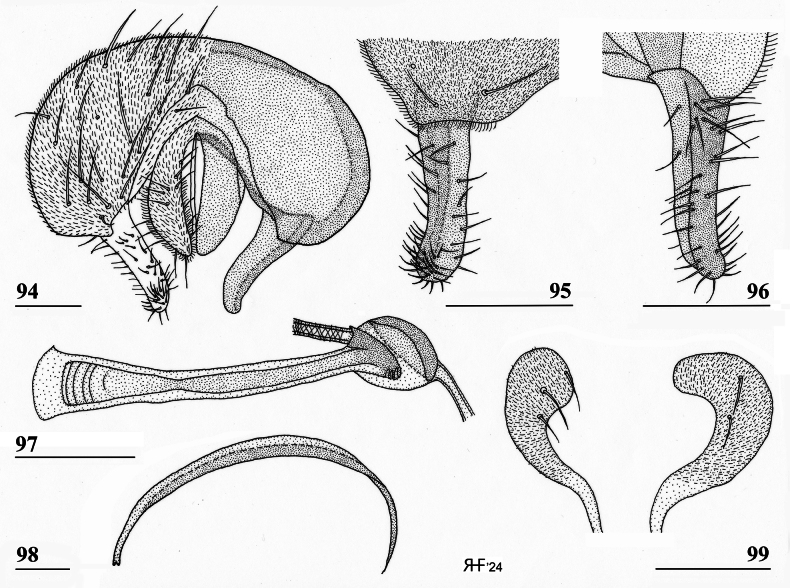
*Sphyracephalanigrimana*, ♂, Kedrovaya Pad, Russia **94** epandrium, cerci, surstyli, posterior view **95** surstylus, outer view **96** surstylus, inner view **97** ejaculatory apodeme + sac **98** syntergosternite 7+8, anterior view **99** sternite 8. Scale bars: 0.1 mm.

***Male postabdomen***. Syntergosternite 7+8 slender, on both sides extending to the venter, (Fig. 98), spiracles 7 in membrane; epandrium (Fig. 94) rounded, clothed in microtrichia and ~ 25 pairs of setulae; surstyli (Figs 95, 96) articulate, l/w ratio: ~ 4.7, almost parallel-sided, apically rounded, ventrally directed, on inner medial side hollow (scoop-like), no microtrichia, outer and inner sides clothed in setulae (Fig. 96); surstyli interconnected via slender processus longi; cerci rounded on lateral sides, length/broadest width ratio: 4.0, clothed in microtrichia and ~ 15 setulae; phallapodeme (lost during preparation) with slender anterior arm, lateral processes slender; ejaculatory apodeme straight, slender, apically ~ 3× as broad as basally (Fig. 97), ejaculatory sac normal-sized.

#### Biology.

The only observations on the biology of *S.nigrimana* are by Nartshuk (2017). She mentioned that the species is characterised by gregarious behaviour. More than 150 specimens were collected in one day in the Suputinsky Nature Reserve. They were found to be active from April to the end of October, while adults were assumed to hibernate. Mader (2017) mentions for *S.nigrimana* the antipodal position during copulation. All (also photographical) records for *Sphyracephala* and other diopsids show an epipodal position during copulation (e.g., Hochberg-Stasny 1985: figs 24–32). As, in addition, *S.nigrimana* was listed under European Diptera, this record by Mader can better be disregarded.

#### Distribution.

The type series originated from a tributary of the Amur River in Russia (Loew 1873). Bezzi (1922), Hennig (1941b), and Nartshuk (2017) all agreed that the flies from the vicinity (~ 43°08'N, 131°55'E) of Vladivostok and identified by Portschinsky (1871) as *S.brevicornis* belonged to *S.nigrimana*. Hennig (1941b) also recorded *S.nigrimana* from “der Mandschurei” (ZMHB). According to Pont and Ackland (2009), this locality is in Heilongjiang, China. Yang and Chen (1988) and Hua (2006) repeated this Chinese record without additional comments. Nartshuk (2017) specified that *S.nigrimana* is distributed in the Primorsky Territory to the north up to the Bikin-Belimbe line (up to ~ 46°48'N). She also mentions specimens from the Suputinsky Nature Reserve (~ 43°40'N, 132°30'E). Dubatolov (2020) reported on the presence of *S.nigrimana* in the more northern Bolshekhekhtsirsky Reserve (48°17'N, 132°49'E) near the Amur River. Flies were observed on the sunny wooden wall of the reserve office on 21.x.2020, 29.ix.2021 and 14.x.2021 [sic].

Liu (2009) listed one ♂ *S.nigrimana* from Hainan, China. From the same island he also recorded the Nearctic *S.brevicornis*. However, both records are based on misidentifications as can be verified, for instance, from the wing drawings. Biogeographically, these records would also have been highly unlikely.

The northern latitude limits of the Nearctic and Palaearctic *Sphyracephala* are quite consistent. The Nearctic species reach in Canada 47°36'N for *S.subbifasciata* and 45°30'N for *S.brevicornis* (Feijen 1989). The most northern record for *S.babadjanidesi* is found in Hungary with 46°41'N, while for *S.nigrimana* the northern limit in Russia comes to 48°17'N. Hibernation is found in all four Holarctic species.

## ﻿Discussion

### ﻿Sexual monomorphism and dimorphism in *Sphyracephala*

**Figure 100.**
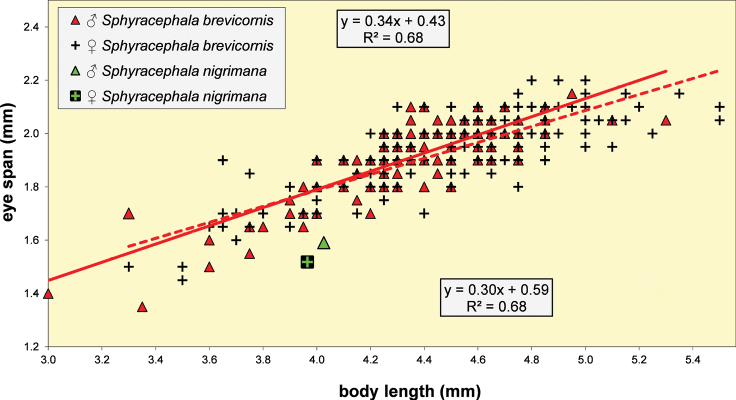
*Sphyracephalabrevicornis*, eye span plotted against body length. Older measurements for *S.brevicornis* from the 1970s and 1980s were also used. These measurements were not elaborated with the required accuracy for allometric studies. However, given the large number of data, the allometric lines obtained could be used, though the R^2^ values were rather low. Two data points for *S.nigrimana* are also presented.

**Figure 101.**
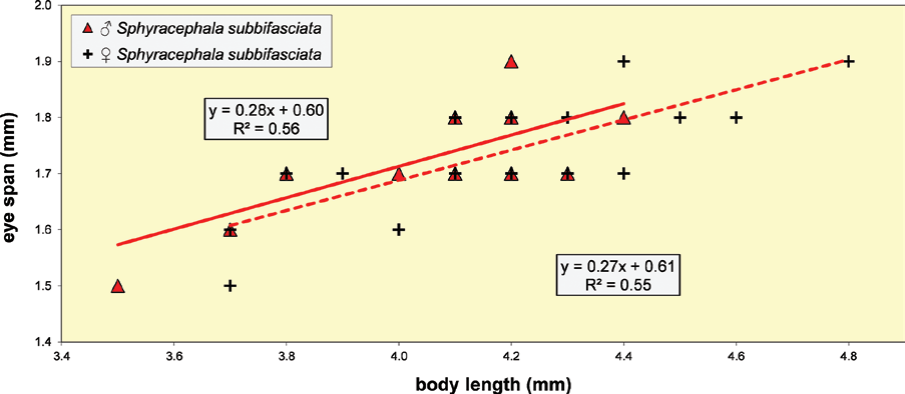
*Sphyracephalasubbifasciata*, eye span plotted against body length.

**Figure 102.**
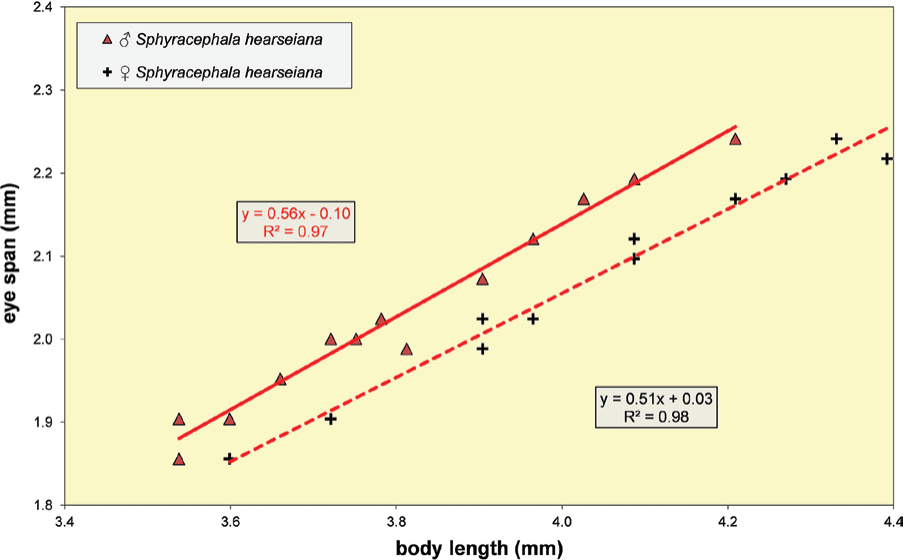
*Sphyracephalahearseiana* eye span plotted against body length.

**Figure 103.**
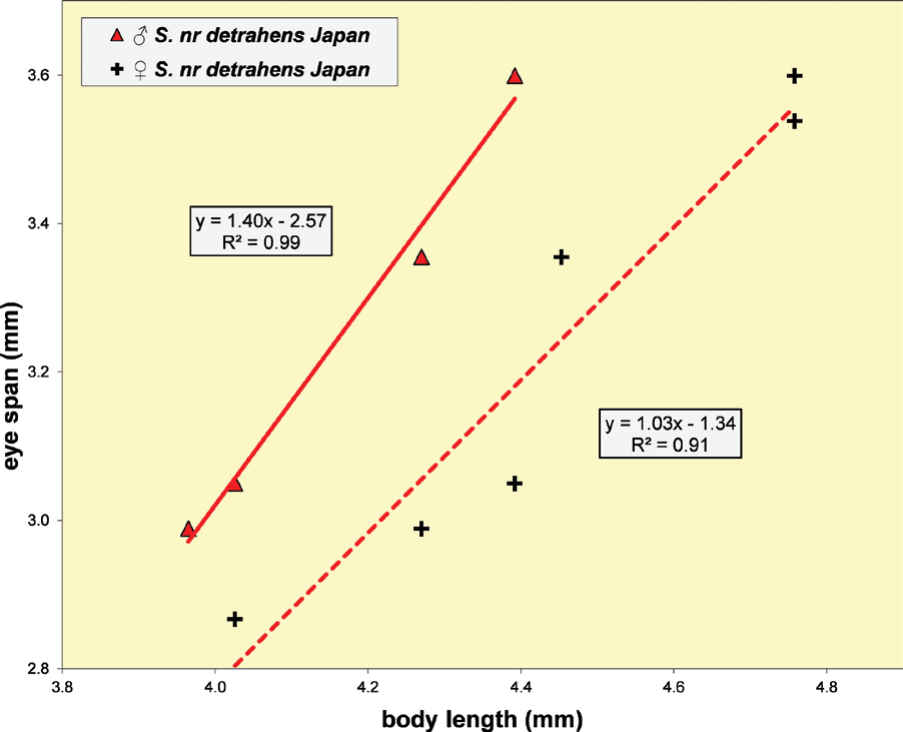
Sphyracephalanrdetrahens from Japan, eye span plotted against body length.

**Figure 104.**
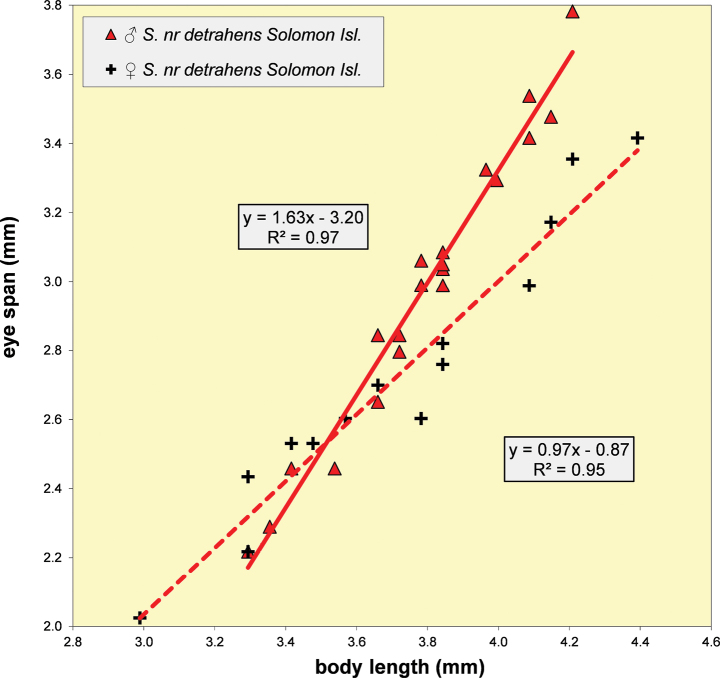
Sphyracephalanrdetrahens from Solomon Islands, eye span plotted against body length.

Allometric graphs for eye span on body length for *S.babadjanidesi*, *S.beccarii* and *S.munroi* have already been presented in their redescriptions. To get a more complete picture, we present graphs for *S.brevicornis* (Fig. 100), *S.subbifasciata* (Fig. 101), *S.hearseiana* (Fig. 102) and Sphyracephalanrdetrahens spp. from Japan and the Solomon Islands (Figs 103, 104). The two Nearctic species are both monomorphic with the smallest allometric slope value of all *Sphyracephala* (Table 2). For the East Asian *S.nigrimana* only two data points are available, but these point towards a close relationship with the Nearctic species (Fig. 100, Table 2). *Sphyracephalahearseiana* is also a distinct monomorphic species with a rate of dimorphism D = 0.05 (Fig. 102, Table 2). For the Oriental *S.detrahens* and *S.bipunctipennis* no allometric graphs could be constructed due to few datapoints of partly poor specimens. Baker and Wilkinson (2001) found for a “*S.bipunctipennis*” from Gombak River, Malaysia allometric lines of 0.377 ± 0.085 for ♀♀ and 1.114 ± 0.090 for ♂♂ indicating a dimorphic species with a D = 0.737. *Sphyracephalabipunctipennis* does not occur in Malaysia, but the species concerned must be a species near *S.detrahens*. Data for this species form an indication for the relatively high rate of dimorphy in the *S.detrahens*/*S.bipunctipennis* subtaxon. This is confirmed by allometric data for two Sphyracephalanrdetrahens from the Solomon Islands and Japan (Figs 103, 104, Table 2) with respectively D = 0.67 and D ≅ 0.37. The graph for the Japanese flies was only based on ten flies. Takeda et al. (2020: fig. 1a) presented a graph based on a much larger (224 ♀, 262 ♂) number of flies. They found allometric slopes of 0.917 ± 0.038 for ♀ and 1.14 ± 0.06 for ♂ and indicated the ♂ slope as significantly steeper than the ♀ slope. The dimorphism index came to D = 0.222, somewhat lower than our figure based on ten flies. The graph by Takeda et al. shows a rather high number of ♀ data points interspersed with male data points and vice versa. Determining the sex of flies in the *S.detrahens*/*S.bipunctipennis* subtaxon, we consider as rather difficult, certainly compared with all other diopsids.

**Figure 105.**
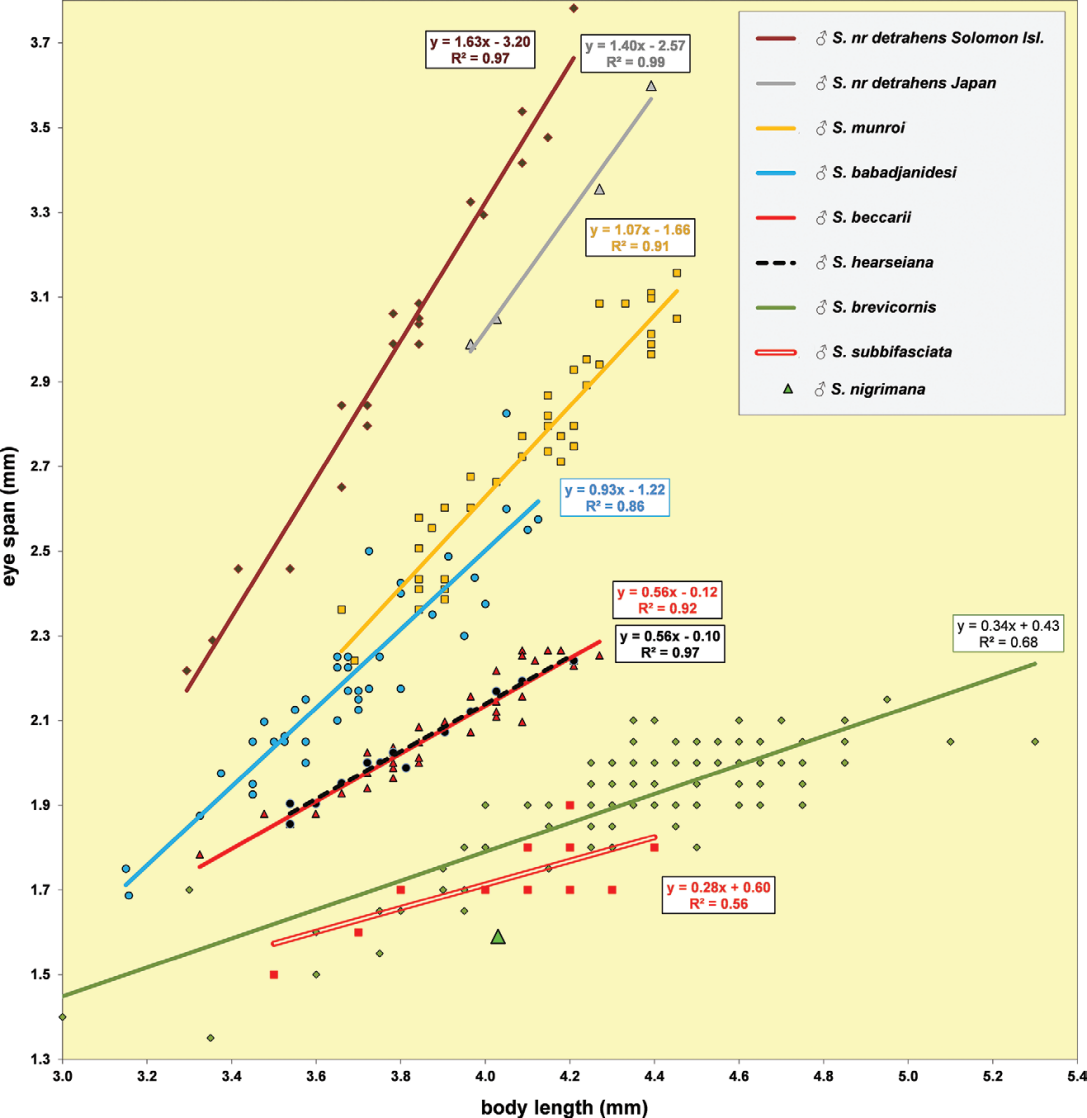
Eye span plotted against body length for males of eight *Sphyracephala* species. A single data point for *S.nigrimana* is also shown.

**Figure 106.**
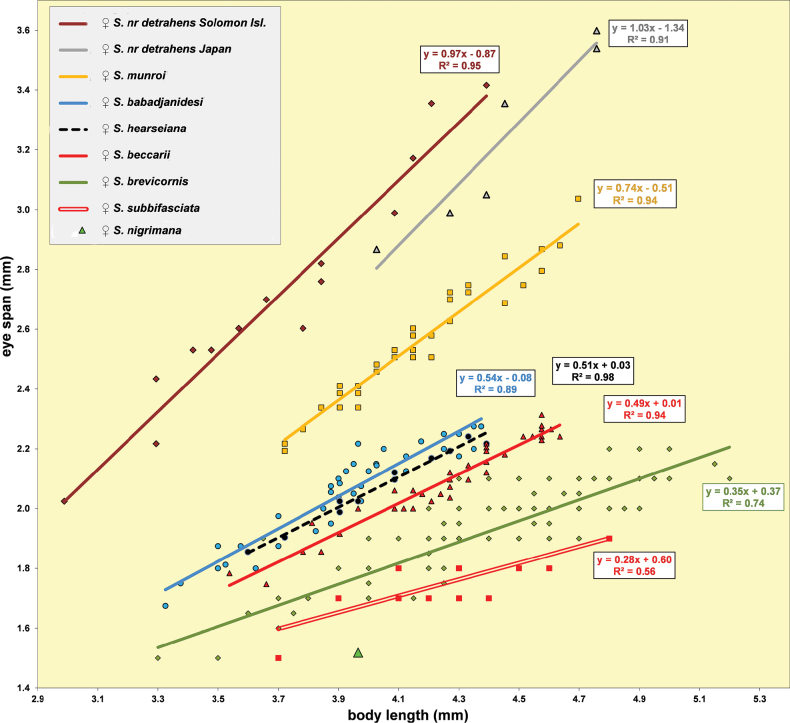
Eye span plotted against body length for females of eight *Sphyracephala* species. A single data point for *S.nigrimana* is also shown.

Comparison of the allometric lines (Figs 105, 106) for males and females of eight species gives a clear indication that species are not as closely related as appears from the general morphology. In general, allometric lines for related species, like in genera or species groups, are often quite similar. For females, lines are often almost overlapping, i.e., both slope and intercept are similar, while in males at least the slopes are comparable, giving parallel allometric lines. This can be demonstrated for the genera *Madagopsina* (Feijen et al. 2018: figs 155, 157), *Diopsina* (Feijen and Feijen 2013: fig. 56, table 1) and *Teleopsis* (Feijen 2011: table 1). However, from the male lines and especially the female lines for *Sphyracephala* species (Figs 105, 106), it is obvious that slopes and intercepts are quite different for the four subtaxa. This probably indicates that these taxa are old. The slopes of the lines are clearly linked to the subtaxa. In the graph for the males (Fig. 105) the two top lines are for the two dimorphic species of the *S.detrahens* group. This is followed by the lines for the dimorphic *S.munroi* and *S.babadjanidesi*. Remarkable is the case of the lines for the cryptic species *S.hearseiana* and *S.beccarii*. The lines for males are not just similar, but they fully coincide (Fig. 105). The bottom lines in the graph are for the two monomorphic Nearctic species. It should be noted that dimorphy in *Sphyracephala* must have developed independently in the *S.brevicornis* species group and the *S.hearseiana* species groups (see also the cladogram in Fig. 112).

### ﻿Geometric morphometric analysis

**Figure 107.**
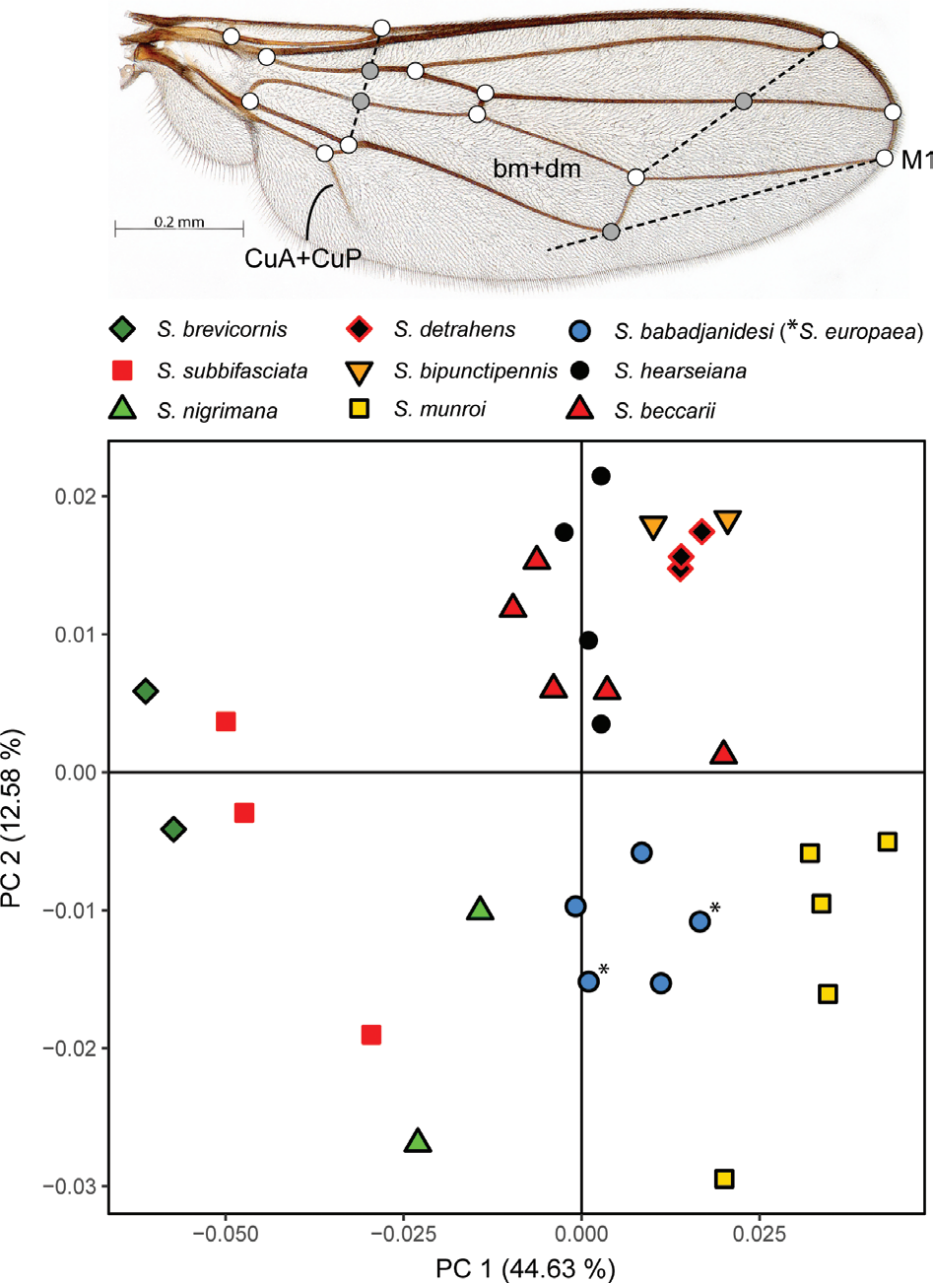
Geometric morphometric PCA analysis of *Sphyracephala* wing venation patterns. Grey dots represent landmarks that were placed with the aid of straight (dotted) lines through other landmarks (white dots). Asterisks indicate wings from paratypes of *S.europaea* (junior synonym of *S.babadjanidesi*).

The Geometric morphometric PCA analysis of wing venation patterns shows that 44.63% of variance is explained by PC 1 (Fig. 107) and *S.brevicornis*, *S.subbifasciata* and *S.nigrimana* are separated from the other species along this axis. *Sphyracephalamunroi and S.babadjanidesi* are found in the lower right of the PCA plot, separated from *S.hearseiana*, *S.beccarii*, *S.bipunctipennis* and *S.detrahens* along the second PCA axis (12.58% of variance, Fig. 107). The three *S.babadjanidesi* from Azerbaijan group together with the two *S.europaea* paratypes from Hungary (Fig. 107).

**Figure 108.**
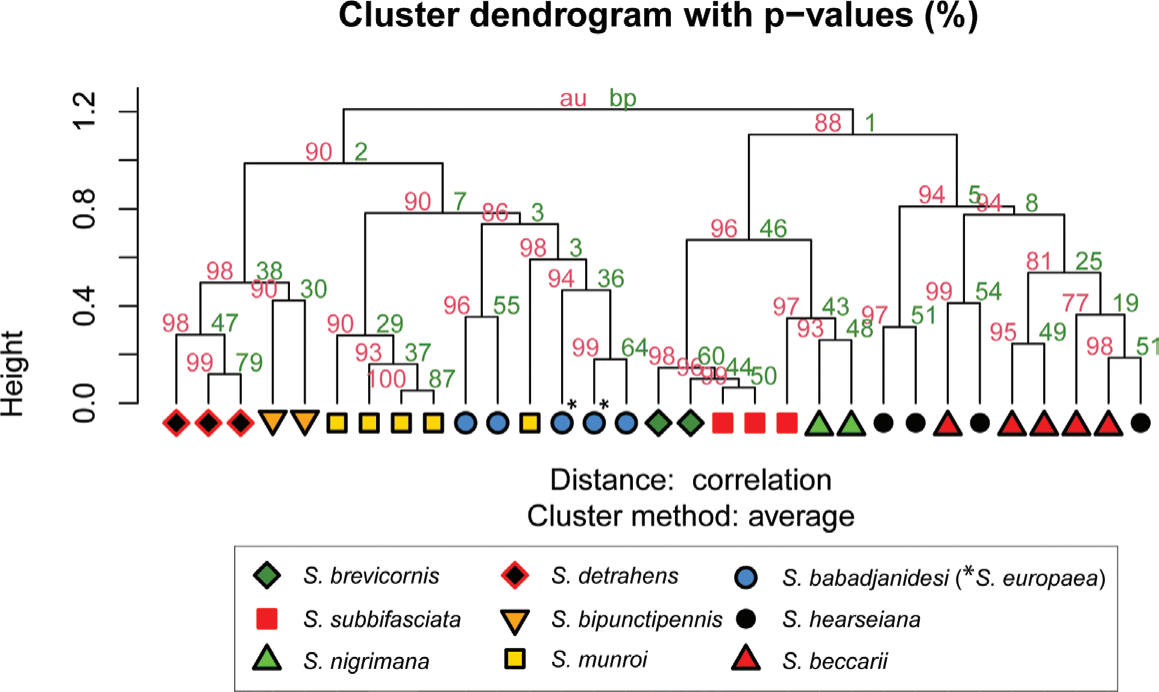
Hierarchical clustering analysis of principal component scores from the PCA in Fig. 107, using the average clustering method. Branch labels indicate the Approximately Unbiased p-value (AU) and Bootstrap Probability (BP). Asterisks indicate wings from paratypes of *S.europaea* (junior synonym of *S.babadjanidesi*).

**Figure 109.**
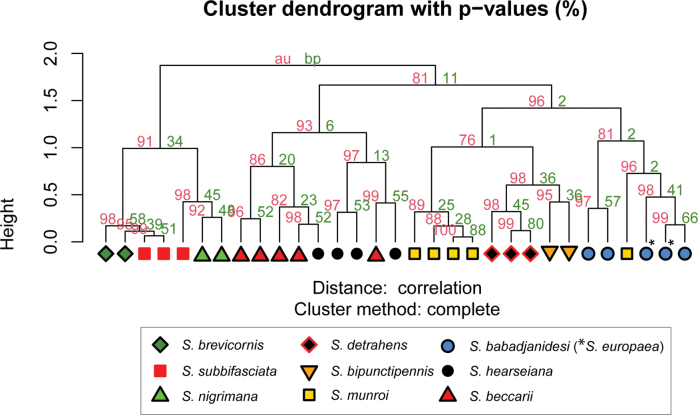
Hierarchical clustering analysis of principal component scores from the PCA in Fig. 107, using the complete clustering method. Branch labels indicate the Approximately Unbiased p-value (AU) and Bootstrap Probability (BP). Asterisks indicate wings from paratypes of *S.europaea* (junior synonym of *S.babadjanidesi*).

Both hierarchical clustering dendrograms show that the grouping of *S.brevicornis*, *S.subbifasciata* and *S.nigrimana* is robust to clustering method (Figs 108, 109). Likewise, both clustering methods group *S.beccarii* with *S.hearseiana* and *S.detrahens* with *S.bipunctipennis*. The species *S.babadjanidesi* and *S.munroi* are grouped together using the average clustering method (Fig. 108), but not all *S.munroi* specimens group with *S.babadjanidesi* when using the complete clustering method (Fig. 109). The two clustering methods do not lead to concordant clustering patterns among higher branches (Figs 108, 109).

In accordance with morphological and allometric evidence, geometric morphometric analysis supports the grouping of *S.brevicornis*, *S.subbifasciata* and *S.nigrimana*. Furthermore, *S.babadjanidesi* and *S.munroi* cluster together, *S.hearseiana* and *S.beccarii* cluster together and *S.bipunctipennis* clusters with *S.detrahens*. Support is found for *S.europaea* as junior synonym of *S.babadjanidesi* as their specimens cluster together. In our PCA analysis (Fig. 107), we see that the four species in the *S.hearseiana* species group (see Fig. 112) aggregate. However, this is not supported in the clustering analysis. The landmark data works well to identify which species are closely related but seems to be less informative to resolve grouping among more distant species, as both clustering dendrograms show different patterns (Figs 108, 109).

**Figures 110–112.**
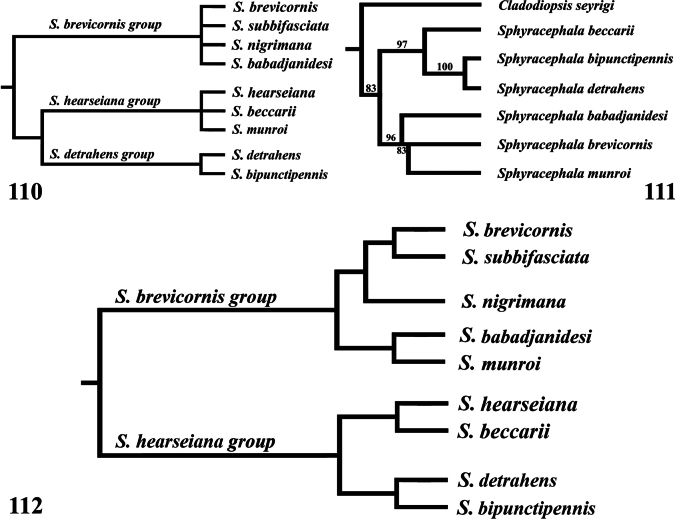
**110** Cladogram of *Sphyracephala* species groups according to Feijen (1989)**111** preferred maximum likelihood phylogeny of the Sphyracephalinae, based on molecular analysis (Jackson 2019, supplemental figure 1, in slightly adapted form). Numbers at nodes represent Ultrafast Bootstrap supports **112** revised cladogram of *Sphyracephala* species groups.

### ﻿Species groups in *Sphyracephala*

Feijen (1989) divided the *Sphyracephala* into three species groups based on morphological characters: the *S.brevicornis* species group and, as sister taxa, the *S.hearseiana* group and the *S.detrahens* (the former genus *Pseudodiopsis*) group. This view is presented in a cladogram (Fig. 110). The latest molecular analysis of *Sphyracephala* species is presented by Jackson (2019). A preferred maximum likelihood phylogeny of the Sphyracephalinae, extracted from Jackson’s supplemental fig. 1, is presented here in a slightly adapted form as Fig. 111. This molecular analysis did not involve *S.hearseiana*, *S.nigrimana* and *S.subbifasciata*. However, all sub-groups were presented and both analyses are largely in agreement, except for the placement of *S.munroi*. One of the main differential characters used by Feijen (1989) was the state of the prosternum: a basiliform prosternum in the *S.brevicornis* species group and a precoxal bridge in the other two groups. Based on the presence of a precoxal bridge in the Afrotropical *S.munroi*, this species was placed in the *S.hearseiana* group, together also with the Afrotropical *S.beccarii*. Examination of the state of the prosternum of *S.munroi* now made it clear that this species has a basiliform prosternum and should be placed in the *S.brevicornis* group. In dry specimens, the state of the prosternum can be difficult to ascertain, but re-examination of the prosternum of the two Afrotropical species (Figs 1, 2) clearly proved that these species belong in different species groups. As such, the phylogenies based on morphological characters and on molecular analysis can be brought into line, leading to a revised cladogram (Fig. 112). A small difference between the phylogenies based on molecular analysis on the one hand and morphological, allometric and wing morphometrics considerations on the other hand is found in the *S.brevicornis* species group. Jackson (2019) indicated *S.munroi* as probably more related to *S.brevicornis*, while the other approaches indicate it as the sister taxon of *S.babadjanidesi*.

The *S.brevicornis* species group can be recognised by the following set of character states: basiliform prosternum, tergite 1 with semicircular groove, ♀ sternite 8 with two small sclerites located on the meson, presence of sclerotised ring of ventral vagina wall, and surstylus without microtrichia. In the *S.hearseiana* species group these character states are precoxal bridge, tergite 1 with two longitudinal grooves, ♀ sternite 8 with two large, elongate plates, absence of sclerotised ring, and surstylus with microtrichia. For the differences between the subtaxa can be referred to the key to the species. Sets of quantitative characters also support the division of *Sphyracephala* in four subtaxa as presented in the revised cladogram (Tables 2, 3). In addition, wing morphometrics analysis supports the division into two species groups and four subtaxa (Fig. 112).

From a biogeographical point of view, the revised cladogram is consistent with the other approaches. The taxon of *S.brevicornis*, *S.subbifasciata* and *S.nigrimana* occurs in the Nearctic Region and Eastern Asia. These three monomorphic species have for females and males the lowest slopes for the allometric lines (Figs 105, 106). The other two species of the *S.brevicornis* group, *S.munroi* and *S.babadjanidesi* are dimorphic and respectively from the Afrotropical Region and from the Balkan and Caucasus Regions. A special case is formed by the sister taxa *S.beccarii* and *S.hearseiana* in the *S.hearseiana* species group, respectively occurring in the Afrotropical region and India-Pakistan. These monomorphic species cannot be distinguished by external morphological characters. Quantitative characters (Tables 2, 3) are also very similar, while the allometric lines simply coincide (Figs 105, 106). The geometric morphometric PCA analysis of wing venation (Fig. 107) and the cluster dendrograms (Figs 108, 109) confirm their close relationship. Nevertheless, male and female genitalia easily distinguish the two species (compare Figs 48, 49 with Figs 132, 133). Hennig (1941b) already remarked that the species are “außerordentlich ähnlich” (extraordinarily similar), but could be distinguished by the male genitalia. The final two species are the Oriental *S.detrahens* and *S.bipunctipennis*. These species were formerly placed in *Pseudodiopsis* and now form a subtaxon in the *S.hearseiana* species group. This subtaxon extends to Japan, Papua New Guinea and the Solomon Islands and contains several undescribed species. These dimorphic species are morphologically quite distinct from the other *Sphyracephala*.

### ﻿Sex ratios in *Sphyracephala*

Feijen and Feijen (2023) briefly reviewed sex ratios in Diopsidae, stating that “aberrant sex ratios, usually favouring the females, are often encountered (Burkhardt and de la Motte 1985; Feijen 1989; Wilkinson et al. 1998; Paczolt et al. 2017).” Burkhardt and de la Motte found that “sex ratio of freshly emerged dimorphic flies deviated significantly from the 1:1 ratio in favour of the females” while in “cultures of the homomorphic species no significant deviations were found”. Wilkinson et al. stated that “By comparing sex-ratio distributions in stalk-eyed fly (*Cyrtodiopsis*) progeny we found that female-biased sex ratios occur in species exhibiting eye-stalk sexual dimorphism and female preferences for long eye span.” Feijen (1989) already indicated that certainly not all Diopsidae, dimorphic with regards to eye span, have aberrant sex ratios. Johnsson (2015) discussed relations between eye span, sex ratio and meiotic drive in stalk-eyed flies. Paczolt et al. (2017) reviewed and studied the segregation distorter mechanisms in Diopsidae. Their results illustrated that sex ratio modification in these flies is “undergoing recurrent evolution with diverse genomic consequences.”

It has clearly been demonstrated that a link can occur between sexual dimorphism regarding eye span and female-biased sex ratios. However, many diopsids with distinct sexual dimorphy have 1:1 sex segregation like the well-known *Diopsislongicornis* Macquart and all species in the *Diopsisapicalis* species group. Feijen (1984) found a percentage for males of 52% (*n* = 24,162) for *D.longicornis* in the field, but flies reared from pupae showed a 50/50 sex ratio. However, he also reported on female-biased sex ratios in African *Diopsis*. So far, no monomorphic diopsids with aberrant sex ratios appear to have been reported, only Feijen (1989) indicated low male percentages for Nearctic *Sphyracephala*. It is interesting to examine the sex ratios in *Sphyracephala*. In this genus both male-biased and female-biased are found. In Table 4, an overview is presented for sex ratios in *Sphyracephala* species, showing remarkable variation in this respect.

Both Nearctic species, *S.brevicornis* and *S.subbifasciata*, sexually monomorphic regarding eye span, show a clear female-biased sex ratio. The percentages males come to respectively 39% and 36%. These data were based on flies in museum collections (Feijen 1989). Lavigne (1962) and Hochberg Stasny (1985) working on the biology of Nearctic *Sphyracephala* did not consider the sex ratio in flies collected or reared. Collecting new data in this respect would be useful. *Sphyracephalababadjanidesi* and *S.munroi* also belong to the *S.brevicornis* species group and both show low sexual dimorphism regarding eye span. Both species have a balanced sex ratio of close to 1:1 (Table 4).

*Sphyracephalahearseiana* and *S.beccarii* are sister species within the *S.hearseiana* species group. The former species shows a simple balanced sex ratio (Table 4), The large number of more than 1200 specimens of *S.beccarii* from the African continent and the Arabian Peninsula also gave a near perfect balanced sex ratio. However, the picture is very different for the more than 800 *S.beccarii* specimens collected in Madagascar over 84 malaise trapping periods and some additional sweepings. Here, almost twice as many females as males were found giving a sex ratio of 1.00:0.52 (Table 4). In Madagascar, *S.beccarii* is the only non-endemic diopsid (Feijen et al. 2018). Compared to the endemic species in Madagascar, some of which diverged more than 10 million years ago, it is likely that *S.beccarii* is a relatively recent introduction from continental Africa. Somewhere during this introduction, a segregation distorter must have been introduced. For the diopsids, this represents the first case of a female-biased sex-ratio in a geographically isolated population of a monomorphic species.

The remaining species in the *S.hearseiana* species group are *S.detrahens* and *S.bipunctipennis* (previously placed in *Pseudodiopsis*), and several undescribed species. For *S.detrahens* from Sulawesi and *S.bipunctipennis* from Sri Lanka, India, and Bhutan only few data are available (Table 4), but both might have a female-biased sex ratio. From undescribed species (S.nrdetrahens) from Japan and the Solomon Islands larger series of data are available. The more than 600 flies from Japan show a male-biased sex ratio, the percentage males coming to 60% (Table 4). A small sample of 44 flies from the Solomon Islands also had a male-biased sex ratio with 66% males. From the data presented in Table 4, it is obvious that the sex ratio issue is very diverse in *Sphyracephala*. The only conclusion that can be drawn is that the more closely related species show similar sex ratios.

### ﻿Morphological characters

Prosternum. The shape of the prosternum can form a major differential character in Diopsidae. In the Madagascar genera *Madagopsina* Feijen et al. and *Gracilopsina* Feijen et al. a basiliform prosternum occurs in the former genus and a precoxal bridge in the latter one. In *Sphyracephala*, the prosternum forms a major character to distinguish the two species groups. In the *S.brevicornis* species group only a basiliform prosternum occurs, while all species in the *S.hearseiana* species group have an apomorphic precoxal bridge (Figs 1, 2).

Tergite 1. A semi-circular groove occurs in tergite 1 of all species of the *S.brevicornis* species group (Fig. 64), while in the *S.hearseiana* species group tergite 1 has two longitudinal grooves (Fig. 42). It is interesting to note that in the *Sphyracephala*-like *Cladodiopsissicardi* Séguy also a semi-circular groove occurs.

Female sternite 8. In *S.beccarii*, female sternite 8 is represented by two small sclerites, almost touching on the meson near the genital pore (Fig. 4). Kumar (1978b) and Feijen (1989) described a similarly shaped sternite 8 for *S.hearseiana*, the sister species of *S.beccarii*. Kumar referred to sternite 8 as the “vulva”. In the other species of the *S.hearseiana* species group, sternites 8 form a similar structure, though less medially (Feijen 1989). In the species of the *S.brevicornis* group sternite 8 is represented by two large rectangular sclerites, well separated on the meson (Figs 3, 19, 88).

Sclerotised ring of ventral vagina wall. This sclerotised “ring” (see Kotrba 2000) of the ventral vaginal wall shows considerable variation in size and shape (Kotrba1995). In the subfamily Diopsinae, the sclerotised ring can form a useful character at genus- and species level (e.g., Presgraves et al. 1999; Feijen and Feijen 2012, 2013; Feijen et al. 2018), while in the Centrioncinae the ring does not occur (Feijen and Feijen 2023). For the Sphyracephalinae, the sclerotised ring has not yet been mentioned. Peterson (1987) and Feijen (1989) did not refer to the ring in Nearctic *Sphyracephala*, while Hilger (2000) also did not refer to the ring for Palaearctic species. Examination of the female postabdomens of *S.brevicornis* and *S.subbifasciata* revealed the existence of a small circular sclerotised ring with thin arms. In the *Sphyracephala*, redescribed, a small ring was also found in *S.babadjanidesi* (Fig. 22), while for *S.munroi* a tiny structure with thin lateral extensions was illustrated (Fig. 68). In *S nigrimana*, a large ring was found (Fig. 90). No sclerotised ring was found in *S.beccarii*, *S.hearseiana* and an S.nrdetrahens from Papua New Guinea. Examination of more species and specimens is required, but it appears likely that a sclerotised ring did develop in the *S.brevicornis* species group, but the ring is not found in the *S.hearseiana* species group. The absence of the ring can be considered a plesiomorphic condition.

Surstylus. The surstyli form an important differential character for the species. For the cryptic species *S.beccarii* and *S.hearseiana*, the shape of the surstylus is even the principal differential character. In the Nearctic species, the surstylus is fused to the epandrium, but the suture remains well visible. In the species of the *detrahens*/*bipunctipennis* group, the surstylus is seamlessly fused to the epandrium.

Quantitative characters. Tables 2 and 3 show the values for males and females of a set of quantitative characters for the *Sphyracephala* species: l/w ratio of fore femur, ratio eye span/ body length, allometric line for eye span on body length, ratio scutellar spine/scutellar length and ratio apical seta/scutellar spine. In these tables the species are arranged according to the four subtaxa distinguished. Examination of these data reveals that these quantitative characters can have a high taxonomic value, especially for the four subtaxa.

### ﻿An illustrated overview of the other five described *Sphyracephala* species

#### ﻿ Sphyracephala brevicornis

Taxon classificationAnimaliaDipteraDiopsidae

(Say, 1817)

0875BFC4-EF88-51E2-BBC1-5F65BC8F134C

Figs 100 , 105 , 106 , 107 , 108 , 109 , 110–112 , 113–115 , Tables 2 , 3 , 4

Diopsis brevicornis Say, 1817: 23.
Sphyracephala brevicornis (Say): Say 1828: plate LII (unpaginated). Portschinsky 1871: 287 (material from Siberia, misidentification for S.nigrimana); Curran 1934: 358 (in part, figure represents S.subbifasciata); Lavigne 1962: 5 (Lavigne - pers. comm. - later indicated that all data refer to S.subbifasciata, except for 1958 records); Sabrosky 1965: 638 (in part); Steyskal 1972: 13 (in part); Hochberg Stasny 1985: 1; Peterson 1987: 785 (in part); Feijen 1989: 72, figs 5–10, 13–40; Marshall 2017: 419, fig. 9 on p. 510. Up to 1989, authors considered S.subbifasciata as junior synonym of S.brevicornis. Their treatment of Nearctic Sphyracephala could therefore be one of the two species or a mixture.
Achias brevicornis (Say): Wiedemann 1830: 564. Wiedemann received these flies as A.brevicornis, but was convinced that they belonged to Diopsis.
Sphyracephala bicornis (Say): Peterson 1916: 183. Error for S.brevicornis.

##### Type series.

U.S.A.: ***holotype***, sex unknown, Wissahickon Creek near Philadelphia, Pennsylvania [~ 40°8'55"N, 75°13'14"W, ~ 60 m]. It seems likely that the single type specimen of *S.brevicornis* has been lost.

##### Distribution.

South-Eastern corner of Canada, contiguous U.S.A. east of the line Houston-Lincoln (Nebraska)-Grand Forks.

##### Illustrations.

**Figures 113–115.**
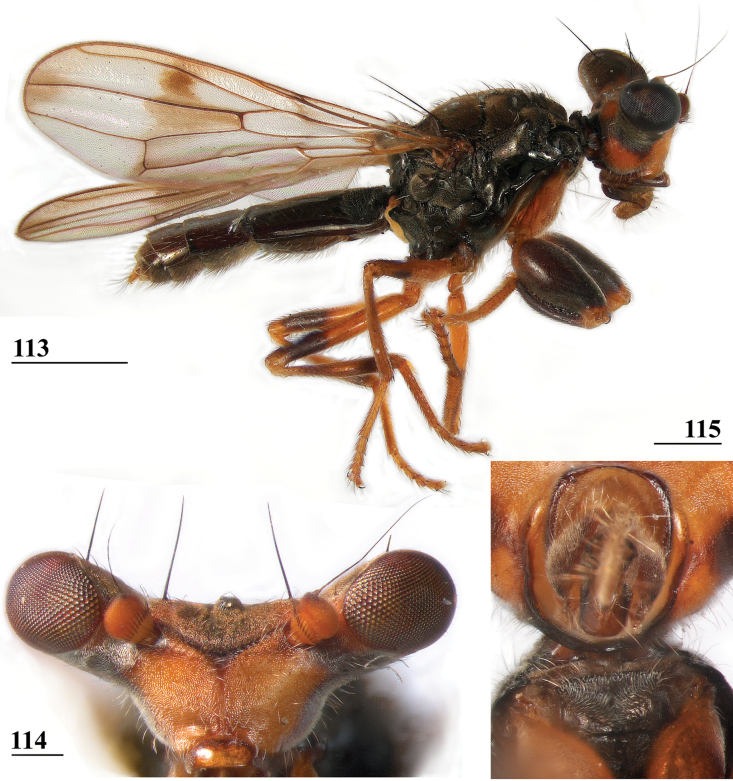
*Sphyracephalabrevicornis*, Belmont, U.S.A. **113** ♀, habitus, lateral view **114** ♂, head, anterior view **115** ♀, basiliform prosternum, ventral view. Scale bars: 1 mm (**113**); 0.2 mm (**114, 115**).

**Figures 116–118.**
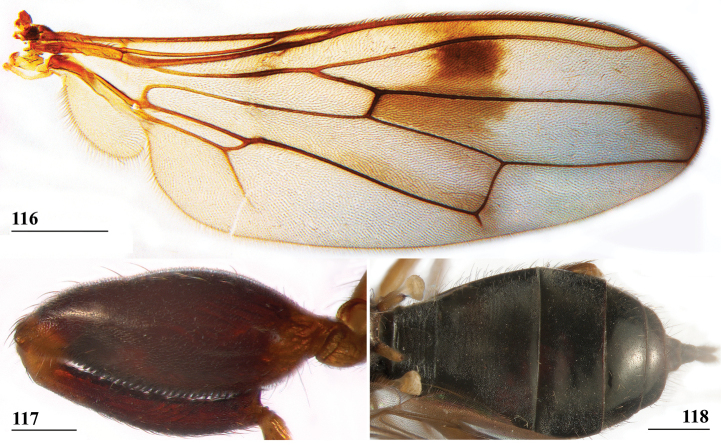
*Sphyracephalabrevicornis*, USA **116** ♀, Lewisburg, PA, wing **117** ♂, Belmont, fore femur, outer view **118** ♀, Philadelphia, abdomen, dorsal view. Scale bars: 0.5 mm (**116, 118**); 0.2 mm (**117**).

Feijen (1989) provided drawings of external morphology and genitalia. To this is added a set of photographs (Figs 113–118) of habitus, head, thorax, wing, fore femur, and abdomen.

#### ﻿ Sphyracephala subbifasciata

Taxon classificationAnimaliaDipteraDiopsidae

Fitch, 1855

B57F46D8-5C06-5007-9029-13690BF7674C

Figs 101 , 105 , 106 , 107 , 108 , 109 , 110 , 112 , 119–120 , 121–124 , Tables 2 , 3 , 4

Sphyracephala subbifasciata Fitch, 1855: 774. Williston 1908: 314 (S.brevicornis figure is S.subbifasciata); Curran 1934: 358 (S.brevicornis figure is S.subbifasciata); Lavigne 1962: 5 (Lavigne - pers. comm. - later indicated that all data refer to S.subbifasciata, except for 1958 records); Cole 1969: fig. 10; Barnes 1988: 110; Peterson 1987: 785 (fig. 61.1 represents S.subbifasciata); Feijen 1989: 84, figs 41–68; Delfosse 2006: 32 (S.brevicornis figure is S.subbifasciata); Stoaks and Shaw 2011: 232; Lonsdale 2013: table 2, figs 90, 91 (phylogenetic analysis), Londsdale 2020: 6, figs 143–147, 160–168, 188–190, 407; Marshall 2017: 419.
Sphyracephala brevicornis (Say): Loew 1873: 103. Loew was the first author to ascertain S.subbifasciata as junior synonym of S.brevicornis. Feijen (1989) indicated that Loew based his study of S.subbifasciata on a pair of flies received from Osten Sacken as S.subbifasciata. However, this pair represented S.brevicornis. Till Feijen (1989), all authors followed Loew’s view, while often reporting on a mixture of the two species, or one of the two species.

##### Type series.

USA: ***lectotype*** ♀, north of Ottawa, Illinois, 17.x.1854, swept from grass, at base of the bluffs of the Illinois river [~ 41°23'31"N, 88°47'13"W, ~ 150 m] (USNM, see also Feijen 1989: 84). ***Paralectotypes***: 2 ♀, same data as lectotype (USNM).

##### Distribution.

Feijen (1989) gave for distribution: South-Eastern Canada and Northern U.S.A. from Northern Colorado to New England. Stoaks and Shaw (2011) extended the known distribution 1200 km westward till Yellowstone National Park, Wyoming.

##### Illustrations.

**Figures 119, 120.**
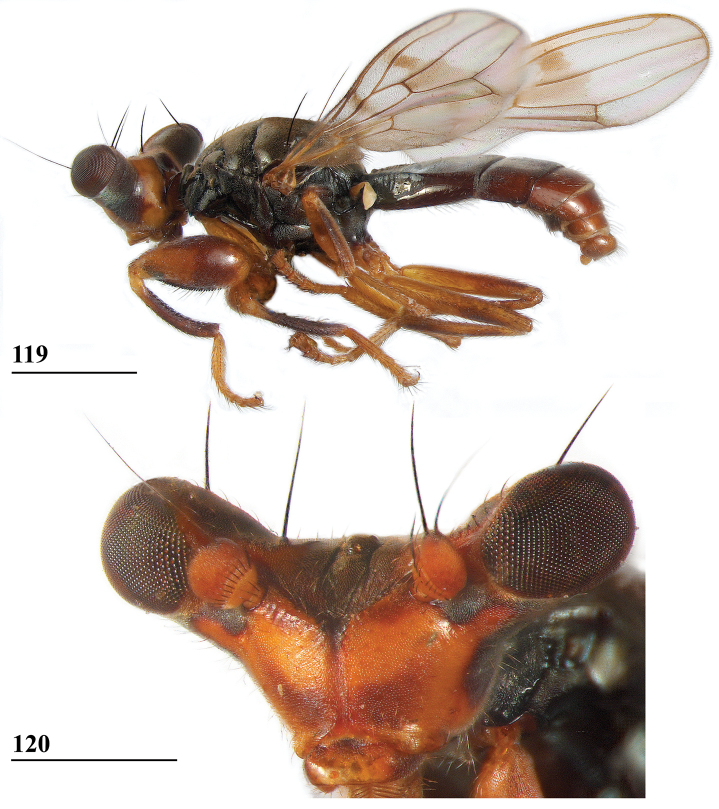
*Sphyracephalasubbifasciata*, Canada **119** ♀, Hull QC, habitus, lateral view **120** ♂, Hemmingford QC, head, anterior view. Scale bars: 1 mm (**119**); 0.5 mm (**120**).

**Figures 121–124.**
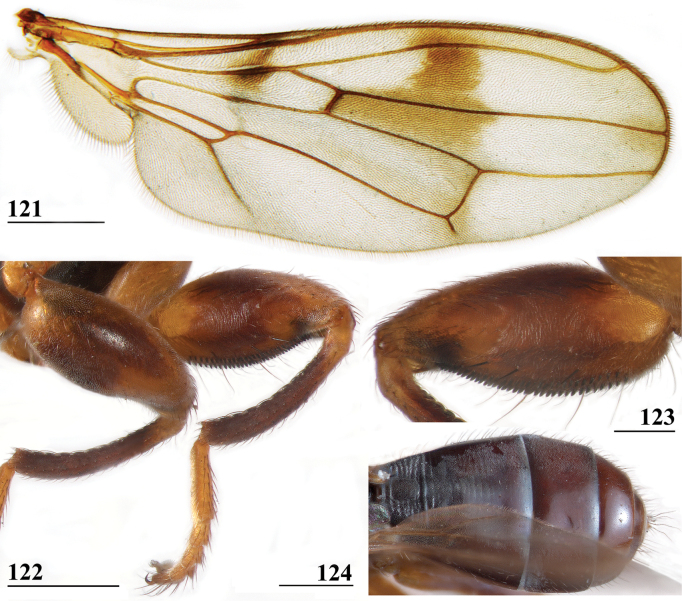
*Sphyracephalasubbifasciata*, Canada **121** ♀, Ottawa ON, wing **122** ♂, Hull QC, fore legs **123** ♂, Hull QC, fore femur, inner view **124** ♀, Hemmingford QC, abdomen, dorsal view. Scale bars: 0.5 mm (**121, 122**); 0.2 mm (**123, 124**).

Feijen (1989) and Lonsdale (2020) provided drawings of external morphology and genitalia, while Lonsdale also presented some photographs. To this is added photographs (Figs 119–124) of habitus, head, thorax, wing, fore femur, and abdomen.

#### ﻿ Sphyracephala hearseiana

Taxon classificationAnimaliaDipteraDiopsidae

(Westwood, 1845)

75C3DA03-D02F-5A53-857E-8458E48EB3F6

Figs 102 , 105 , 106 , 107 , 108 , 109 , 110 , 112 , 125–127 , 128–131 , 132–133 , Tables 2 , 3 , 4

Diopsis hearseiana Westwood, 1845: 99.
Sphryracephala hearseiana (Westwood): Westwood 1848: 37, pl. 18, fig. 3 (error for Sphyracephalahearseiana). Bigot 1892: 216; Sen 1921: 33.
Diopsis hoarseiana Westwood: Macquart 1851: 270, pl. 27, fig. 12.
Zygocephala hearsejana (Wiedemann): Rondani 1875: 443 (error for Zygocephalahearseiana (Westwood)), 1876: 184 (as Zygocephalahearseiana (Wiedemann)).
Sphyracephala hearseiana (Westwood): Loew 1873: 102. Hennig 1941a: 61; Kumar 1978a: 63, 1978b: 201, 1979a: 95, 1979b: 143; 70; Feijen et al. 2017: 85; Feijen and Feijen 2019: 40.
Sphyracephala hearseyana (Westwood): Osten Sacken 1882: 235. Van der Wulp 1896: 172; Brunetti 1907: 163; Hennig 1958: 567.
Sphyracephala hearseyiana (Westwood) (also as *hearseiyana*): Hennig 1941b: 5.
Sphracephala hearseyana (Westwood): Nayar and Tandon 1962a: 113. Nayar and Tandon 1962b: 131, 1963: 1; Singh et al. 1962: 79.
Non Sphyracephalahearseiana: Bezzi 1922: 69. African records are misidentifications for Sphyracephalabeccarii (Rondani).

##### Type series.

India: the type series appears lost. Westwood states “captured by Colonel Hearsey in different months and various localities; some on window-panes in June, some on orange and citron leaves in gardens in July, and some in the middle of August on cucumber leaves”. Westwood (1848) added “Inhabits Neemuch [Madhya Pradesh] and other parts of India”.

##### Distribution.

Some records are known for Islamabad and Punjab in Pakistan. Most records come from the western half of India from Himachal Pradesh to Tamil Nadu. As easternmost Indian locations a few records are found for Chhattisgarh, Odisha, and West Bengal. Datta and Biswas (1985) mention a record for Khushtia, in western Bangladesh. Two records are known from photographs of an *S.hearseiana*-like fly from Dan Chang and Ban Rai Districts in western Thailand (iNaturalist observations 110414296 and 112536071), but it remains to be seen whether these represent *S.hearseiana* or an undescribed species.

##### Illustrations.

**Figures 125–127.**
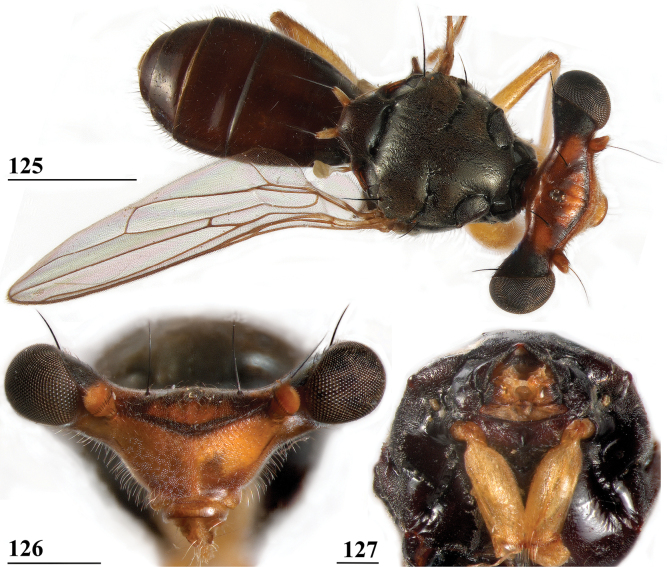
*Sphyracephalahearseiana*, ♀, **125, 126** Tamil Nadu, India **127** Madhya Pradesh **125** habitus, dorsal view **126** head, anterior view **127** precoxal bridge of prosternum, ventral view. Scale bars: 1 mm (**125**); 0.5 mm (**126**); 0.2 mm (**127**).

**Figures 128–131.**
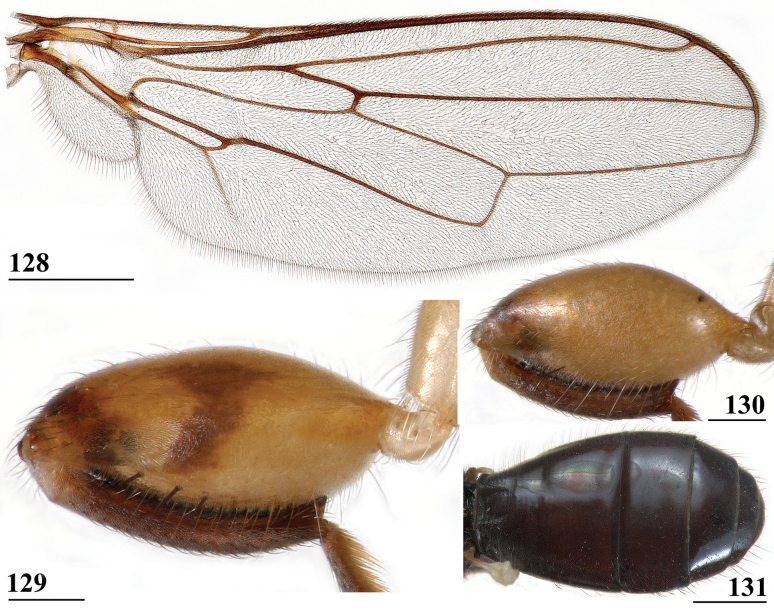
*Sphyracephalahearseiana*, Tamil Nadu, India **128** ♀, wing **129** ♀, fore femur, inner view **130** ♀, fore femur, outer view **131** ♂, abdomen, dorsal view. Scale bars: 0.5 mm (**131**); 0.2 mm (**128–130**).

**Figures 132, 133.**
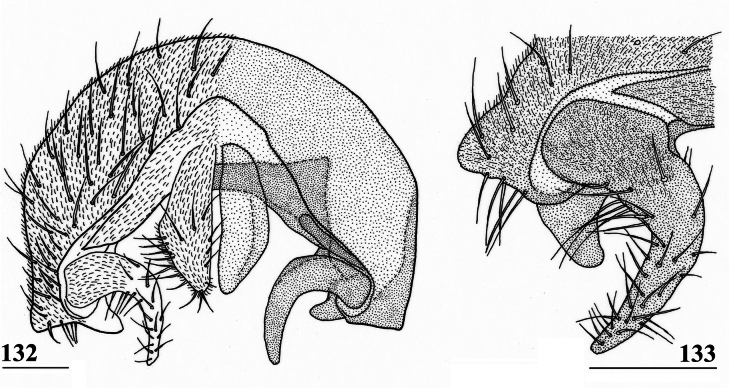
*Sphyracephalahearseiana*, Rajasthan, India **132** epandrium, cerci, surstyli, posterior view **133** surstylus, outer view. Scale bars: 0.1 mm.

Nayar and Tandon (1962a, 1962b, 1963) and Singh et al. (1962) provided drawings of wing, head, genitalia, and thorax, while Kumar (1978a, 1978b, 1979a, 1979b) gave drawings of genitalia, head, and mouthparts. Feijen and Feijen (2019) gave photographs of habitus and head. Here, photographs (Figs 125–131) of habitus, head, thorax, wing, fore femur, and abdomen are presented and drawings of male genitalia (Figs 132, 133).

#### ﻿ Sphyracephala detrahens

Taxon classificationAnimaliaDipteraDiopsidae

Walker, 1860

6D23E0AA-7D8E-5A79-A676-DC04E972399B

Figs 107 , 108 , 109 , 110–112 , 134–136 , 137–141 , Tables 2 , 3 , 4

Diopsis detrahens Walker, 1860: 161. Descamps 1957: 18; Steyskal 1972: 8.
Diopsis cothurnata Bigot, 1874: 115.
Pseudodiopsis detrahens (Walker): Hennig 1965: 60 (with P.cothurnata as junior synonym). Steyskal 1977: 35 (with P.cothurnata as junior synonym).
Pseudodiopsis cothurnata (Walker): Hendel, 1917: 33. Curran 1934: 495; Malloch 1938: 437; Shillito 1940: 150.
Microdiopsis cothurnata (Walker): Curran, 1934: 359. Curran 1934: 495 (in the corrections rectified to Pseudodiopsiscothurnata); Malloch 1938: 437; Steyskal 1972: 12.
Sphyracephala detrahens (Walker): Feijen 1989: 66. Baker 1999: 24, fig. 1-2; Meier and Baker 2002: 326.

##### Type series.

Indonesia: ***holotype*** “*Fæm.*” ♀ (head and abdomen lost, Fig. 136), Makessar, Celebes, on label Macassar, Celebes, A. R. Wallace, ex coll. Saunders 684 [Makassar, Sulawesi, Indonesia, ~ 5°12'31"S, 119°27'1"E, ~ 5 m] (NHMUK). The type of *D.cothurnata* appears lost. Bigot only indicated Célèbes [Sulawesi, Indonesia].

**Figures 134–136.**
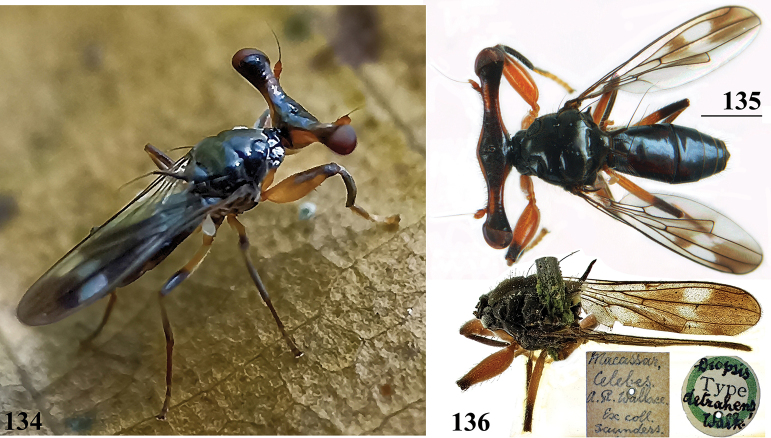
*Sphyracephaladetrahens***134** Donggala, Sulawesi, Indonesia (photograph © Ariyo Prasetyo) **135** ♀, Dumoga, Sulawesi, habitus, dorsal view **136** ♀, holotype, Makassar, Sulawesi, dorsolateral view. Scale bar: 1 mm (**135**).

##### Distribution.

Only Sulawesi can, at present, be considered as the area for *S.detrahens*. *Sphyracephala* specimens from countries as far apart as Malaysia, Japan, and the Solomon Islands have been identified as *S.detrahens*. However, at least part of these identifications appears doubtful and more study of genitalia or molecular studies are required. Although *S.detrahens* and *S.cothurnata* appear distinct synonyms, it is possible that at least on the small islands near Sulawesi an additional species occurs.

##### Illustrations.

A set of photographs (Figs 134–141) of habitus, head, thorax, wing, fore femur, and abdomen is presented.

**Figures 137–141.**
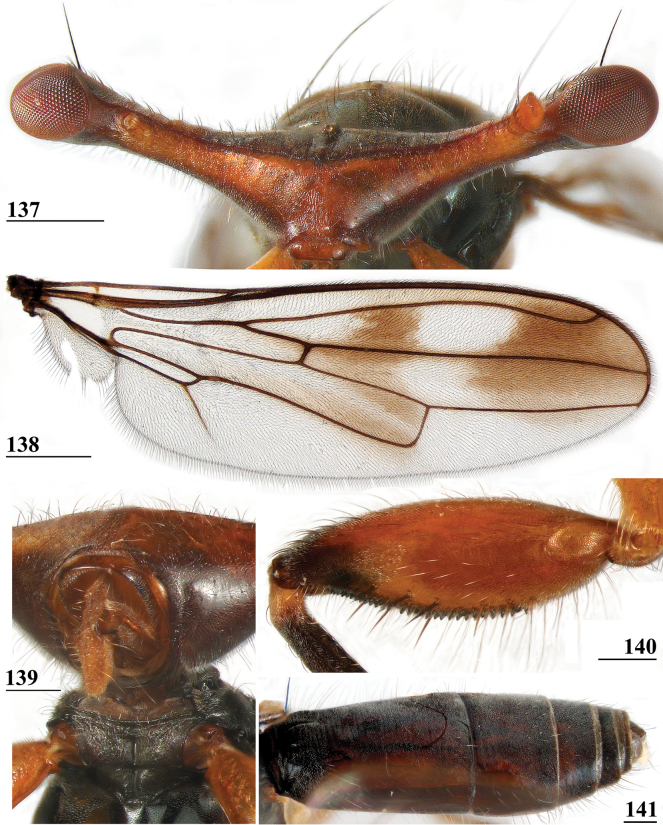
*Sphyracephaladetrahens*, ♀, Dumoga, Sulawesi, Indonesia **137** head, anterior view **138** wing **139** precoxal bridge of prosternum, ventral view **140** fore femur, inner view **141** abdomen, dorsal view. Scale bars: 0.5 mm (**137, 138**); 0.2 mm (**139–141**).

#### ﻿ Sphyracephala bipunctipennis

Taxon classificationAnimaliaDipteraDiopsidae

Senior-White, 1922

40BA5D15-51B7-5867-BA90-05B32AB0F9D0

Figs 107 , 108 , 109 , 110–112 , 142–146 , 147–150 , Tables 2 , 3 , 4

Teleopsis bipunctipennis Senior-White, 1922: 165, pl. 13, fig. 1. Descamps 1957: 19; Steyskal 1972: 11.
Pseudodiopsis bipunctipennis (Senior-White): Shillito 1940: 150. Steyskal 1977: 35.
Sphyracephala bipunctipennis (Senior-White): Feijen 1989: 67. Feijen 1998: 50; Meier and Hilger 2000: 4 (specimens from Thailand, unlikely to be S.bipunctipennis); Baker et al. 2001: 93, fig. 1 (specimens from Malaysia, unlikely to be S.bipunctipennis); Meier and Baker 2002: 334 (specimens from Malaysia, unlikely to be S.bipunctipennis); Feijen and Feijen 2019: 39; Jackson 2019: suppl. fig. 1 (specimens from Malaysia, unlikely to be S.bipunctipennis).

##### Type series.

Sri Lanka: ***holotype*** ♂, Ceylon, Indiganga [on label], on plant growing in the water at edge of the Suduganga river, on leaves of Liliacrans plant [on labels], 10.viii.1919 [~ 7°29'22"N, 80°39'46"E, ~ 380 m] (NHMUK). ***Paratypes***: 7 ♀, 4 ♂, same data as holotype (NHMUK). Senior-White mentioned in description “Type, allo-type, and ten co-types”.

##### Distribution.

Sri Lanka, India (Karnataka, Tamil Nadu), ?Bhutan. Specimens from Bhutan still require confirmation.

##### Illustrations.

**Figures 142–146.**
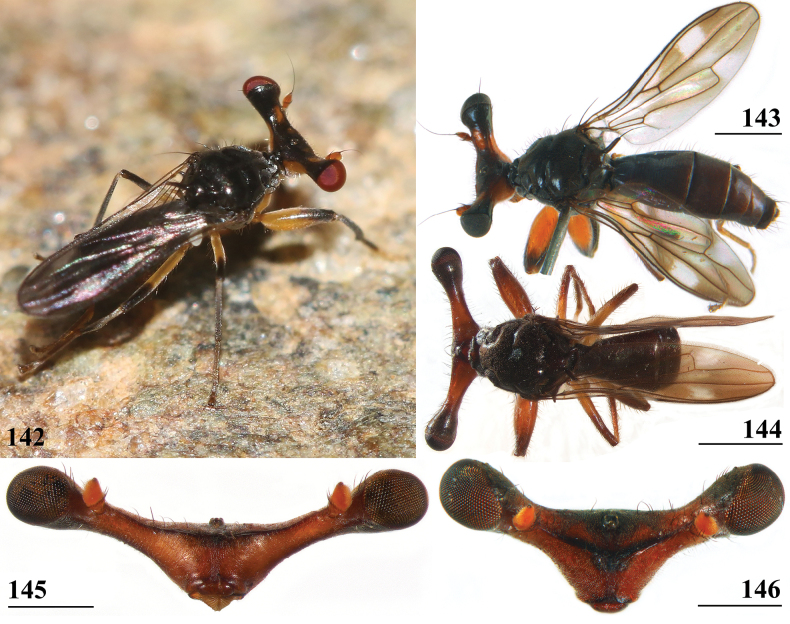
*Sphyracephalabipunctipennis***142** Matale, Sri Lanka (photograph © Amila P. Sumanapala) **143** ♀, Tamil Nadu, India, habitus, dorsal view **144** ♂, paratype, Indiganga, Sri Lanka, habitus, dorsal view **145** ♂, paratype, head, anterior view **146** ♀, paratype, head, anterior view. Scale bar: 1 mm (**143**, **144**); 0.5 mm (**145**, **146**).

**Figures 147–150.**
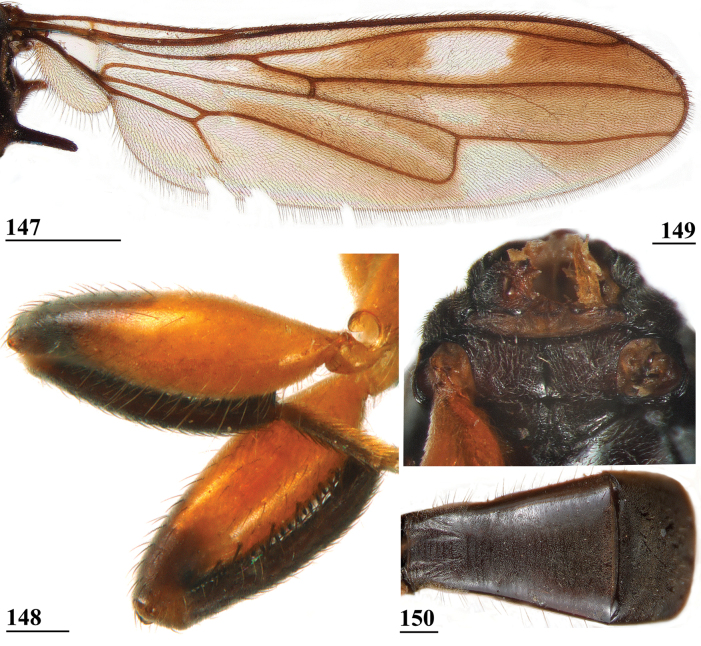
*Sphyracephalabipunctipennis*, **147**, **149**, **150** ♂, paratype, Indiganga, Sri Lanka, **148** ♀, Tamil Nadu, India **147** wing **148** fore femora, lateral view **149** precoxal bridge of prosternum, ventral view **150** abdomen, dorsal view. Scale bars: 1 mm (**147**); 0.2 mm (**148, 150**); 0.1 mm (**149**).

Senior-White (1922) gave a schematic drawing of the wing. Meier and Hilger (2000: figs 12–17, 115, 116, 118) presented photographs of the egg, but these are unlikely to represent *S.bipunctipennis*. Feijen and Feijen (2019) gave photographs of habitus and head. Life photographs are available (e.g., https://www.inaturalist.org/observations/53700018 © Amila P. Sumanapala). Here, photographs (Figs 142–150) of habitus, head, thorax, wing, fore femur, and abdomen are presented.

## Supplementary Material

XML Treatment for Diopsidae

XML Treatment for Sphyracephala

XML Treatment for Sphyracephala babadjanidesi

XML Treatment for Sphyracephala beccarii

XML Treatment for Sphyracephala munroi

XML Treatment for Sphyracephala nigrimana

XML Treatment for Sphyracephala brevicornis

XML Treatment for Sphyracephala subbifasciata

XML Treatment for Sphyracephala hearseiana

XML Treatment for Sphyracephala detrahens

XML Treatment for Sphyracephala bipunctipennis

## ﻿Additional information

### Author ORCIDs

Hans R. Feijen https://orcid.org/0000-0002-7265-8448

Frida A. A. Feijen https://orcid.org/0000-0001-5872-8780

Cobi Feijen https://orcid.org/0000-0001-8756-3903

### Data availability

All of the data that support the findings of this study are available in the main text.
